# Decoding circular polarization with biaxially chiral fluorescence reporters

**DOI:** 10.1039/d6sc05555a

**Published:** 2026-07-31

**Authors:** Summer J. Brown, Jiaoyan Zhao, Delia K. Savin, Bella S. Davey, Cedric P. Schaack, Austin M. Evans

**Affiliations:** a Department of Chemistry, University of Florida Gainesville Florida 32611 USA austinevans@ufl.edu; b Department of Chemistry, Wake Forest University Winston-Salem North Carolina 27109 USA schaacc@wfu.edu; c Department of Materials Science and Engineering, University of Florida Gainesville Florida 32611 USA; d Center for Functional Materials, Wake Forest University Winston-Salem North Carolina 27109 USA

## Abstract

Chiroptical organic materials that interact asymmetrically with circularly polarized light will drive advancements in quantum cryptography, biosensing, and anti-counterfeiting. In this report, we show how biaxial chiral emitters can be used to decode the circular polarization of light. Specifically, we develop a library of contorted dithienophenazine chromophores that have a donor–acceptor–donor architecture, which we can straightforwardly access as both enantiomers (*R*/*S*). The tailored donor–acceptor interactions in this scaffold provide tunable absorption and emission across a broad range of wavelengths. These optical properties are retained in polymer-based films, which suggests that these molecular chromophores could be deployed in solid-state devices. Density functional theory reveals that installing increasingly electron-rich substituents to the dithienophenazine core significantly decreases the HOMO–LUMO gap. Chiral donor–acceptor–donor chromophores are sensitive to solvent polarity, protonation, and temperature, which provides access to emission across a >100 nm range. We find that the fluorescence intensity of these atropoisomeric chiral compounds depends on the circular polarization of the excitation source. This differential emission intensity can be increased by aggregation induced emission, which leads to fluorescence quantum yields exceeding 50%. Overall, we leverage this class of biaxially chiral chromophores to create a tunable library of fluorescent chiroptical materials that can be used to decode information stored in the spin angular momentum of photons.

## Abstract 2

Chiroptical organic materials that interact asymmetrically with circularly polarized light will drive advancements in quantum cryptography, biosensing, and anti-counterfeiting.

## Graphical abstract

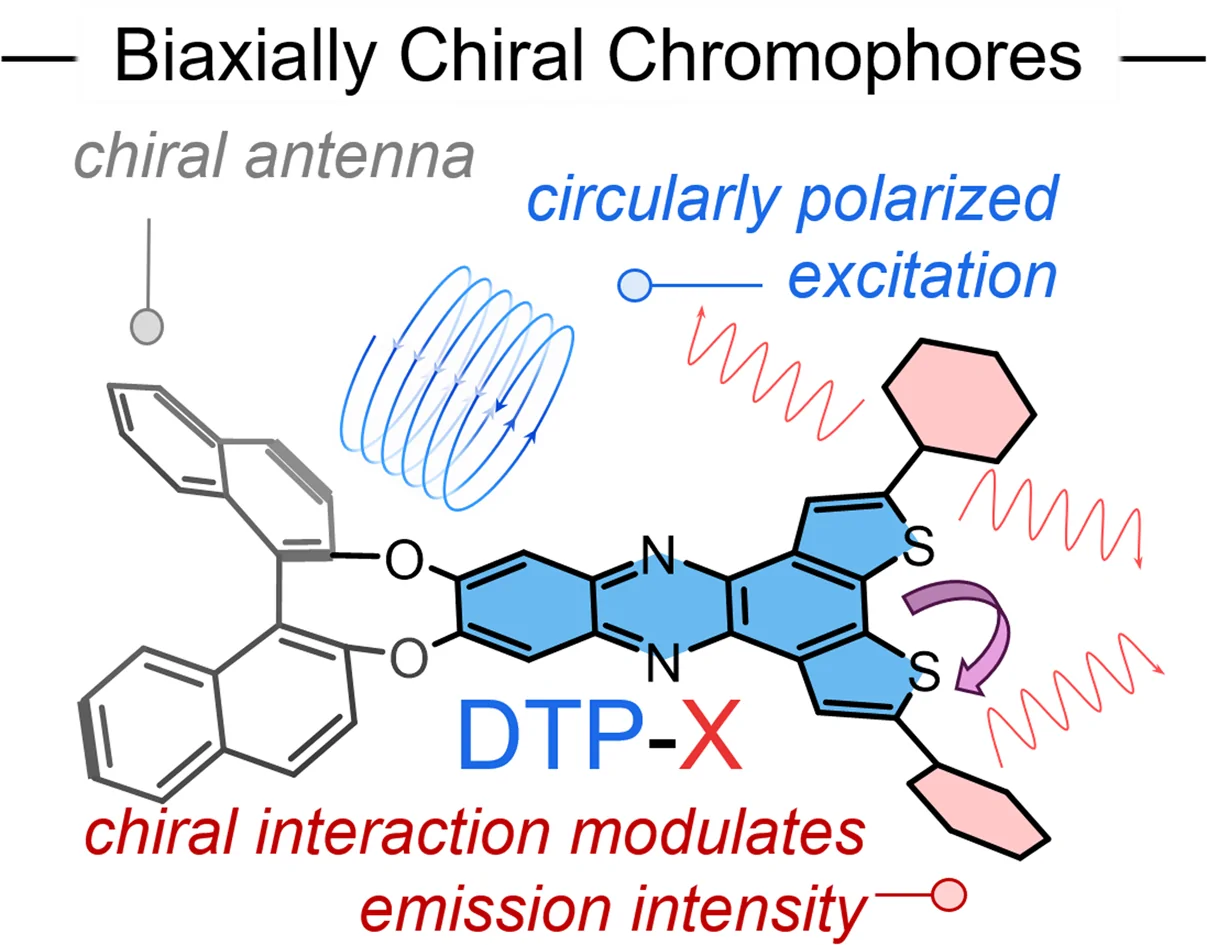

## Introduction

Manipulating and measuring the circular polarization of light will provide new opportunities in biosensing,^1–3^ spintronics,^4–8^ energy-efficient displays,^9–11^ anti-counterfeiting,^12–14^ and quantum cryptography.^15–18^ Circular polarization arises from spin angular momentum in bosonic photons, which takes on values of either −ℏ or +ℏ for left-handed or right-handed circular polarization (LCP and RCP).^19^ Chiral molecules and nanostructures interact asymmetrically with LCP and RCP because their directional electric potential couples with the rotating electric and magnetic field vectors of circularly polarized light.^20,21^ As one example of these phenomena, Robinson and coworkers exploited these chiral light–matter interactions in inorganic semiconductor nanocrystals that form helical nanostructures in thin films, which lead to highly preferential circular dichroism (CD).^22^ CD spectrometers remain bulky instruments that struggle to balance overall molecular absorption against the much smaller circular dichroism signal. This reliance on complex instrumentation and the low signal-to-noise ratio of this technique has relegated CD-based phenomena primarily to passive structural analysis or filter design, limiting their deployment in active encryption or sensing contexts.

Luminescence-based chiral materials are more attractive for active photonic, electronic, and quantum technologies. Luminescence offers a higher signal-to-background ratio because it tracks the light emitted by the chiral element rather than measuring small absorption differences relative to an external source.^23^ Significant effort has been devoted to developing chiral organic emitters with optical properties that can be readily tuned through synthetic design.^24–28^ Direct detection of circularly polarized emission is attractive, but state-of-the-art chiral organic emitters suffer from challenges associated with optimizing *g*_lum_ (luminescence dissymmetry factor) values and photoluminescence quantum yield concurrently.^29^ A related strategy to detect the circular polarization of an excitation source relies on preferential CD absorption, which then leads to a differential emission intensity when these molecules are fluorescent. In these fluorescence-detected circular dichroism methods, the chiroptical absorption and achiral emission can be decoupled, which circumvents tradeoffs inherent to modulating chiral emission directly.^30–33^ For fluorescence-detected circular dichroism methods to mature, it is necessary to develop chiral chromophore structure–property design rules for optical characteristics such as absorption wavelengths (*λ*_abs_), absorptivity (*ε*), photoluminescence quantum yield (*Φ*_emission_), and emission color (*λ*_emission_).

Recent efforts have focused on pairing electron-rich donors with electron-deficient acceptors along a chiral axis to generate chiroptically active materials.^34–36^ For instance, Jin and coworkers developed an efficient synthesis of chiral donor–acceptor (D–A) helicenes that exhibit a high transition magnetic dipole moment and, by extension, high dissymmetry factors (*g*).^35^ This design approach has allowed them to bias the product ratios of the resultant helicenes, and the D–A characteristics manifest as high *Φ*_emission_ and pronounced CPL. However, these donor–acceptor chromophores frequently require complex syntheses and small-scale (10 mg) chiral purifications, which complicate systematic investigations of their structure–property relationships and limit their application relevance. We recently reported a biaxial chromophore scaffold that addresses this practical limitation.^37^ We hypothesized that introducing donor–acceptor–donor (D–A–D)^36,38,39^ interactions into this biaxial chromophore platform would let us tune chiroptical absorption and emission independently, circumventing the conventional inverse correlation between *g*_lum_ and fluorescence efficiency.^23^

In this report, we show that a biaxially chiral D–A–D scaffold enables simple decoding of the circular polarization of light using only luminescence intensity measurements. We first synthesized enantiopure chromophores derived from a central dithienophenazine core.^40^ Pairing an electron-deficient phenazine motif with an electron-rich dithiophene flanking unit provides a base-level push–pull chromophore, which we deliberately augment by functionalizing the periphery of this scaffold.^41,42^ By installing increasingly electron-rich units, we red-shift the absorption and increase the molar absorptivity of these compounds. Additionally, the highly polarized D–A–D structure of these molecules renders their *λ*_emission_ sensitive to solvent polarity. Crucially, these optical properties are retained when the chromophores are embedded in solid-state thin films, underscoring their viability for practical device integration. We also find that the most emissive of these chromophores undergoes significant aggregation-induced emission (AIE) with *Φ*_emission_ that evolves from <1% to >50% when concentrated. Although the g_lum_ of these materials is below observable levels (<10^−3^), differential absorption of left- and right-handed circularly polarized light by the chiral chromophore, quantified by *g*_abs_, manifests as a measurable difference in emission intensity, providing a luminescence-based readout of the excitation beam's circular polarization. Due to the sensitivity of these chromophores to environmental conditions, the correct combination of temperature, solvent, chromophore identity, and chromophore concentration must be known to decode a circularly polarized input (Fig. 1). More broadly, this effort illustrates how straightforwardly accessible chiral chromophores can be useful in applied chiroptics.

**Fig. 1:**
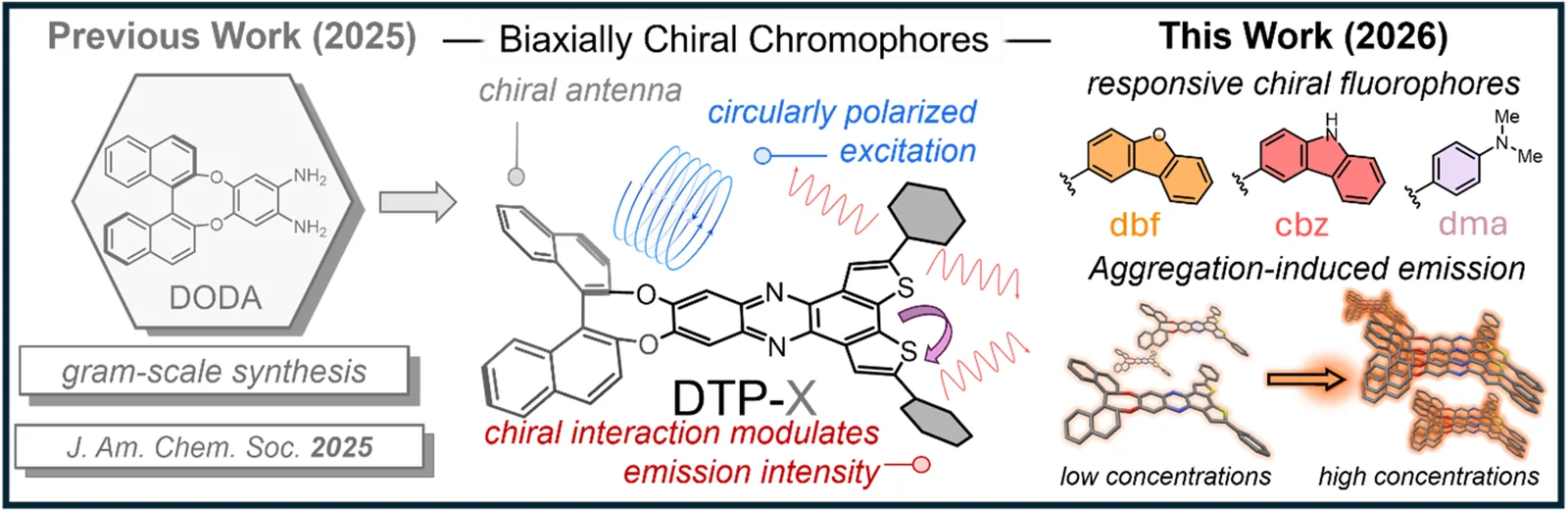
General concept of chiral chromophores used in this work, where a chiral absorbing unit reports a preferential circularly polarized absorption by an achiral and differential luminescence intensity.

### Chromophore synthesis

Our synthesis begins with the central brominated chromophore DTP-Br, accessed from *R*/*S*-[1,1′-binaphthalene]-2,2′-diol (BINOL) in three steps. Installing an arylenediamine functionality to the BINOL scaffold gives benzodinaphtho[1,4]dioxicine-2,3-diamine (DODA, see SI S2), which we condense with a brominated dione (Br_2_-BDTD) to form the central phenazine ring and access DTP-Br as a single enantiomer without the need for enantiomeric purification.^43^ This route is a direct extension of the biaxial contortion strategy we reported previously, in which chirality installed at the BINOL-derived DODA axis is transferred to a second, orthogonal axis defined by the newly formed phenazine. We have previously demonstrated that this conserved biaxially chiral precursor can be prepared in large quantities, which makes it well suited for subsequent library generation at a useful scale, a practical advantage over axially chiral helicene- or binaphthyl-based emitters that typically require chromatographic enantioseparation at every stage of derivatization.^37^

From DTP-Br, we prepared three dithienophenazine (DTP-X) chiral chromophores (X = -dbf, -cbz, -dma) through a single, divergent late-stage functionalization (Fig. 2). Palladium-catalyzed Suzuki–Miyaura cross-couplings of DTP-Br with 2-dibenzofuranylboronic acid, 2-carbazolylboronic acid (or ester), and 4-(dimethylamino)phenylboronic acid, respectively, gives DTP-dbf (98%), DTP-cbz (31%), and DTP-dma (27%) (SI S2). Because all three derivatives are installed onto the enantiopure DTP-Br intermediate *via* the same coupling chemistry, this divergent strategy decouples the chirality (set at the DODA stage) from electronic tuning (set at the final stage), letting us probe donor strength as a clean structural variable without re-deriving the chiral core for each substrate. We hypothesized that progressively increasing the donor strength of the DTP-X substituent, from the electron-poor bromide in DTP-Br to a strongly electron-donating dimethylaniline in DTP-dma, would intensify intramolecular charge transfer. In this manner, we could prove the energy gap law in the DTP-X series, where an enhanced charge-transfer character would come at the cost of emission intensity.

**Fig. 2:**
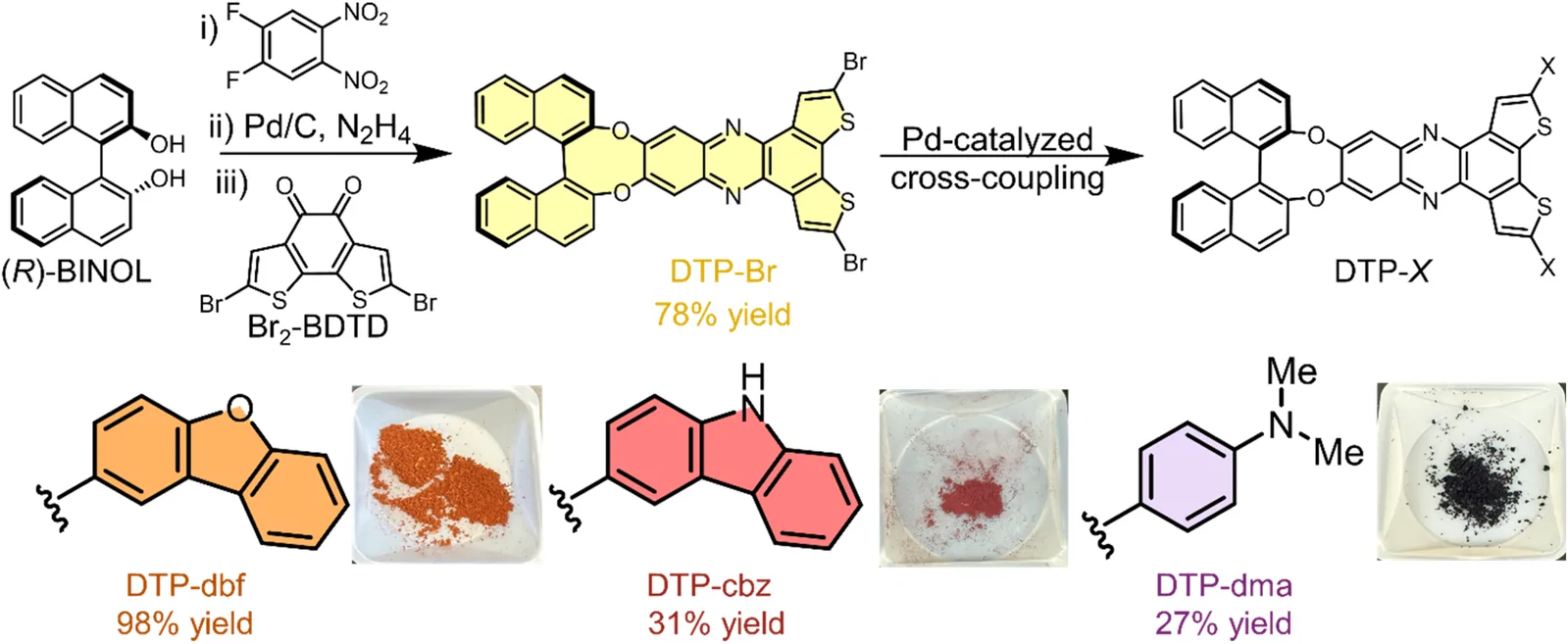
Synthesis of biaxially chiral dithienophenazine chromophores. Synthesis with (*R*) or (*S*) BINOL and photographs of the synthesized chromophores: (left to right) DTP-dibenzofuran (DTP-dbf), 98% yield with corresponding rustic orange powder; DTP-carbazole (DTP-cbz), 31% yield with corresponding crimson red powder; DTP-dimethylaniline (DTP-dma), 27% yield with corresponding plum purple powder. All (*S*) enantiomers were synthesized using the same procedure as the (*R*) enantiomers and have the same yield.

### Optical absorption spectroscopy

Increased electron donation from the pendent arene leads to more pronounced donor–acceptor character in chiral DTP-X chromophores. The high-energy (<450 nm) optical absorption profiles of these DTP-X chromophores originate from the biaxially chiral scaffold, as we have previously shown, with the high-energy band's molar absorptivity ranging from 41 000 M^−1^ cm^−1^ (DTP-Br) to 44 000 M^−1^ cm^−1^ (DTP-dma) across the series. We also observe that as the electron-donating character of the peripheral units increases, a more intense, broad, red-shifted absorption feature emerges, which we assign as a charge-transfer (CT) band. Specifically, DTP-Br does not display this red-shifted absorbance, consistent with the electron-withdrawing character of the bromide substituents. Conversely, DTP-dbf (530 nm onset, 7300 M^−1^ cm^−1^ at 476 nm), DTP-cbz (590 nm onset, 8700 M^−1^ cm^−1^ at 500 nm), and DTP-dma (610 nm onset, 4500 M^−1^ cm^−1^ at 527 nm) all exhibit this pronounced, broad CT feature. This suggests that pendant arene installation can be used to manipulate the electronics of the central DTP chromophore.

Experimental optical spectroscopy and time-dependent density functional theory (TD-DFT, at the cam-B3LYP/def2-TZVP level of theory) calculations collectively reveal that increasing the electron-donating character of the peripheral aryl units yields more pronounced D–A–D behavior across the DTP-X series (see SI Fig. S37). Experimentally, we find that DTP-dbf and DTP-Br are highly fluorescent, while DTP-cbz and DTP-dma are weakly emissive. The stark contrast in their emission profiles is fundamentally driven by these varying donor strengths and their impact on the excited-state energy gap. In DTP-dbf, the moderate electron-donating nature of the oxygen atom restricts the push–pull character, allowing the molecule to maintain a locally excited geometry with a S_1_ → S_0_ energy gap (2.93 eV) that favors radiative decay. In contrast, the stronger π-donating carbazole and tertiary amine units in DTP-cbz and DTP-dma drives the formation of a highly polarized, quinoid-like architecture. This electronic push–pull effect significantly narrows the adiabatic energy gap to 2.81 eV and 2.67 eV, respectively. TD-DFT calculations show good qualitative overlap between computation and experiment (Fig. S37), and reveal that the most red-shifted absorption band is dominated by HOMO → LUMO contributions (>90%), a transition that displays partial charge transfer character (Fig. 3e).^44–47^ These computational simulations reveal that installing arenes on the periphery of the DTP scaffold is a straightforward way to manipulate the electronic structure of the central chiral chromophore.

**Fig. 3:**
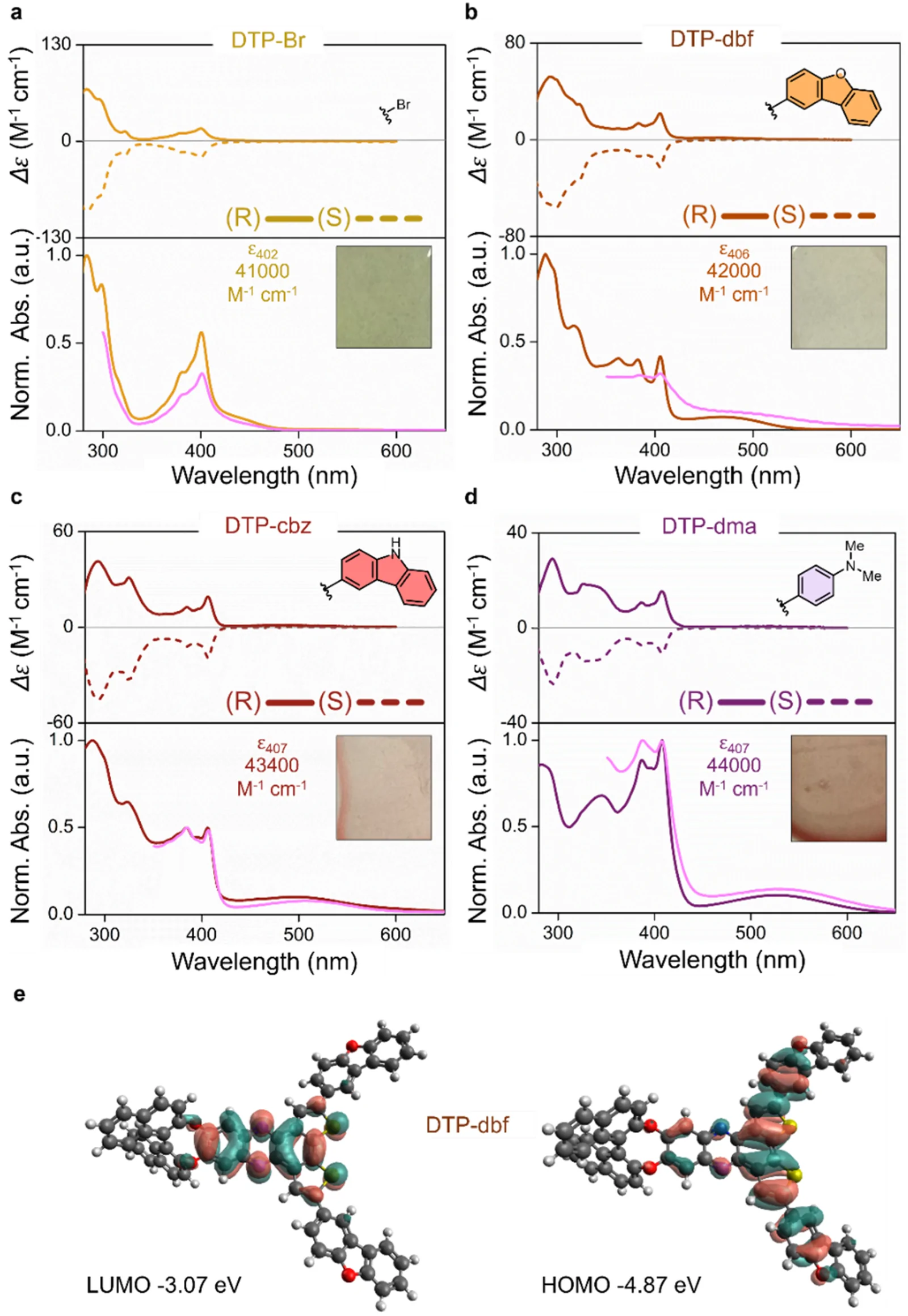
Circular dichroism and UV-visible spectroscopy of DTP-X chromophores. (a–d) Solution-state UV-vis spectra at 10 µM at 25 °C in CH_2_Cl_2_ (bottom) with PMMA film spectra overlaid (pink lines) and CD of DTP-X derivatives at 10^−6^ M at 25 °C in CH_2_Cl_2_ (top). Inserts: photographs of PMMA film of DTP-X derivatives. (e) LUMO (left) and HOMO (right) maps of DTP-dbf as determined by density functional theory calculations.

The circular dichroism (CD) spectra of all biaxial chromophores exhibit monosignate, broadband chiral absorption between 280 and 410 nm with Δ*ε* values between 20–60 M^−1^ cm^−1^ that do not cross zero. By exchanging the enantiopure *R*-DODA for its *S*-enantiomer as a starting material, we produce the opposite enantiomer DTP-X chromophores, which afford mirror-image CD signals (Fig. 3a–d dashed lines). Our synthetic route thus retains the chiroptical integrity after subsequent derivatization of the biaxial DTP-Br scaffold.^37^ This also validates that this platform is amenable to rapid chiroptical derivatization, which would be challenging in other systems that thermally racemize during functionalization.^48^ The intramolecular charge-transfer feature observed in the UV-vis spectra between 410 nm and 600 nm in DTP-dbf, DTP-cbz, and DTP-dma is absent in the corresponding CD spectra. The absence of this signal indicates that chiral information is not transferred over the biaryl cleft. This is likely due to the small torsional barrier of the phenyl-thiophene bond, which allows for rapid rotation under the conditions we investigate.^49–51^ Regardless, while the biaxial core of the strongly absorbing DTP unit exhibits chiroptical characteristics, the CD spectra at longer wavelengths are unaffected by the presence of electron-donating units at the periphery of this chromophore.

While solution-state investigation enables more straightforward analysis of optical characteristics, technological applications benefit from chiroptical materials that are deployable in solid-state architectures. We find that our chiral chromophores are readily incorporated into poly(methyl methacrylate) (PMMA) matrix *via* solution casting, which leads to colorful, transparent, and mechanically robust thin films (Fig. 3 inserts). We produce these thin films by first dissolving a small amount of the chiral chromophore (1–2 mg) into 5 mL of a PMMA CH_2_Cl_2_ solution (50 mg mL^−1^). We then solution-cast this mixture and allow it to air-dry, which provides uncomplicated access to homogeneous solid-state chiroptical films. As one example, DTP-dma is isolated as a dark purple powder. When we suspend this chromophore in a PMMA matrix and cast it, we obtain a deep purple DTP-dma film that is optically transparent (see SI S55). The optical absorption spectra and CD spectroscopy of these thin films are virtually identical to their solution-state measurements (Fig. 3a–d, see SI Fig. S21 and 22). The matrix-compatibility of these tunable chiral DTP-X chromophores underscore their potential relevance in chiroptical devices.

### Excited-state solvatochromism

We serendipitously noticed that the luminescence wavelength and intensity of DTP-X chromophores are strongly influenced by their solvent environment, which we attribute to the dipolar D–A–D character of DTP-X chromophores. At concentrations of 10 µM and at 23 °C in toluene (*ε* = 2.38), DTP-dbf is strongly luminescent with emission centered at 567 nm (Stokes shift of 98 nm) when excited at 365 nm, whereas DTP-Br demonstrates the opposite behavior (Fig. 4c) and is only weakly emissive in this solvent under these same conditions. These emission profiles are qualitatively retained when the chromophores are embedded in a PMMA matrix (*ε* = 3.0), consistent with their toluene-solvated behavior. In more polar solvents, both compounds red-shift, with DMSO-solvated (*ε* = 46.7) emission centered at 552 nm (Stokes shift of 150 nm) and 613 nm (Stokes shift of 133 nm) for DTP-Br and DTP-dbf, respectively. These red-shifted emission spectra are consistent with stabilization of the strongly dipolar excited-state. Despite these solvent shifts, the lack of a linear trend in the calculated Lippert–Mataga plot for DTP-Br and DTP-dbf suggests that the stabilizing solvent interactions are more complex than those that could be attributed to dielectric medium effects (see SI S38 and 39). Density functional theory geometry optimizations offer a partial structural explanation for this behavior in DTP-dbf: the relaxed excited-state (S_1_) geometry shows the dibenzofuran arms rotated toward coplanarity with the dithienophenazine core relative to the ground state (Fig. 4f), consistent with a quinoid-type excited state. However, DTP-Br experimentally displays similar solvatochromic emission-wavelength behavior despite lacking any peripheral aryl group capable of this rotation. Given that its solvatochromism trend runs opposite to DTP-dbf's, this indicates that coplanarization of the dibenzofuran arm is not a general mechanism for solvent-dependent quenching across the series, and that the wavelength shift and emission intensity may have at least partially distinct origins. Consistent with their more dipolar D–A–D ground-state structure and the presence of significant intramolecular charge-transfer, DTP-cbz and DTP-dma were found to be only weakly emissive across all conditions that we investigated (see SI S47–51). Similar to other phenazine-based chromophores, we observe that these materials are halochromic, with protonation leading to red-shifted absorption and circular dichroism, coupled with a suppression of emission intensity (Fig. S46).^52^ These optical characteristics highlight another axis of responsivity in these biaxially chiral chromophores.

**Fig. 4:**
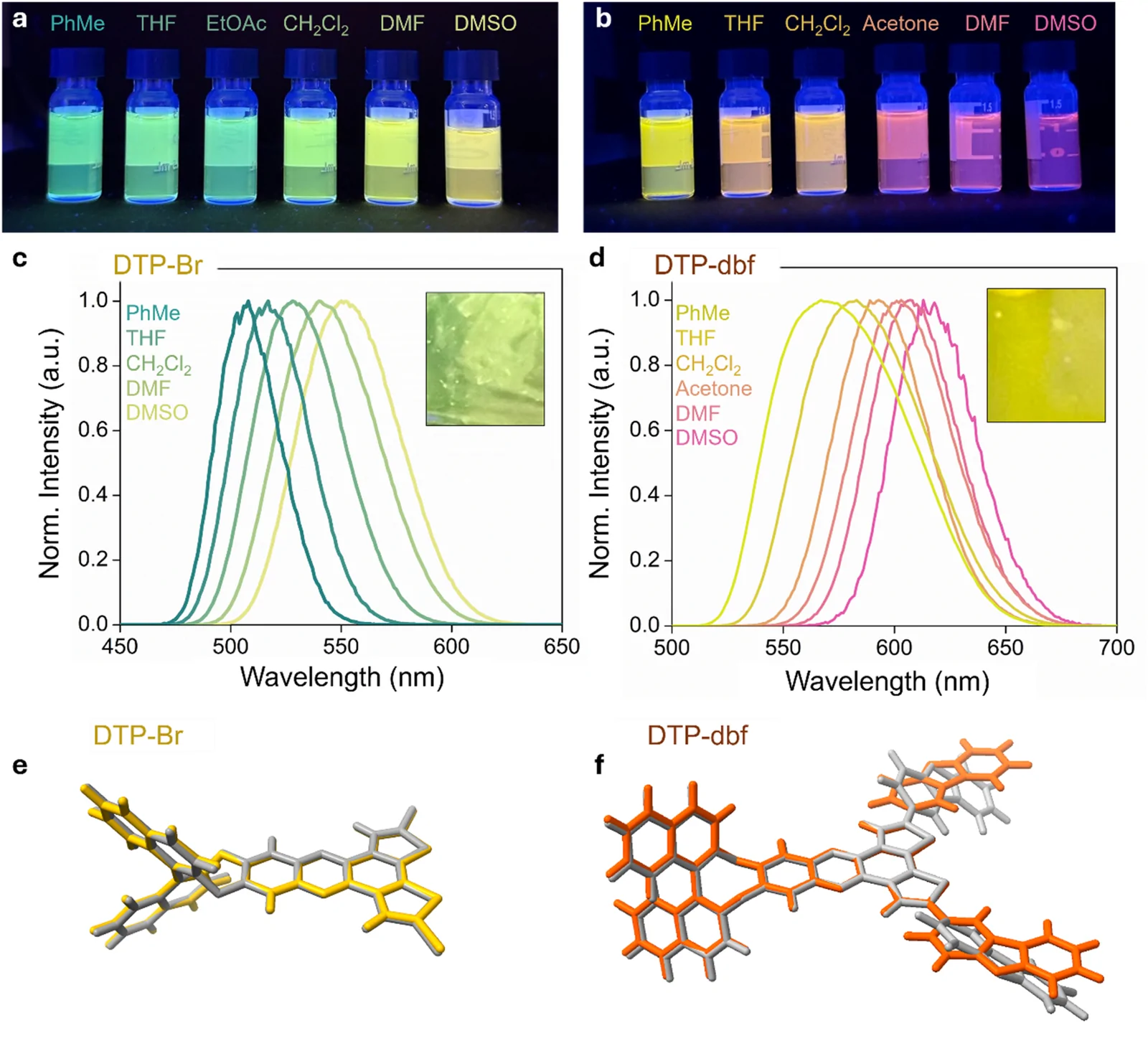
Solvent-responsive emission of DTP-Br and DTP-dbf. (a and b) Photoluminescence image of DTP-Br (left, green to yellow) and DTP-dbf (right, yellow to red) in different solvents (labeled in color above vial). The concentration of vial samples was increased to better represent the solvatochromism. (c and d) Solvent-variable emission of DTP-Br and DTP-dbf. Inserts: photographs of PMMA film of DTP-Br and DTP-dbf under 365 nm irradiation. (e and f) Relaxed geometries of ground-state S_0_ (grey) and excited-state S_1_ (yellow, DTP-Br, and orange, DTP-dbf) of *R*-DTP-Br and *S*-DTP-dbf.

DTP-dbf exhibits pronounced aggregation-induced emission at high concentrations, as evidenced by an increase in PLQY with increasing concentration in toluene.^53,54^ Specifically, we find that the PLQY increases from <1% at 2 µM in toluene to >50% at 18 µM (Fig. 5). Over this same concentration range, UV-vis absorbance intensity increases linearly without discernible shifts in *λ*_abs_ (Fig. 5c, insert), which reveals that the ground-state electronics are not significantly impacted by aggregation. Together, these results establish that emission intensity in this system can be tuned independently through at least three parameters: solvent polarity, concentration, and temperature (see SI Fig. S42 and S45). The sensitivity to environmental conditions led us to speculate that these conditions could act as a key to recording and decoding the circular polarization of light. Moreover, the enhanced fluorescence intensity of DTP-dbf that emerges from aggregation-induced emission led us to speculate that we could exploit these higher emission intensities in a fluorescence-detected circular dichroism configuration.

**Fig. 5:**
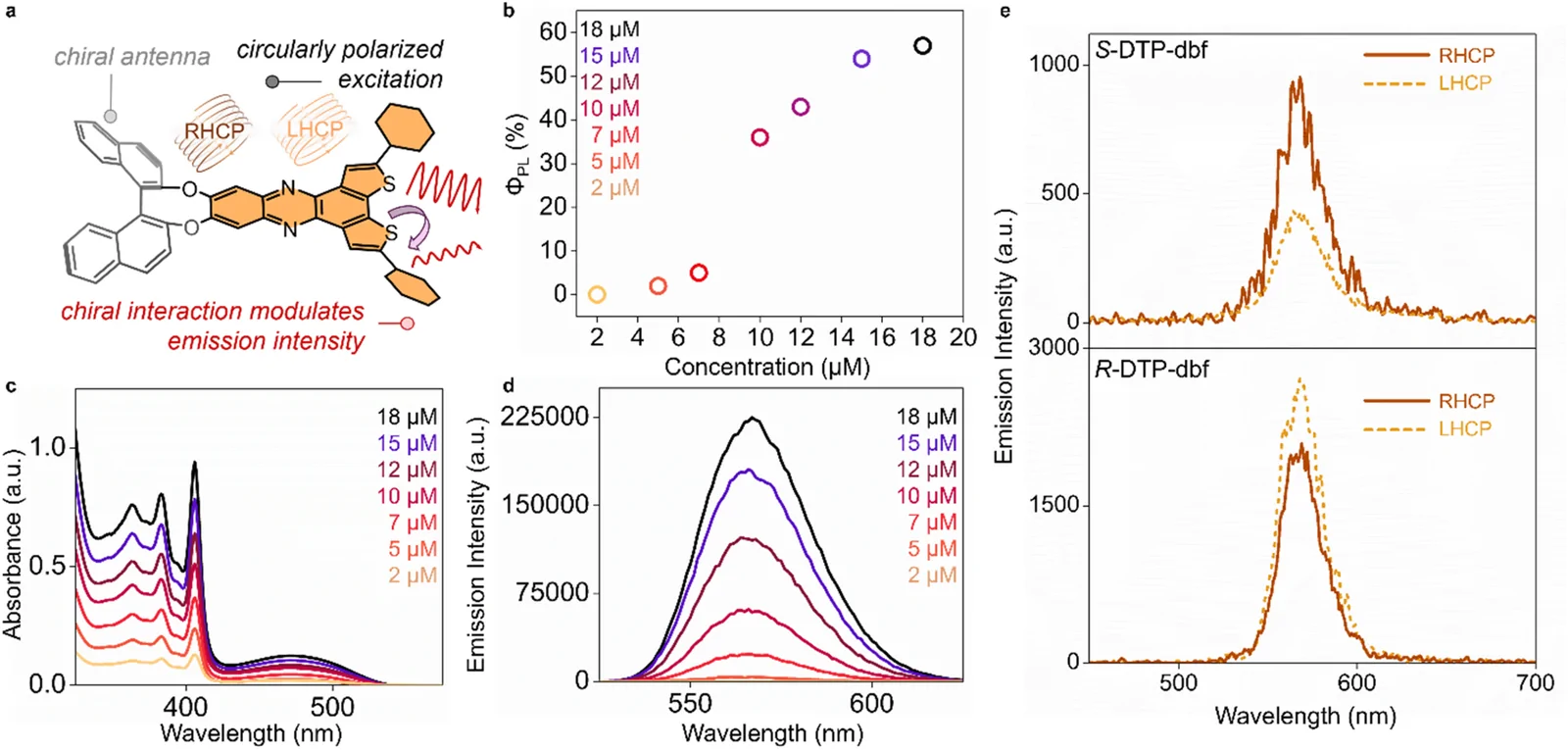
Differential emission of enantiopure DTP-dbf with dichroic filters. (a) Cartoon diagram of differential emission intensity as a function of incoming circularly polarized light; (b) plot of quantum yield *versus* sample concentration in toluene; (c) UV-vis spectra of the same samples used to determine PLQY; (d) photoluminescence spectra of same samples used to determine PLQY; (e) PL of different emission intensities with right-handed (RHCP) and left-handed (LHCP) circularly polarized light and *S*- or *R*-DTP-dbf at similar concentrations.

### Quantum encryption with biaxially chiral fluorescence reporters

Circularly polarized light is most often detected by passing the incident beam through a linear polarizer and a quarter-wave plate or photoelastic modulator and finally analyzing the differential intensity with a photodetector. This approach has several drawbacks. First, its mechanical components pose inherent challenges for miniaturizing and rapidly operating this sensor. Second, the linear polarizer reduces the measured intensity by at least 50%. Third, this approach contains no way to layer additional security into the quantum state of the light. Directly detecting the circular polarization through a material's intrinsic optical response would address these challenges. A chromophore with a tunable and high PLQY can help overcome these drawbacks and enable applied investigations in areas such as anti-counterfeiting or optical sensors. Critically, leveraging a dynamic chromophore in these applied settings only works if the reader knows how to match the chromophore's environment (solvent, concentration, temperature) to the conditions under which it was encoded. Thus, a security feature could be designed so that the correct emission intensity, and therefore the encoded information, is only recovered when the circular polarization of the incoming light is read under the correct combination of conditions.

Because the chiral DTP core and the emissive periphery are electronically decoupled, these chromophores absorb left- and right-handed circularly polarized light differentially but emit unpolarized light, meaning any polarization-dependent change in emission intensity must originate from differential absorption (*g*_abs_) rather than differential emission (*g*_lum_). Using the biaxial chromophores reported above, we exploit this distinction to directly detect the circular polarization of light without additional optical components (Fig. 5b). Based on the difference in molar absorption (*e.g.*, Δ*ε*) associated with left or right-handed circularly polarized light (LHCP or RHCP) in these biaxially chiral scaffolds, the emission intensity also varies depending on the chirality of the incoming light. For example, exciting DTP-dbf (0.5 mM in PhMe) with 440 nm light that is either left- or right-handed circularly polarized yields nearly identical spectra. The maximum emission intensity (558 nm) is reduced when right-handed circularly polarized light is used to excite *R*-DTP-dbf compared to the emission intensity when left-handed circularly polarized light is used. Importantly, this difference in emission intensity is inverted when the opposite chirality of *S*-DTP-dbf is used to perform this same measurement (see Fig. 5e). We note the small difference in emission intensity for the *S*-DTP-dbf*versus* the *R*-DTP-dbf measurement. We attribute this to differences in sample concentration between the two measurements and small variabilities in filter efficiency between the two circular polarization filters. As an achiral control, Rhodamine 6G was also measured with the dichroic filters and we observed no difference in emission intensity (see SI Fig. S54). The precise color and magnitude of the emission can be encoded using solvent, temperature, and enantiomer of the chromophore. This means that without understanding these features the circular polarization of the incoming light could not be reliably decoded. This finding hints at the exciting possibility that if the wavelength of the incoming light and the emissive chromophore conditions (*e.g.*, identity, concentration, temperature, solvent environment, protonation state, emission wavelength) can be coordinated, then the polarization of light could be measured by simply recording the chromophore's differential emission intensity. We envision that this direct, all-optical detection of circular polarization could provide a route to rapidly verify information encoded in circularly polarized light, for example, in anti-counterfeiting or optical-tagging applications.

## Conclusion

In this work, we designed a series of optically tunable, responsive, and chiral dithienophenazine chromophores. The synthetic ease of access to these materials enabled our optical study and a subsequent investigation into an applied chiroptical detection scheme. Previous work in this field has successfully tuned chiroptical properties through structural design but has often done so at the expense of other desirable optical characteristics. The synthetic strategy we report here provides a divergent platform for demonstrating how donor–acceptor–donor features manifest in chiral chromophores. With these DTP-X chromophores, we can access broad-range linear (250–700 nm) and chiral (280–480 nm) absorption. Additionally, we can tune the emissive properties of these chromophores by changing the solvent, temperature, and enantiomeric identity. The finding that emission intensity is sensitive to the circular polarization of the excitation led us to speculate that these molecules could be used to measure circular polarization by simply measuring luminescence intensity. This led us to exploit the environmental sensitivity of this system to decode the circular polarization of light used to excite these molecules. The ability to compatibilize these chromophores in a solid-state PMMA matrix without compromising optical integrity further demonstrates their potential utility in chiroptical applications. More broadly, we expect that non-planar molecular designs may also provide co-functionality associated with ion transport or thermomechanical responses,^55–61^ which may open additional avenues of exploration for solution-processable, scalable chiral chromophores. We anticipate that the tunability, ease of synthesis and processability, and sensitive optical features of this system are desirable characteristics for future chiroptical structure–property relationship investigations.

## Author contributions

A. M. E. and C. P. S. conceptualized, supervised, and administered this work. S. J. B. collected the experimental data, curated the data, and performed analysis on this data. J. Z., D. K. S., and B. S. D. performed experiments as part of this investigation. All authors were involved in the writing and revision of this manuscript.

## Conflicts of interest

There are no conflicts to declare.

## Supplementary Material

SC-017-D6SC05555A-s001

## Data Availability

The data that support the findings of this study are available within the article and its supplementary information (SI) files. Experimental protocols, compound characterization data (^1^H NMR, high-resolution mass spectrometry, and FTIR spectra), and electroanalytical measurements (cyclic voltammetry) are provided in the SI. The optimized molecular geometries, total energies, and time-dependent density functional theory (TD-DFT) computational data are detailed in the SI. Correspondence and requests for additional optical spectroscopy datasets or raw computational files should be addressed to the corresponding author. Supplementary information is available. See DOI: https://doi.org/10.1039/d6sc05555a.
