# Supplementary material for: Decoding circular polarization with biaxially chiral fluorescence reporters
Source: Chem Sci. 2026 Jul 31;17(37):17916–25. doi: 10.1039/d6sc05555a (PMC13446138; doi:10.1039/d6sc05555a)
Supplement: Supplementary file 1 [file SC-017-D6SC05555A-s001.pdf]

Supplementary Information (SI) for Chemical Science.
This journal is © The Royal Society of Chemistry 2026

Decoding Circular Polarization with Biaxially Chiral Fluorescence Reporters

Summer J. Brown[1], Jiaoyan Zhao[2,4], Delia K. Savin[1], Bella S. Davey[1], Cedric P. Schaack[2,4*], Austin M. Evans[1,3*]

[1]Department of Chemistry, University of Florida, Gainesville, Florida 32611, United States
[2]Department of Chemistry, Wake Forest University, Winston-Salem, North Carolina 27109, United States
[3]Department of Materials Science and Engineering, University of Florida, Gainesville, Florida 32611, United States
[4]Center for Functional Materials, Wake Forest University, Winston-Salem, North Carolina 27109, United States

Supporting Information

Professor Austin M. Evans
Department of Chemistry, University of Florida
Leigh Hall 132, 1680 Union Road, Gainesville, Florida, 32611
AustinEvans@ufl.edu

Professor Cedric P. Schaack
Department of Chemistry, Wake Forst University
Salem Hall 016A, 1834 Wake Forest Road, Winston-Salem, North Carolina, 27109
schaacc@wfu.edu

Table of Contents

S1 – Materials and Instrumentation 3

Nuclear Magnetic Resonance (NMR) Spectroscopy Information 3

UV-Visible Spectroscopy (UV-Vis) 3

Photoluminescence Spectroscopy 3

Electronic Circular Dichroism Spectroscopy (ECD) 3

Cyclic Voltammetry (CV) 3

High-Resolution Mass Spectrometry (HRMS) 3

Fourier-Transform Infrared Spectroscopy (FTIR) 3

S2 – Methods 4

Synthesis of Compounds 4

Benzo[b]dinaptho[2,1-e:1',2'-g][1,4]dioxicine-2,3-diamine (**DODA**) 4

2,7-dibromobenzo[1,2-b:6,5-b']dithiophene-4,5-dione (**Br_2-BDTD**) 4

17,20-dibromodinaphtho[2',1':5,6;1",2":7,8][1,4]dioxocino[2,3-*i*]dithieno[3,2-*a*:2',3'-*c*]phenazine (**DTP-Br**) 5

17,20-bis(dibenzo[b,d]furan-2-yl)dinaphtho[2',1':5,6;1",2":7,8][1,4]dioxocino[2,3-*i*]dithieno[3,2-*a*:2',3'-*c*]phenazine (**DTP-dbf**) 6

4,4'-(dinaphtho[2',1':5,6;1",2":7,8][1,4]dioxocino[2,3-*i*]dithieno[3,2-*a*:2',3'-*c*]phenazine-17,20-diyl)bis(N,N-dimethylaniline) (**DTP-cbz**) 7

17,20-di(9H-carbazol-3-yl)dinaphtho[2',1':5,6;1",2":7,8][1,4]dioxocino[2,3-*i*]dithieno[3,2-*a*:2',3'-*c*]phenazine (**DTP-dma**) 8

S3 - Nuclear Magnetic Resonance 9

S4 – Electroanalytical Characterization 14

S5 – Electronic Circular Dichroism 16

S6 – Computational Details 21

S7 – Ultraviolet-visible Spectroscopy 25

S8 – Photoluminescence Spectroscopy 30

S9 – Film Images 40

References 41

S1 – Materials and Instrumentation

Nuclear Magnetic Resonance (NMR) Spectroscopy Information
All ^{1}H spectra were recorded on a Bruker 400 MHz spectrometer at 25 °C. Chemical shifts were calibrated using residual solvent as an internal reference ($CDCl_3$: 7.26 ppm for ^{1}H NMR; DMSO-d_6: 2.50 ppm for ^{1}H NMR).

UV-Visible Spectroscopy (UV-Vis)
All UV-Vis spectroscopy was collected at 25 ℃ using a Cary 5000 UV-Vis-NIR instrument from 250 – 800 nm with a resolution of 0.1 nm. All spectra shown are with a solvent baseline correction. Unless otherwise specified, all samples were measured at a concentration of 10 μM.

Photoluminescence Spectroscopy
All photoluminescence measurements were taken at 25 ˚C using a HoribaJobin Yvon FluoroMax-4 spectrofluorometer. Shutter slit widths were 25 nm for excitation and emission unless otherwise stated. An integration time of 0.1s was used unless otherwise specified.

Electronic Circular Dichroism Spectroscopy (ECD)
All ECD spectra were recorded using a Jasco J-1500 spectrometer at 25 ˚C and recorded between 200–600 nm. Solutions (c ~ 10^{-6} M) were measured in quartz cuvettes with a pathlength of 1 cm. All spectra were recorded using a scanning speed of 100 nm min^{-1}, a step size of 1 nm, a bandwidth of 4 nm, a response time of 1 s, and averaged over 3 accumulations. The spectra were corrected using the spectrum of CH_2Cl_2 recorded under the same conditions immediately before or after the sample.

Cyclic Voltammetry (CV)
Cyclic voltammetry was carried out on a Bio-Logic SP-50e or SP-150e potentiostat with a three-electrode setup, using a glassy carbon electrode as a working electrode, an Ag wire as a pseudoreference electrode, and a Pt wire as a counter electrode. Samples were dissolved in CH_2Cl_2 at an average concentration of 5 mg/ml and then 500 μL was transferred to an MeCN electrolyte solution with 0.1 M $n\mathrm{Bu_4BF_4}$ as supporting electrolyte. Initial scans were carried out at 50 mV/s followed by additional scans with 5 μg ferrocene added as a reference. CVs were manually referenced using the referenced ferrocene data. Measurements were carried out at 25 ˚C.

High-Resolution Mass Spectrometry (HRMS)
Mass spectrometry data were obtained at the University of Florida mass spectrometry center using Agilent 6230 ESI-TOF mass spectrometer, a Bruker autoflex maX MALDI Time-of-Flight instrument in reflectron mode, or direct analysis in real time (DART) analyzed in positive mode.

Fourier-Transform Infrared Spectroscopy (FTIR)
Fourier transform infrared spectroscopy was performed using a Cary 630 instrument with a diamond attenuated total reflection (ATR) attachment with 1 cm^{-1} resolution in ambient atmosphere. Parameters are set to 120 scans. All samples were neat. All spectra are background corrected.

S2 – Methods

Synthesis of Compounds

All syntheses were carried out under an atmosphere of N_2 unless otherwise specified.
All chiral derivatives (both enantiomers) were synthesized using the same procedure albeit with enantiopure (*R*/*S*) starting material.

Benzo[b]dinaptho[2,1-e:1',2'-g][1,4]dioxicine-2,3-diamine (DODA)

Synthesis of DODA was repeated using a previously reported protocol.[1]

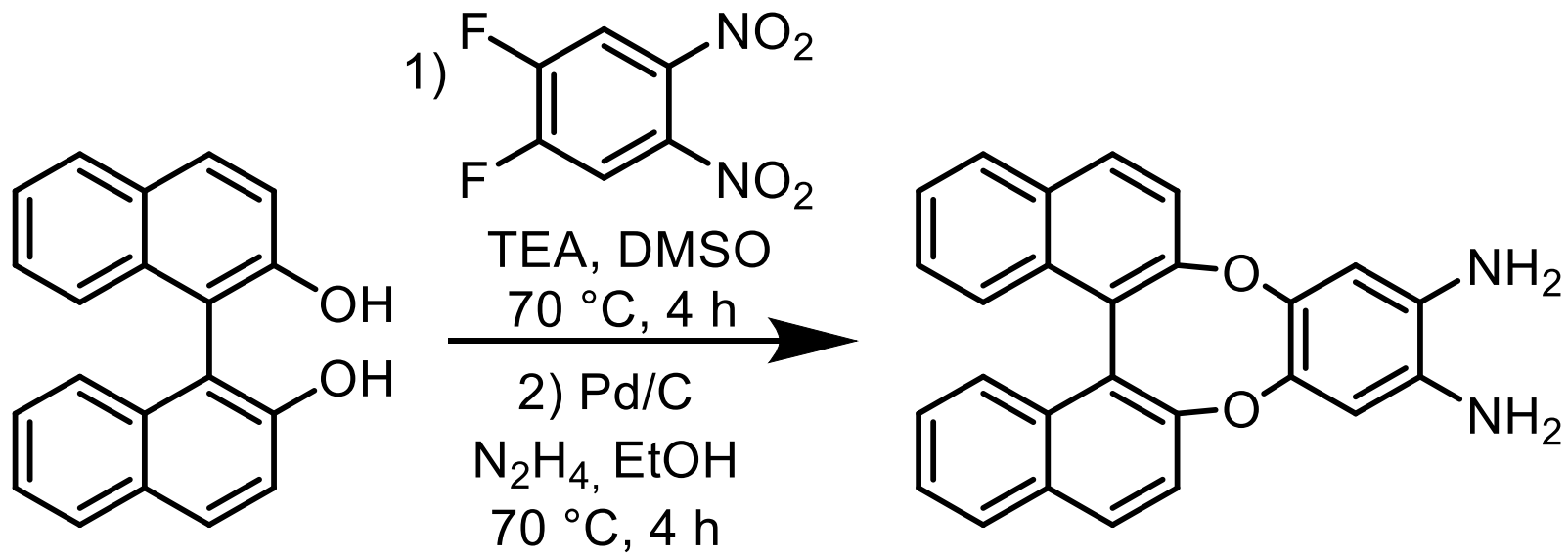

A 500 mL round bottom flask equipped with a stir-bar was charged with 1,1'-bi-2,2'-napthol (3 g, 10.5 mmol, 1 equiv) and 1,2-difluoro-4,5-dinitrobenzene (2.14 g, 10.48 mmol, 2.1 equiv). Anhydrous DMSO (40 mL) and triethylamine (3.1 mL, 22 mmol, 2.1 equiv) were then added to the flask. After stirring for 4 hours at 70 ℃ under a nitrogen atmosphere, the solution had turned from a light yellow to a deep red color. The reaction was allowed to cool to room temperature before being poured onto 200 mL of 1 M hydrochloric acid. A canary yellow solid crashed out of solution. Residual water and solvent were removed with a high vacuum (4.7 g, >99% yield). The product was used without further purification.

To a 500 mL round bottom flask equipped with a stir bar, product from step 1 was added (1.9 g, 4.2 mmol, 1 equiv) with 50 mg of Pd/C. Ethanol (20 mL) was then added to the flask. Subsequently, hydrazine monohydrate (2.8 mL, 58.3 mmol, 14 equiv) was added dropwise to the flask. After stirring for 4 hours at 70 ℃ under a nitrogen atmosphere, the reaction was allowed to cool to room temperature. A Celite© plug was used to remove the Pd/C and boiling ethanol was filtered through the plug to remove residual product. Solvent was then concentrated *in vacuo* until a brown slurry appeared. To the brown slurry was added DI water after which an off-white solid crashed out. The solid was filtered, and residual water and solvent were removed with a high vacuum (1.5 g, 90% yield).
^{1}H NMR matched previous reports.

2,7-dibromobenzo[1,2-b:6,5-b']dithiophene-4,5-dione (Br_2-BDTD)

Br_2- BDTD was synthesized following a method previously reported by Reynolds and coworkers.[2]

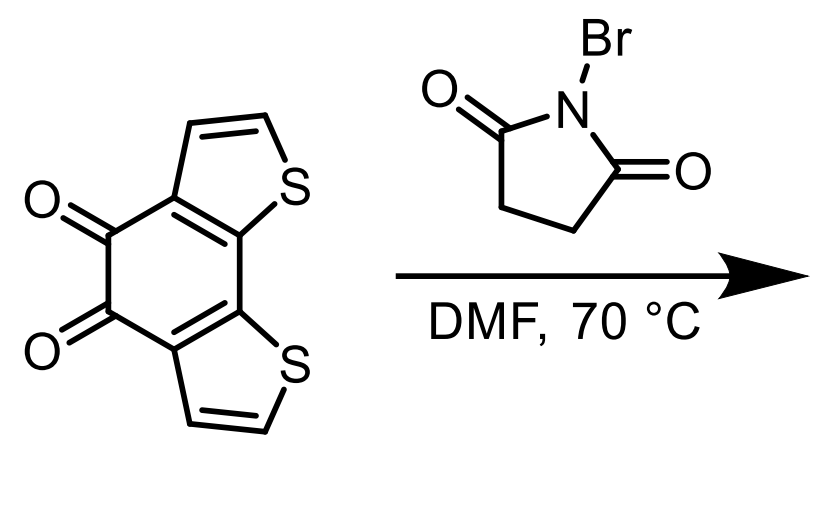

A 50 mL flask equipped with a stir bar was charged with benzo[1,2-b:6,5-b’]dithiophene-4,5-dione (300 mg, 1.36 mmol, 1 equiv), N-bromosuccinimide (509 mg, 2.86 mmol, 2.1 equiv), and DMF (8.2 mL). The mixture was set to stir at 70 ℃ for 18 hours. The solution was concentrated to 5 mL via rotary evaporation, and the product was subsequently precipitated by pouring the DMF into 30 mL of cold water. The precipitate was gravity filtered and air dried overnight (~12 hours) before being recrystallized with acetonitrile. The recrystallized product was a dark purple solid (359 mg, 70% yield).

^{1}H NMR matched previous reports.

17,20-dibromodinaphtho[2',1':5,6;1'',2'':7,8][1,4]dioxocino[2,3-*i*]dithieno[3,2-*a*:2',3'-*c*]phenazine (DTP-Br)

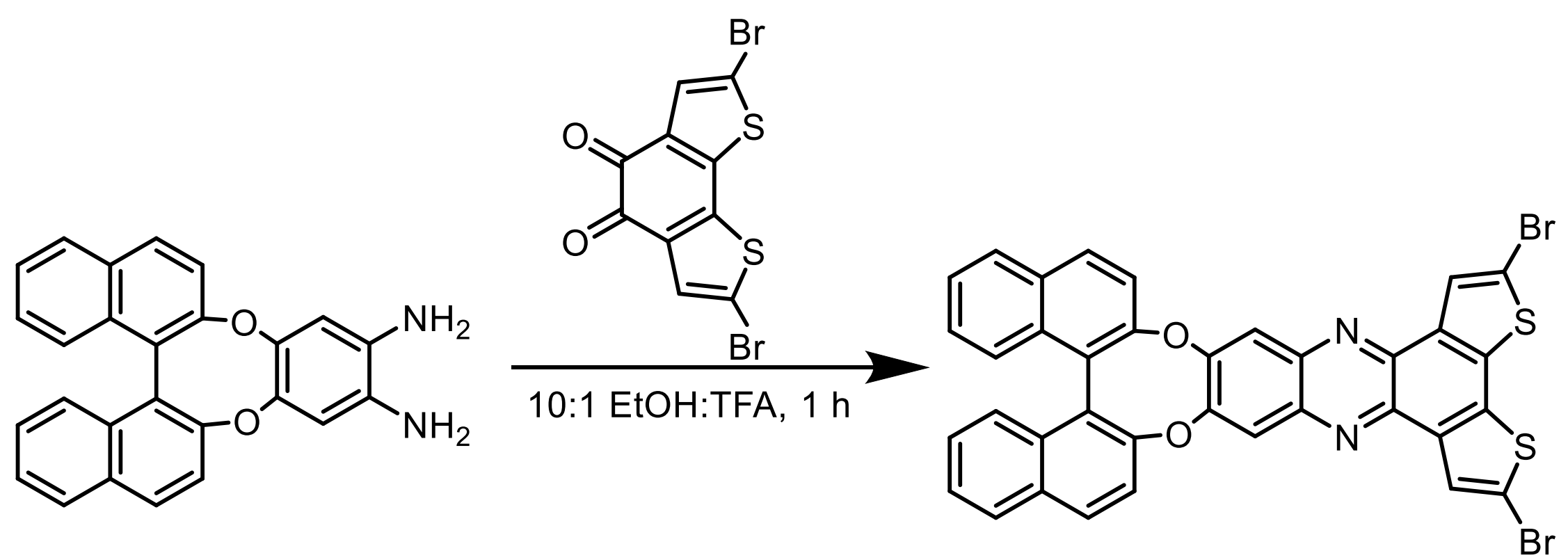

A 20 mL vial with a stir bar was charged with 24 mg of **DODA** (61.4 µmol, 1. equiv). Subsequently, 23 mg of **Br$_2$-BDTD** (61.4 µmol, 1 equiv) was added to the vial. The solids were dissolved in ethanol (1 mL) and trifluoroacetic acid (0.1 mL). The resulting mixture was stirred at 70 °C. After stirring for 1 hour, the reaction mixture was transferred to a beaker with 20 mL 1 M K_2CO_3 and stirred for 5 minutes. The dark yellow solid was then gravity filtered and dried with a high-vacuum (35 mg, 48 µmol, 78% yield).

^{1}H NMR (400 MHz, DMSO-*d$_6$*) δ 8.44 (s, 2H), 8.31 (s, 2H), 8.23 (d, *J*= 8.9 Hz, 2H), 8.14 (d, *J*=8.0 Hz, 2H), 7.85 (d, *J*=8.9 Hz, 2H), 7.61 (t, *J*=7.5 Hz, 2H), 7.53 (t, *J*=7.6 Hz, 2H), 7.47 ppm (d, *J*=8.3 Hz, 2H).

^{13}C NMR not measured due to limited solubility.

IR (neat): 758, 948, 1200, 1235, 1330, 1345, 1405, 1457, 1587, 2922, 3050.

HRMS (ESI–TOF, $[M+H]^+$) *m/z* calcd. for $C_{36}H_{16}{}^{79}Br_2N_2O_2S_2$ 730.9093; Found 730.9087. calcd. for $C_{36}H_{16}{}^{79}Br^{81}BrN_2O_2S_2$ 732.9000; Found 732.9075. calcd. for $C_{36}H_{16}{}^{81}Br_2N_2O_2S_2$ 734.8979; Found 734.9063

17,20-bis(dibenzo[b,d]furan-2-yl)dinaphtho[2',1':5,6;1'',2'':7,8][1,4]dioxocino[2,3-*i*]dithieno[3,2-*a*:2',3'-*c*]phenazine (DTP-dbf)

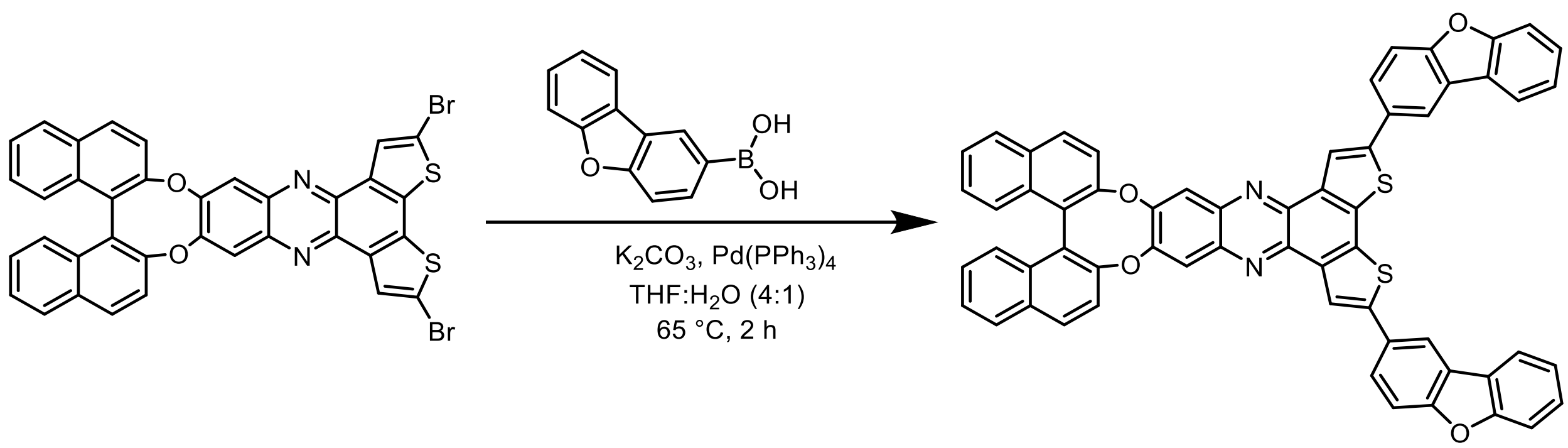

A 100 mL round bottom flask equipped with a stir bar was charged with 85 mg of **DTP-Br** (116 µmol, 1.0 equiv.). Subsequently, 51 mg of dibenzo[b,d]furan-2-ylboronic acid (243.7 µmol, 2.1 equiv.) was added to the flask followed by 96 mg K_2CO_3 (696.28 µmol, 6 equiv.) and 13 mg $Pd(PPh_3)_4$ (11.6 µmol, 0.1 equiv.). The solids were dissolved in a 4:1 mixture of THF: H_2O (8 mL: 2 mL). The resulting mixture was stirred at 65 °C. After stirring for 2 hours, the reaction mixture was transferred to a separatory funnel and extracted with CH_2Cl_2 (10 mL ×3). The combined DCM extracts were washed with brine, dried over Na_2SO_4, filtered, and concentrated *in vacuo* to yield **DTP-dbf** as a rustic orange powder (104 mg, 114 µmol, 98% yield).

^{1}H NMR (400 MHz, DMSO-*d*$_6$) δ 8.86 (s, 2H), 8.78 (s, 2H), 8.41 – 8.29 (m, 4H), 8.26 (d, *J* = 9.0 Hz, 2H), 8.16 (d, *J* = 8.2 Hz, 2H), 8.06 (d, *J* = 8.9 Hz, 2H), 7.88 (d, 2H), 7.75 (dd, 4H), 7.59 (dq, *J* = 21.1, 7.1 Hz, 6H), 7.47 ppm (t, *J* = 8.7 Hz, 4H).

^{13}C NMR not measured due to limited solubility.

IR (neat): 745, 1121, 1226, 1326, 1349, 1462, 2355, 3045.

HRMS (DART–TOF, $[M+H]^+$) *m/z* calcd. for $C_{60}H_{30}N_2O_4S_2$ 907.1720; Found 907.1734.

17,20-di(9H-carbazol-3-yl)dinaphtho[2',1':5,6;1'',2'':7,8][1,4]dioxocino[2,3-*i*]dithieno[3,2-*a*:2',3'-*c*]phenazine (DTP-cbz)

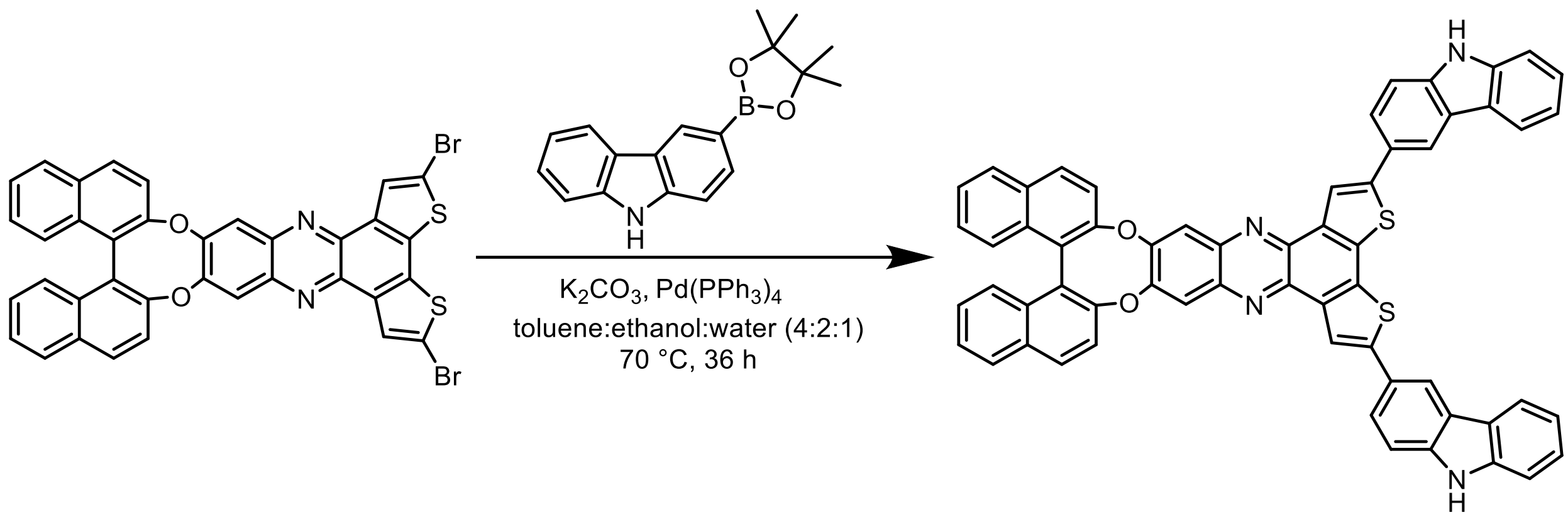

A 100 mL round bottom equipped flask with a stir bar was charged with 38 mg of **DTP-Br** (52.43 µmol, 1.0 equiv.). Subsequently, 33 mg of 3-(4,4,5,5-tetramethyl-1,3,2-dioxaborolan-2-yl)carbazole (112.7 µmol, 2.15 equiv.) was added to the flask followed by 43 mg K_2CO_3 (314.6 µmol, 6 equiv.) and 6.1 mg $Pd(PPh_3)_4$ (5.2 µmol, 0.1 equiv.). The solids were dissolved in a 4:2:1.25 mixture of toluene: ethanol: water (4 mL: 2 mL: 1.25 mL). The resulting mixture was stirred at 70 °C. After stirring for 36 hours, the reaction mixture was transferred to a separatory funnel and extracted with CH_2Cl_2 (10 mL x 3). The combined DCM extracts were washed with brine, dried over Na_2SO_4, filtered, and concentrated *in vacuo*. The crude material was purified by column chromatography (100 mL glass chromatography column, 30 mm O.D., 5.25 inches tall of SiliaFlash silica gel (40-63 µm), slurry loaded in hexanes using SiliaFlash silica gel (40-63 µm) eluting 50 → 90% hexanes to CH_2Cl_2 (10% increments, 100 mL each) followed by 100% CH_2Cl_2 until all product was eluted to yield **DTP-cbz** as a dark crimson red powder (15 mg, 16.57 µmol, 31% yield).

^{1}H NMR (400 MHz, DMSO-*d₆*) δ 11.52 (s, 2H), 8.85 (s, 2H), 8.77 (s, 2H), 8.38 (d, J = 9.7 Hz, 4H), 8.25 (d, J = 9.0 Hz, 2H), 8.16 (d, J = 8.1 Hz, 2H), 8.01 (d, J = 10.0 Hz, 2H), 7.89 (d, J = 8.8 Hz, 2H), 7.63 (dd, J = 12.5, 7.9 Hz, 4H), 7.59 – 7.52 (m, 4H), 7.47 (dd, J = 18.9, 8.3 Hz, 4H), 7.25 ppm (t, J = 7.4 Hz, 2H).

^{13}C NMR not measured due to limited solubility.

IR (neat): 728, 745, 950, 1203, 1226, 1408, 1461, 1582, 2927, 3060.

HRMS (DART–TOF, M^+) *m/z* calcd. for $C_{60}H_{32}N_4O_2S_2$ 904.1966; Found 904.1933.

4,4'-(dinaphtho[2',1':5,6;1'',2'':7,8][1,4]dioxocino[2,3-*i*]dithieno[3,2-*a*:2',3'-*c*]phenazine-17,20-diyl)bis(N,N-dimethylaniline) (DTP-dma)

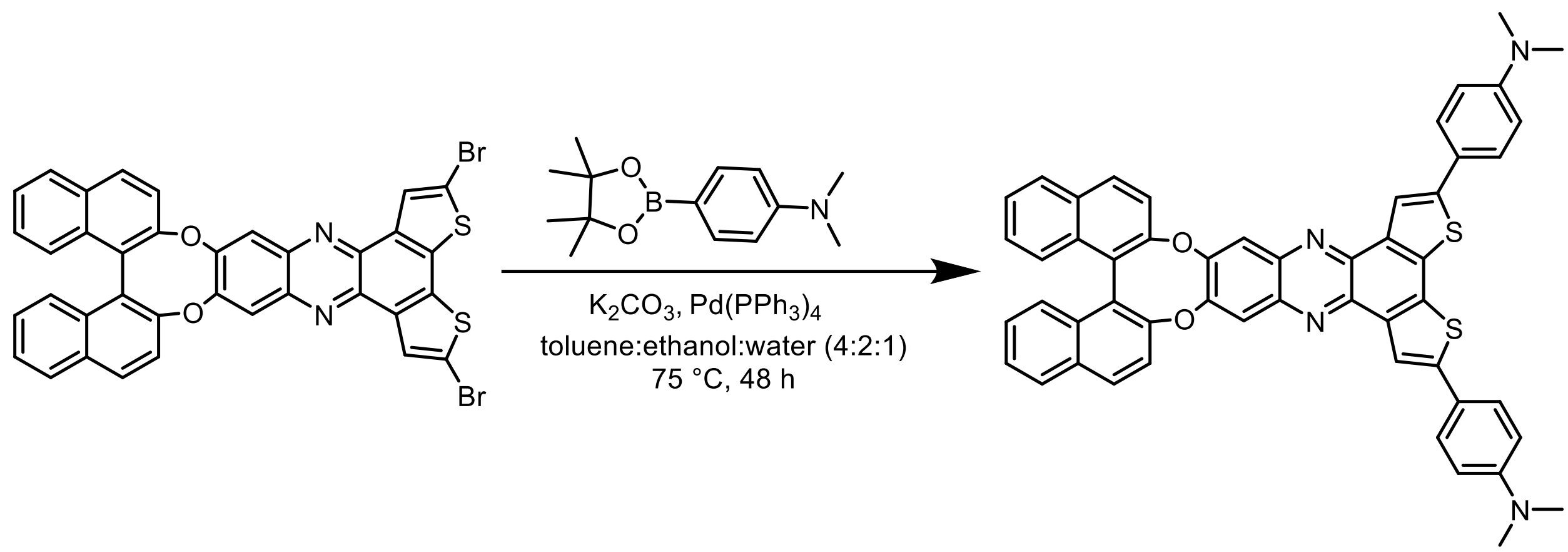

A 100 mL round bottom flask equipped with a stir bar was charged with 51 mg of **DTP-Br** (69.63 µmol, 1.00 equiv). Subsequently, 41.3 mg of *N*,*N*-dimethyl-4-(4,4,5,5-tetramethyl-1,3,2-dioxaborolan-2-yl)-aniline (167.1 µmol, 2.40 equiv) was added to the flask followed by 57.7 mg K_2CO_3 (417.7 µmol, 6.0 equiv) and 8.1 mg $Pd(PPh_3)_4$ (6.9 µmol, 0.10 equiv). The solids were dissolved in a 4:2:1 mixture of toluene: ethanol: water (5 mL: 2.5 mL: 1.25 mL). The resulting mixture was stirred at 75 ℃. After stirring for 48 hours, the reaction mixture was transferred to a separatory funnel and extracted with CH_2Cl_2 (10 mL x 3). The combined DCM extracts were washed with brine, dried over Na_2SO_4, filtered, and concentrated *in vacuo* to yield **DTP-dma** as a plum purple powder (15 mg, 18.45 µmol, 27% yield).

^{1}H NMR (400 MHz, DMSO-*d₆*) δ 8.42 (s, 2H), 8.32 (s, 2H), 8.24 (d, *J* = 8.9 Hz, 2H), 8.15 (d, *J* = 8.2 Hz, 2H), 7.87 (d, *J* = 8.9 Hz, 2H), 7.73 (d, *J* = 8.6 Hz, 4H), 7.62 (t, *J* = 7.4 Hz, 2H), 7.54 (t, *J* = 7.6 Hz, 2H), 7.48 (d, *J* = 8.4 Hz, 2H), 6.81 (d, *J* = 8.8 Hz, 4H), 2.99 (s, 12H).

^{13}C NMR not measured due to limited solubility.

IR (neat): 805, 948, 1192, 1220, 1457, 1606, 2925, 3054.

HRMS (ESI–TOF, $[M+H]^+$) *m/z* calcd. for $C_{52}H_{36}N_4O_2S_2$ 813.2352; Found 813.2313.

S3 - Nuclear Magnetic Resonance

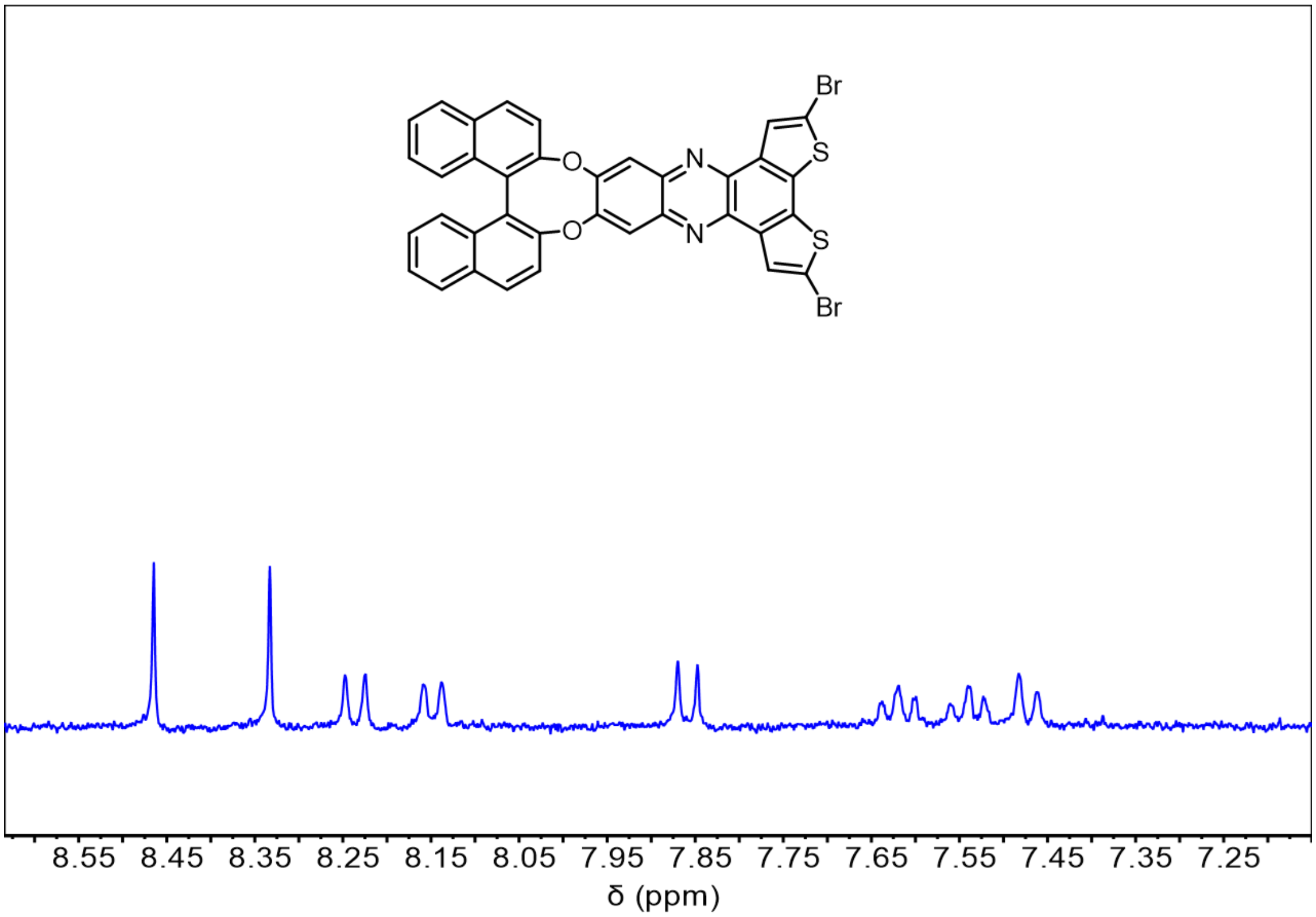

Figure 1. Zoomed in ^{1}H NMR spectrum of **DTP-Br**.

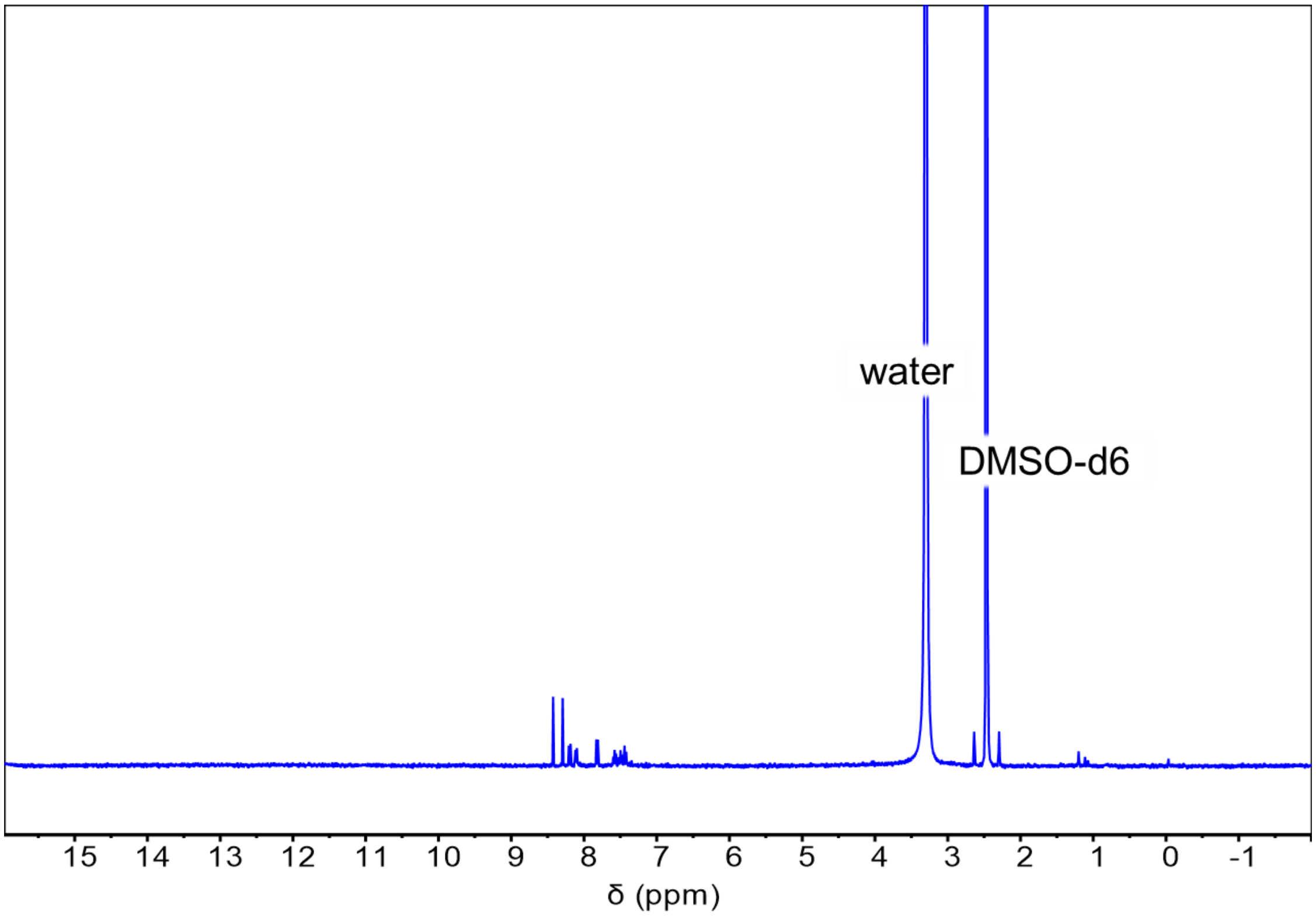

Figure 2. Full ^{1}H NMR spectrum of **DTP-Br**.

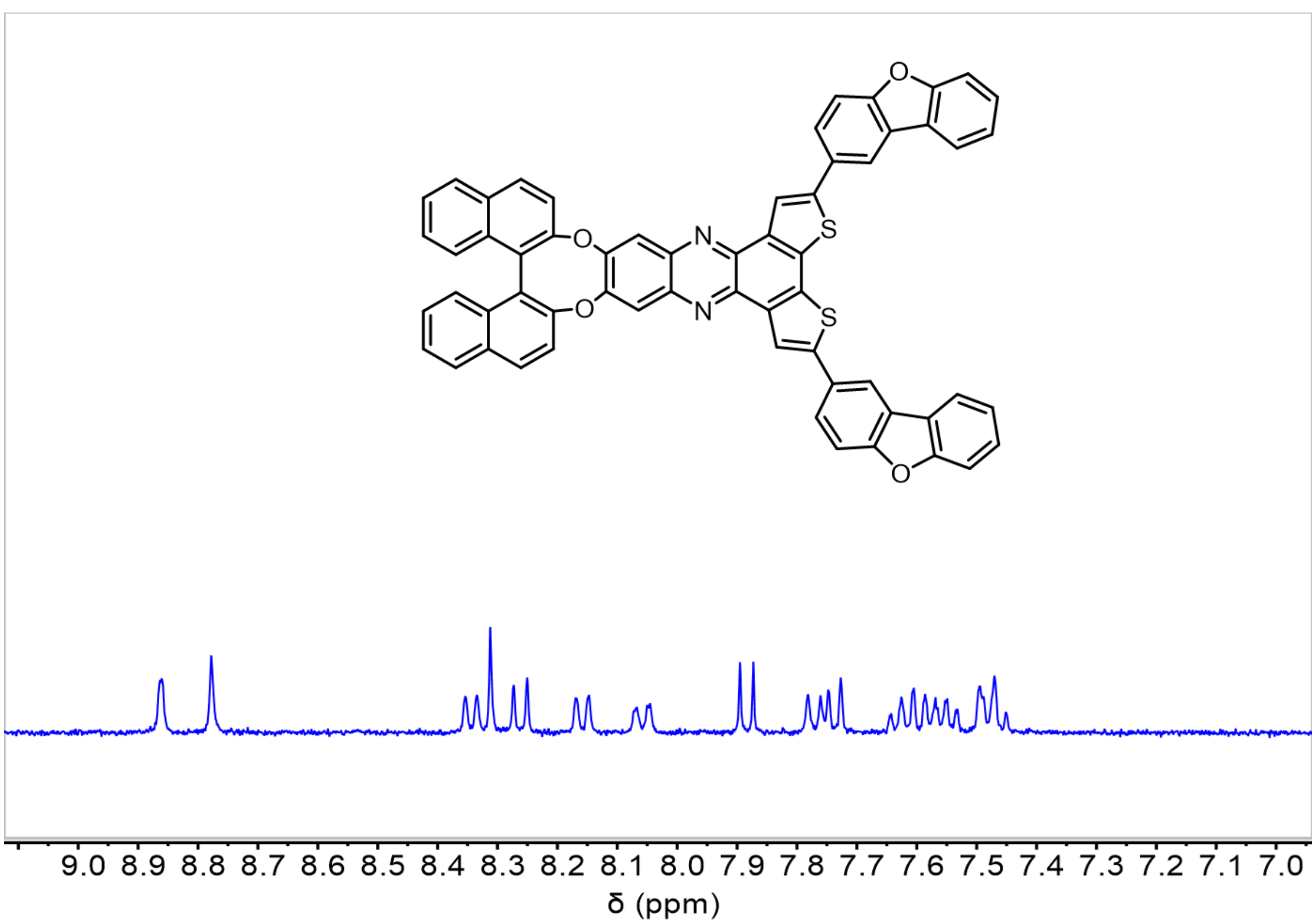

Figure 3. Zoomed in ^{1}H NMR of **DTP-dbf**.

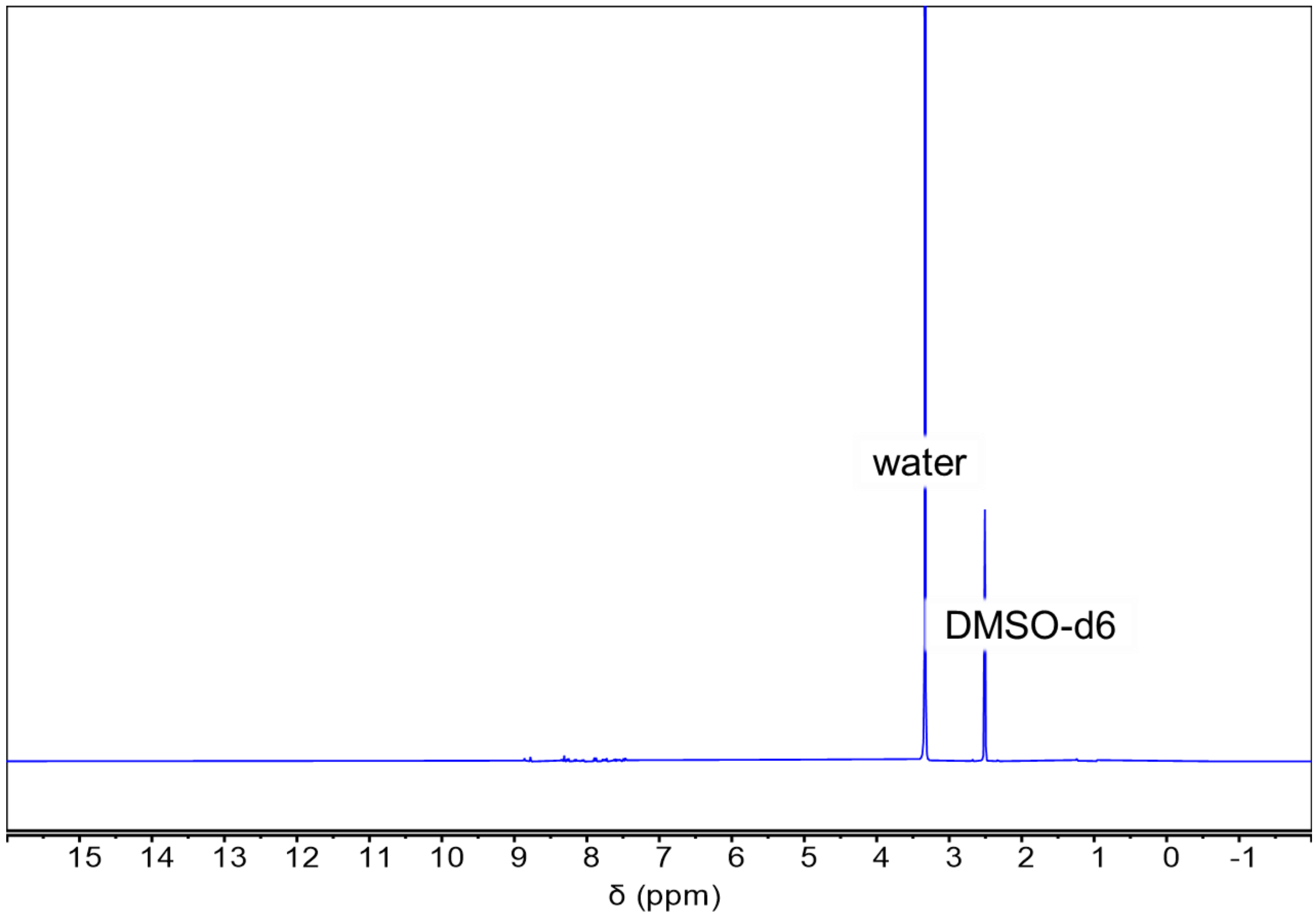

Figure 4. Full ^{1}H NMR of **DTP-dbf**.

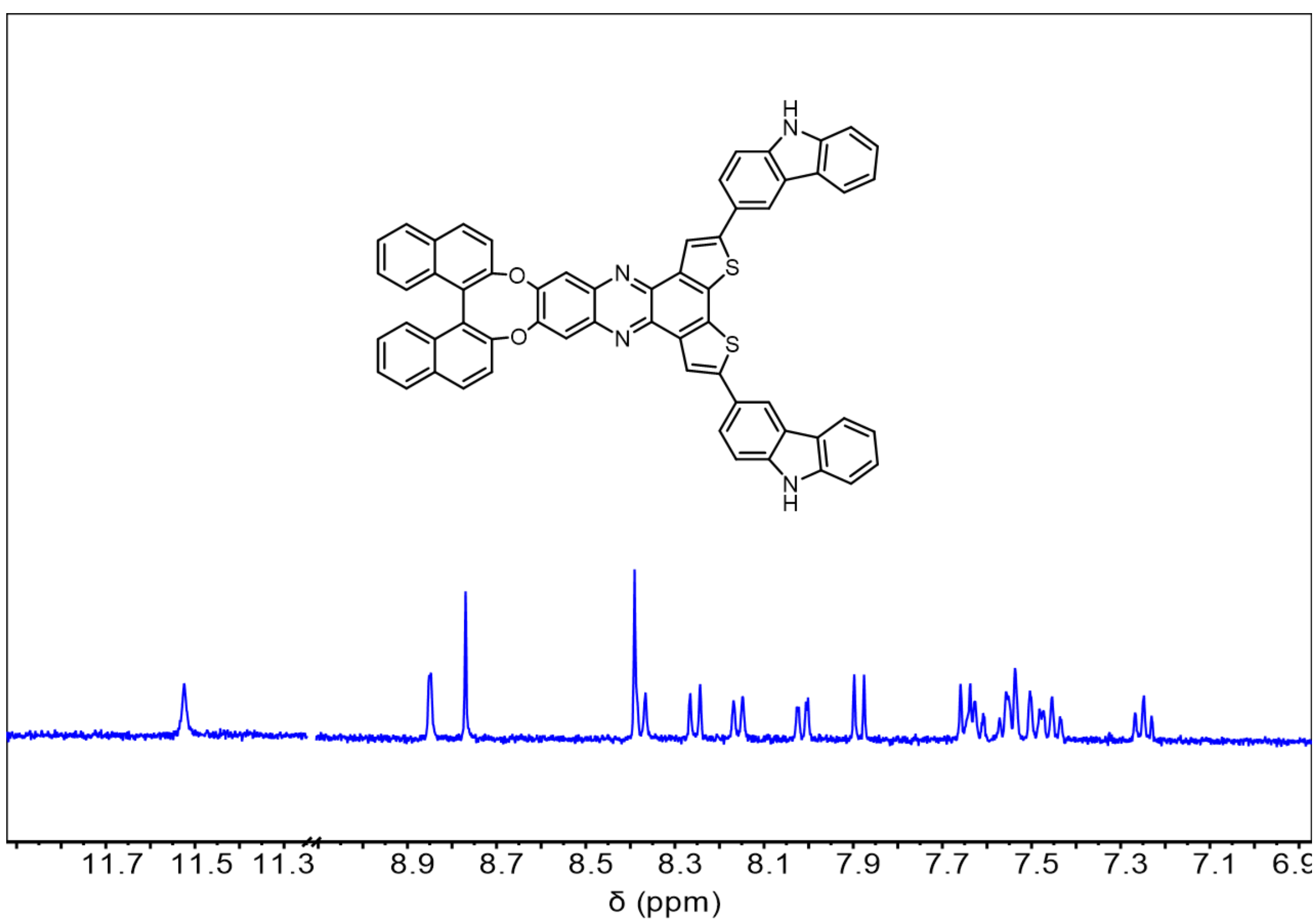

Figure 5. Zoomed in ^{1}H NMR spectrum of **DTP-cbz**.

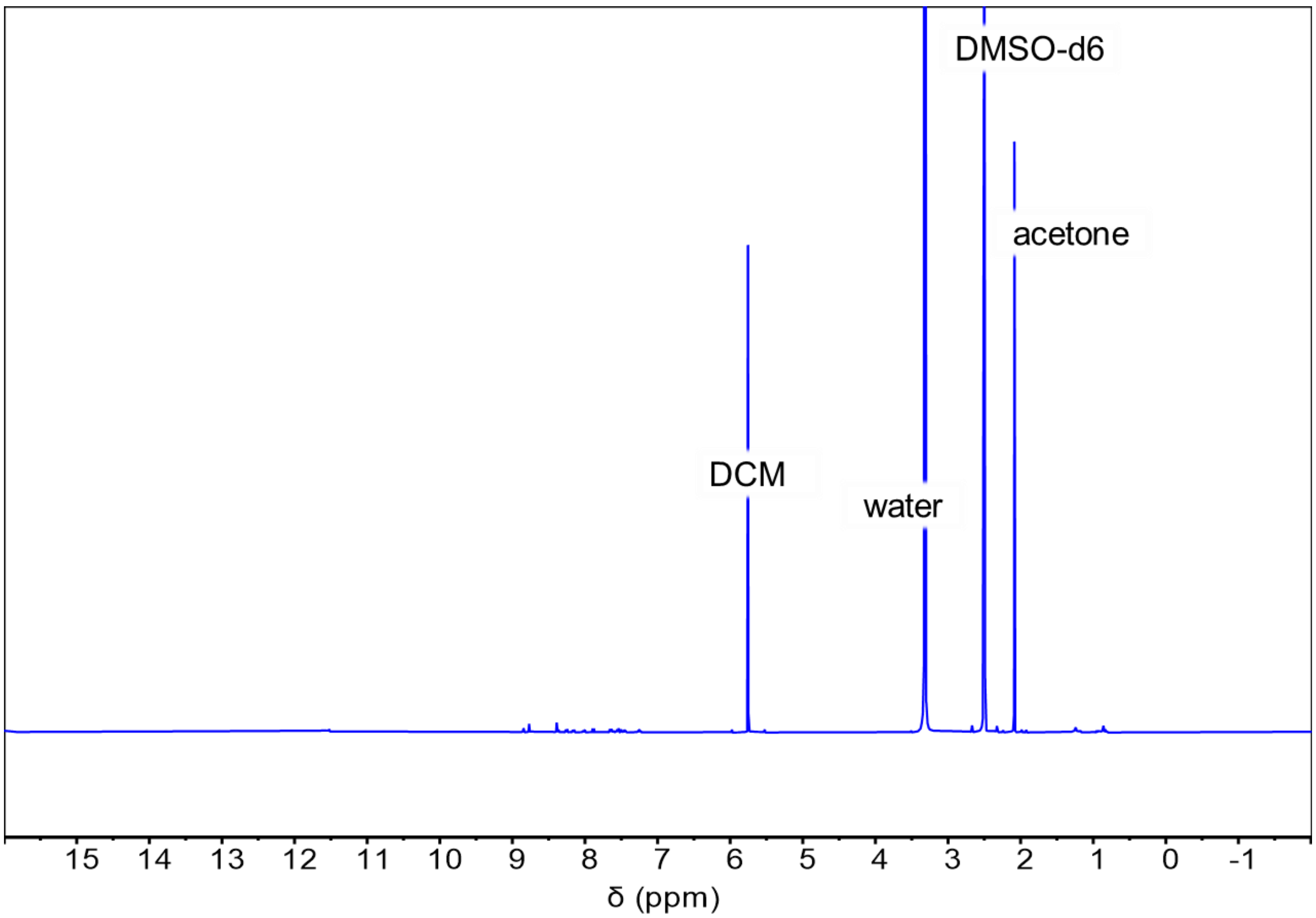

Figure 6. Full ^{1}H NMR spectrum of **DTP-cbz**.

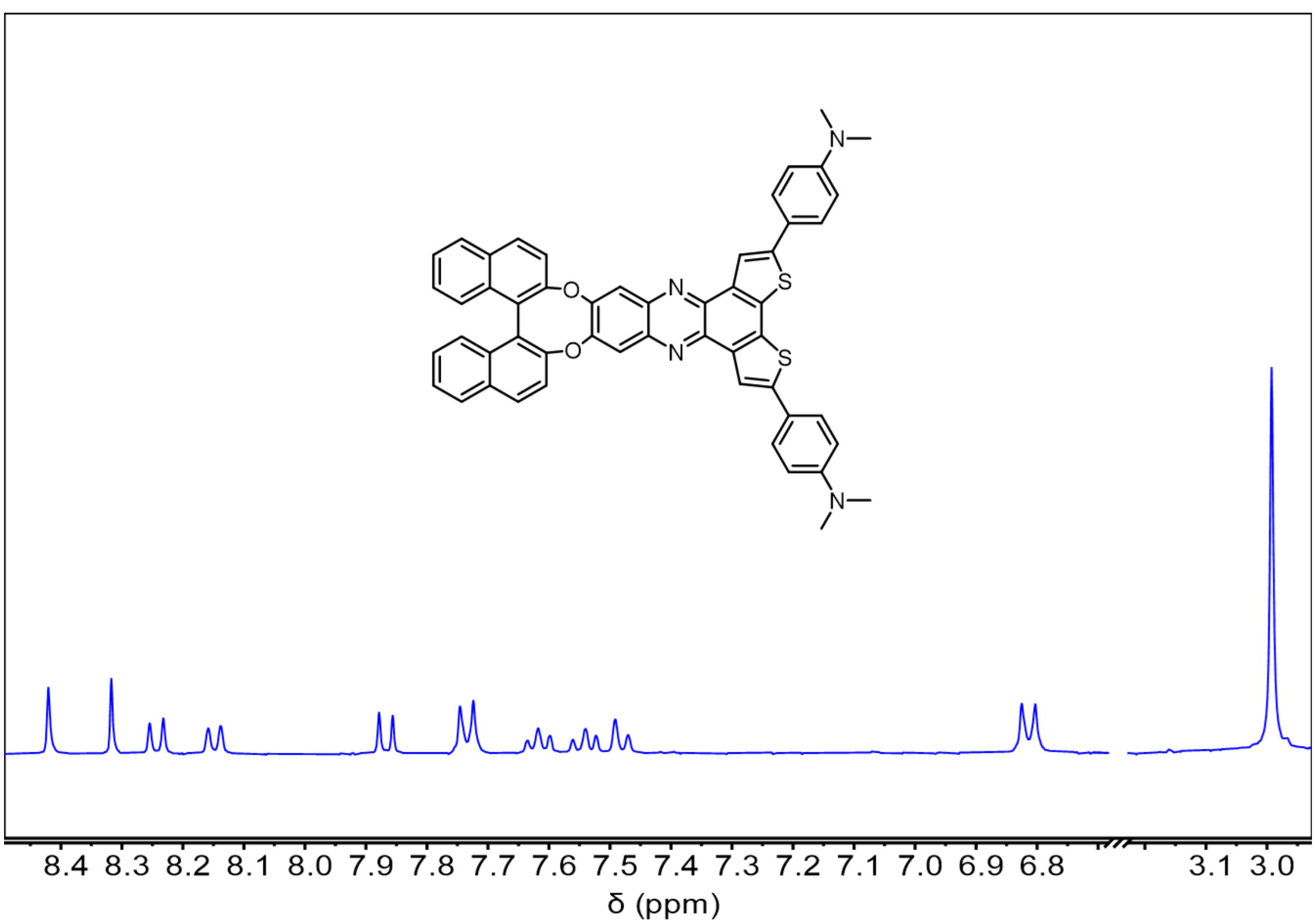

Figure 7. Zoomed in ^{1}H NMR of **DTP-dma**.

$Note$: Residual CH_2Cl_2 and water do appear as signals in this spectrum but have been clipped for clarity so only compounds peaks are shown. Full spectrum is below and includes all signals.

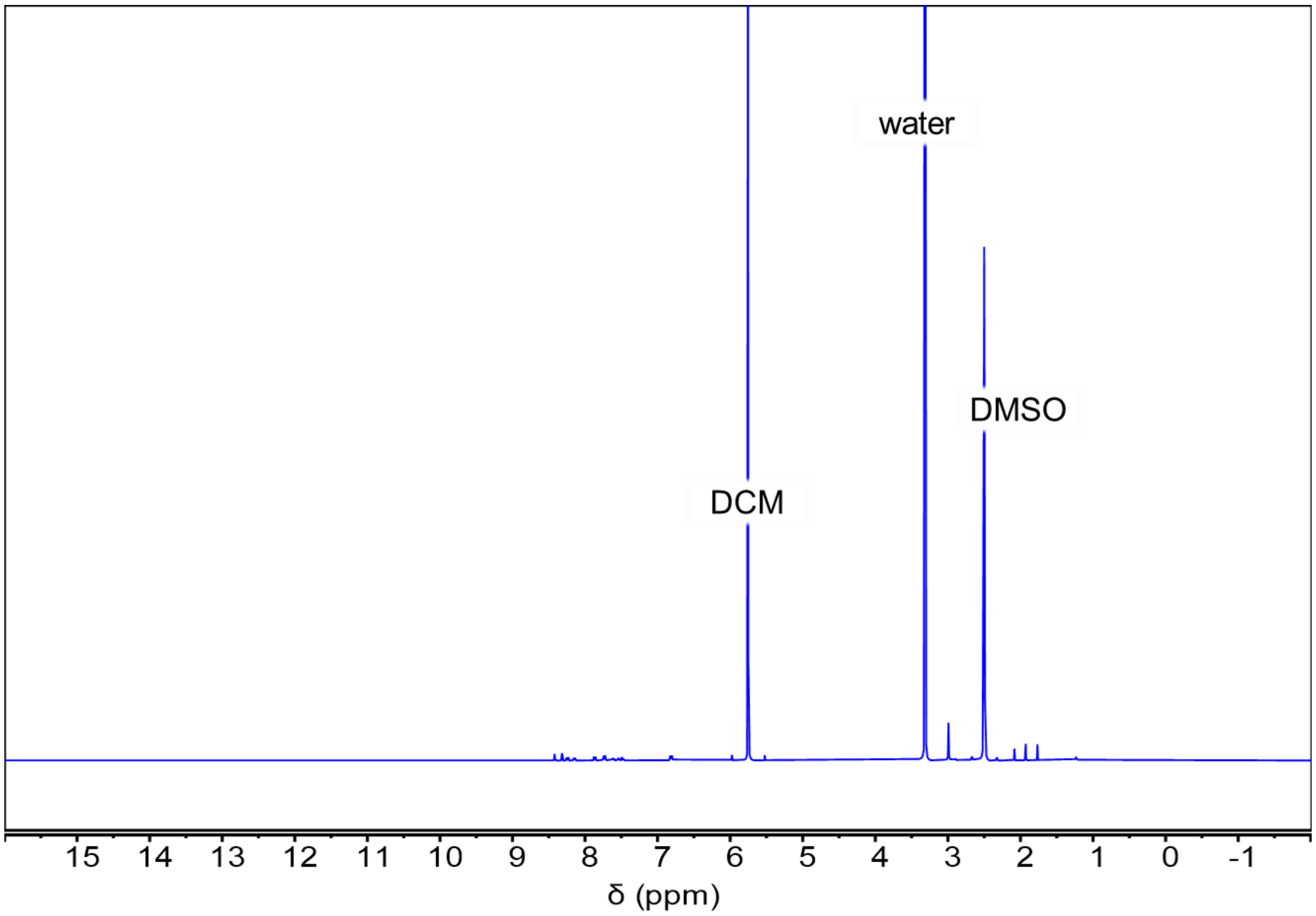

Figure 8. Full ^{1}H NMR spectrum of **DTP-dma**.

S4 – Electroanalytical Characterization

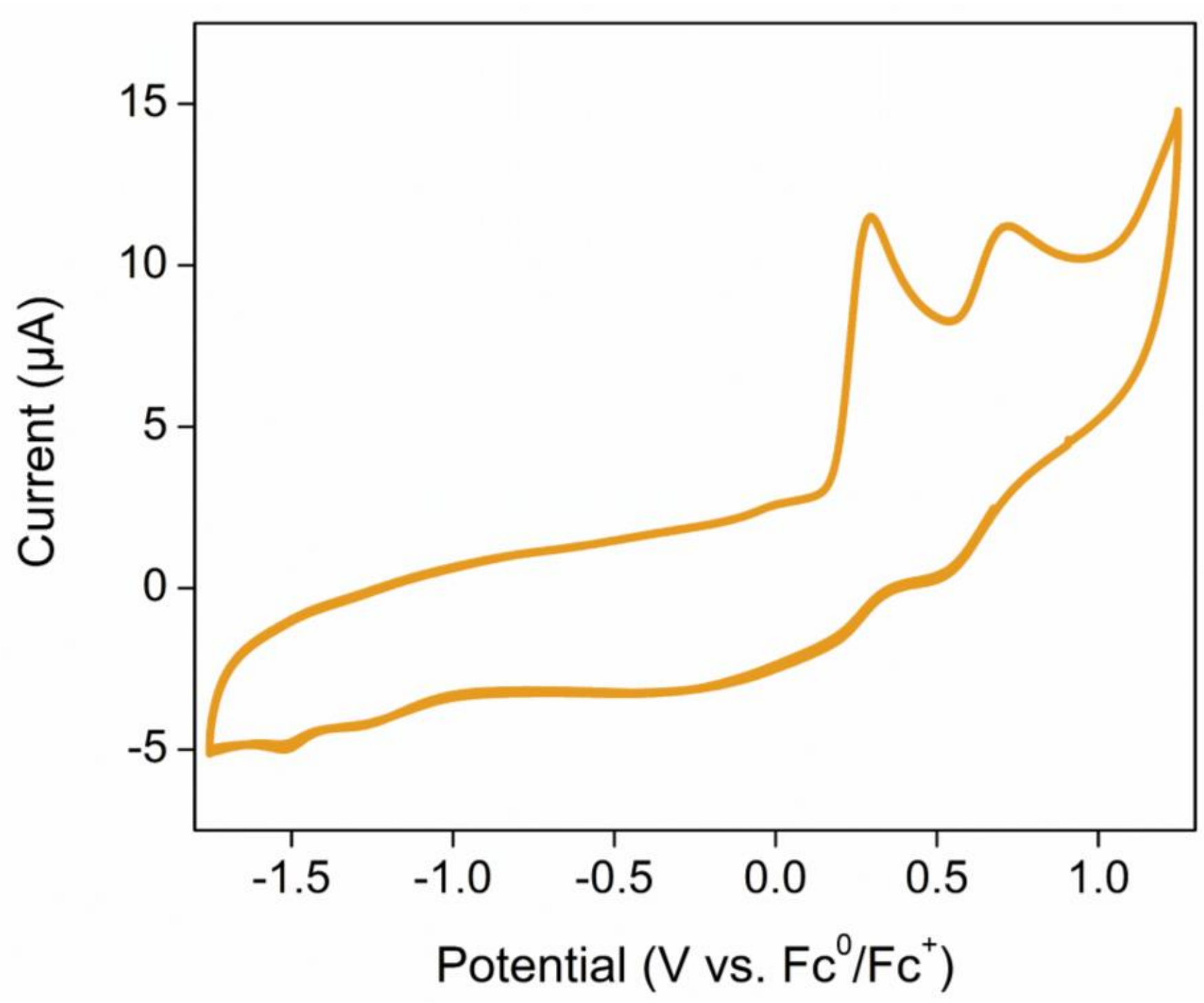

Figure 9. Cyclic voltammogram of **DTP-Br** in MeCN.

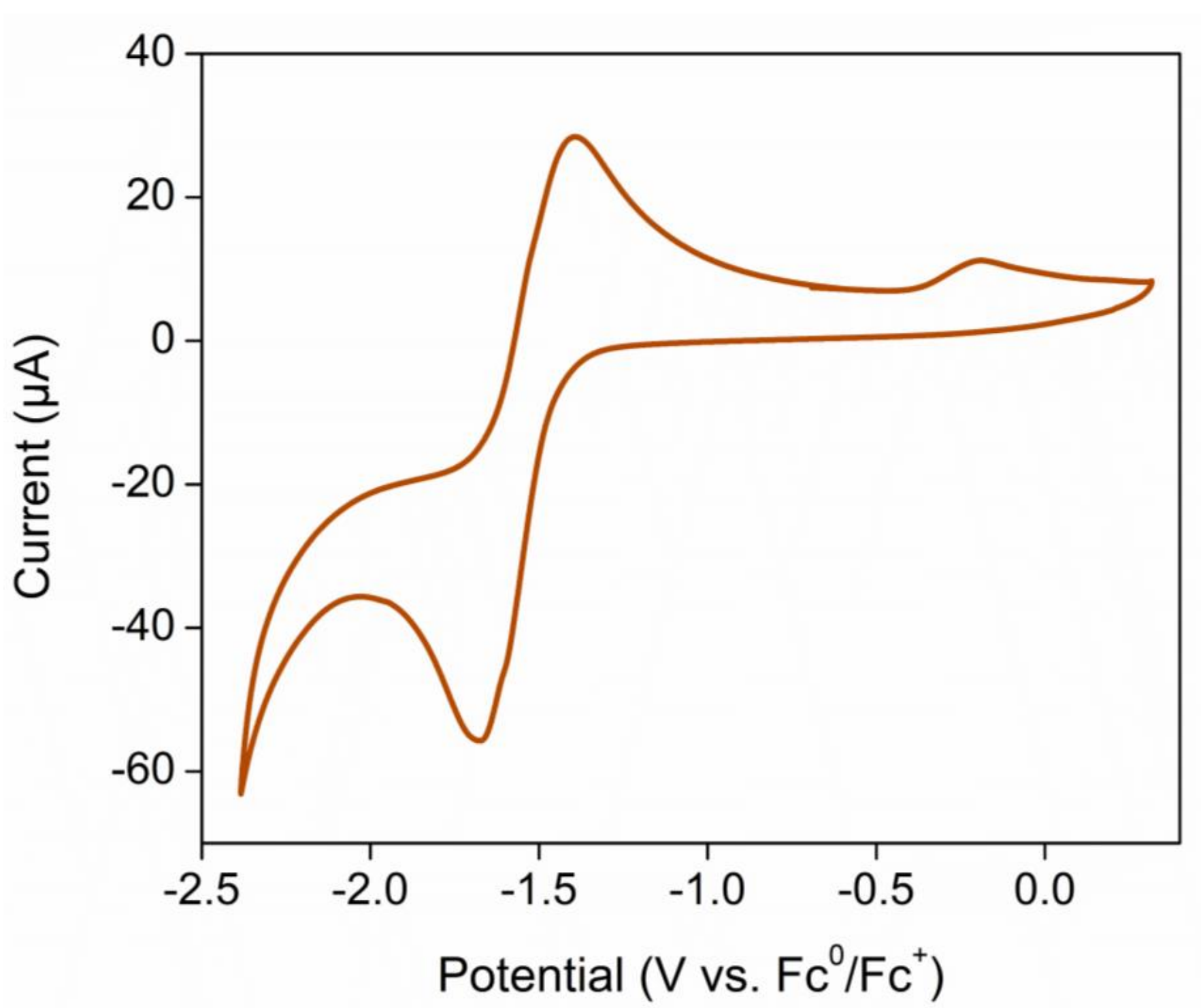

Figure 10. Cyclic Voltammogram of **DTP-dbf** in MeCN.

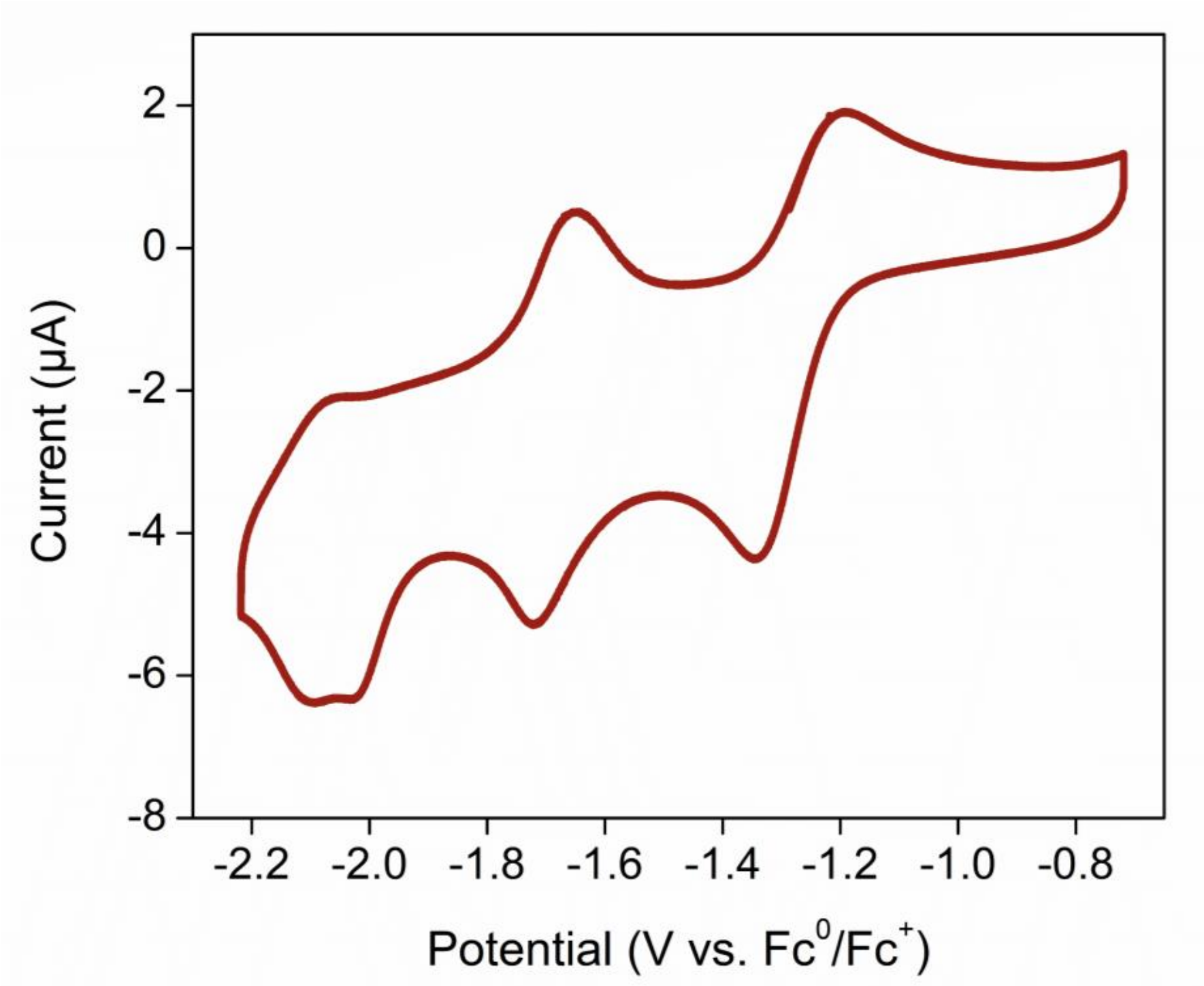

Figure 11. Cyclic voltammogram of **DTP-cbz** in MeCN.

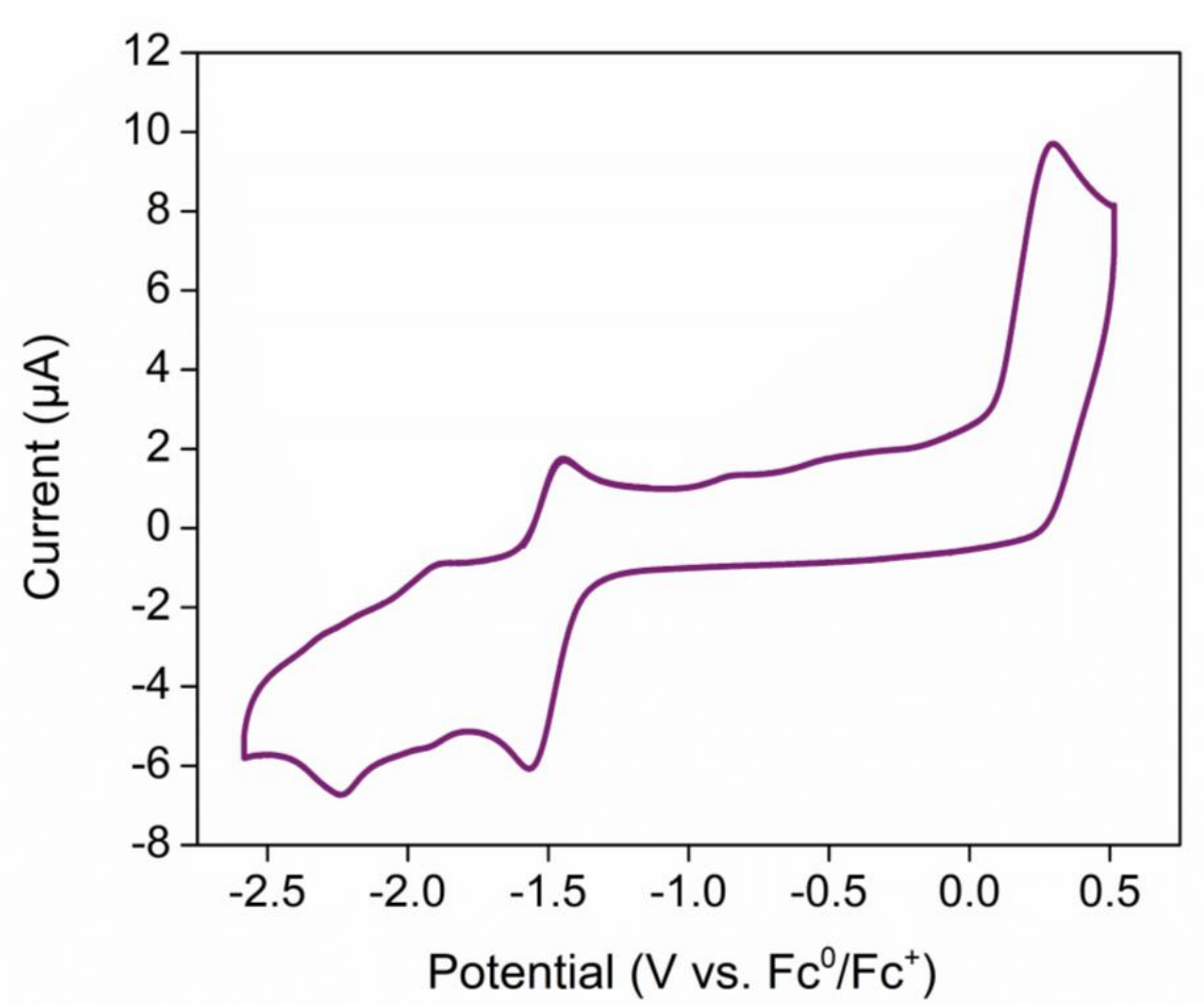

Figure 12. Cyclic voltammogram of **DTP-dma** in MeCN.

S5 – Electronic Circular Dichroism

All circular dichroism spectra were recorded in CH_2Cl_2 at 25 °C (c~ 10^{-6} M) unless otherwise stated. All protonated CD spectra were acidified with ~ 500 µL TFA unless otherwise stated.

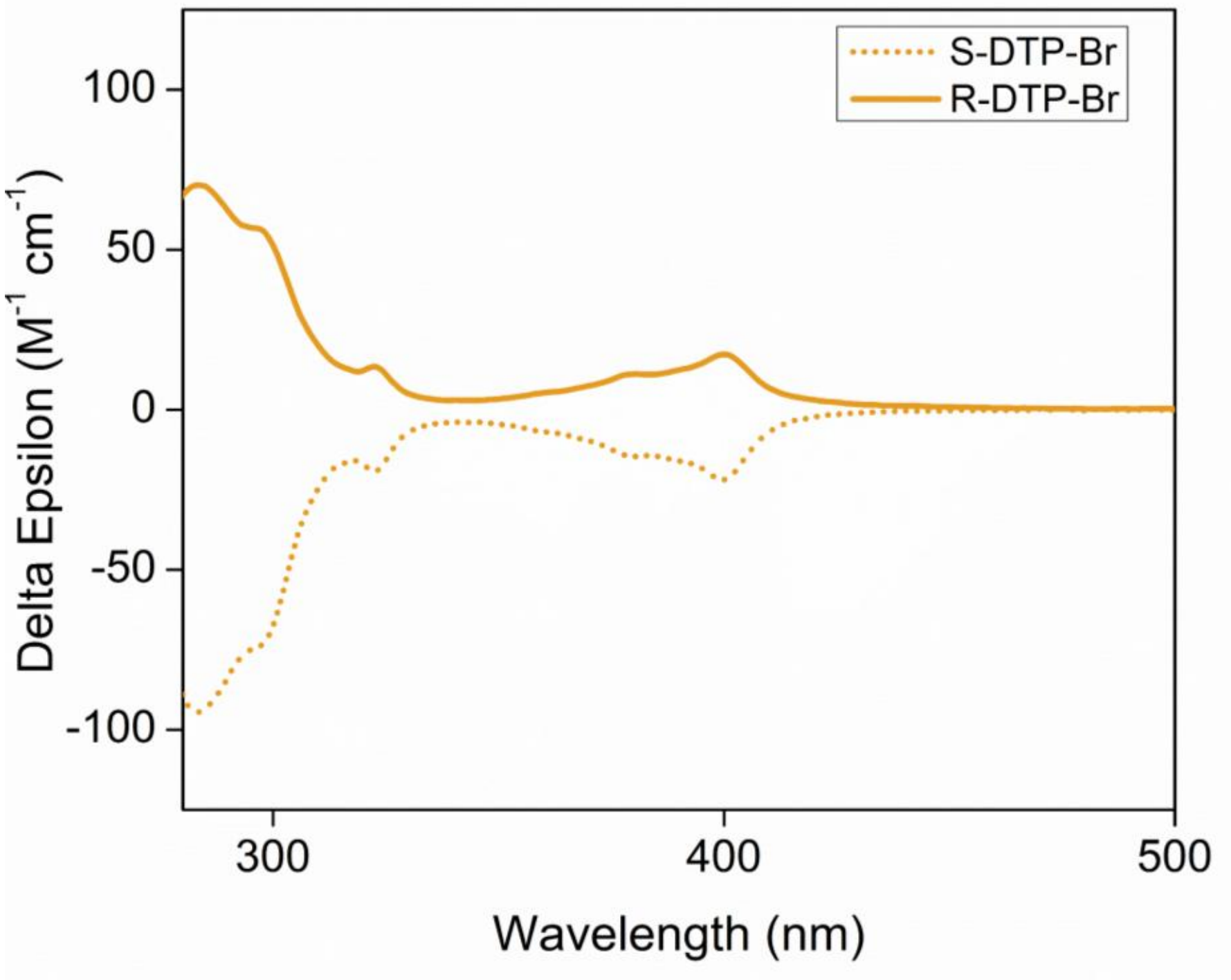

Figure 13. Neutral CD spectrum of **DTP-Br**.

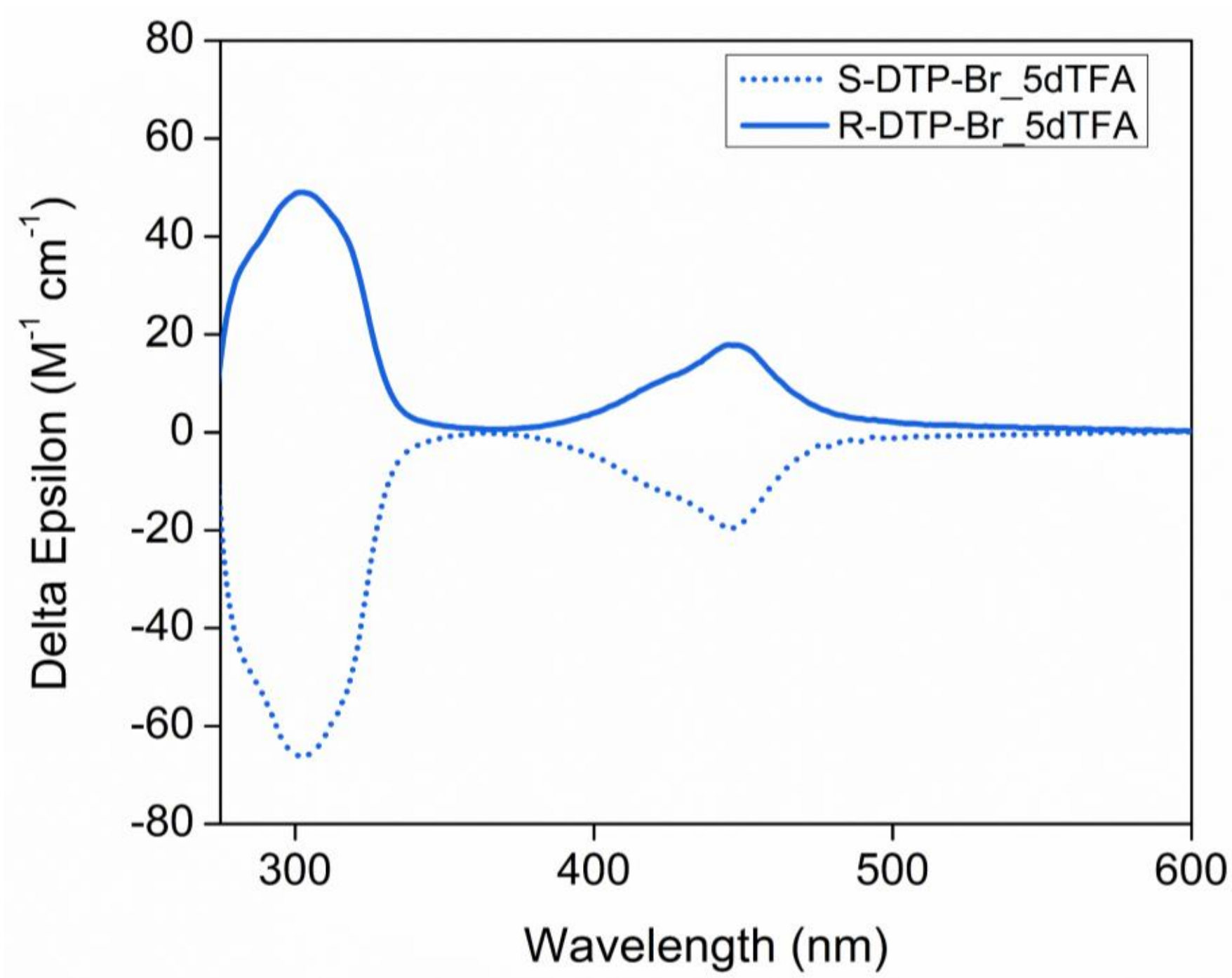

Figure 14. Protonated CD spectrum of **DTP-Br**.

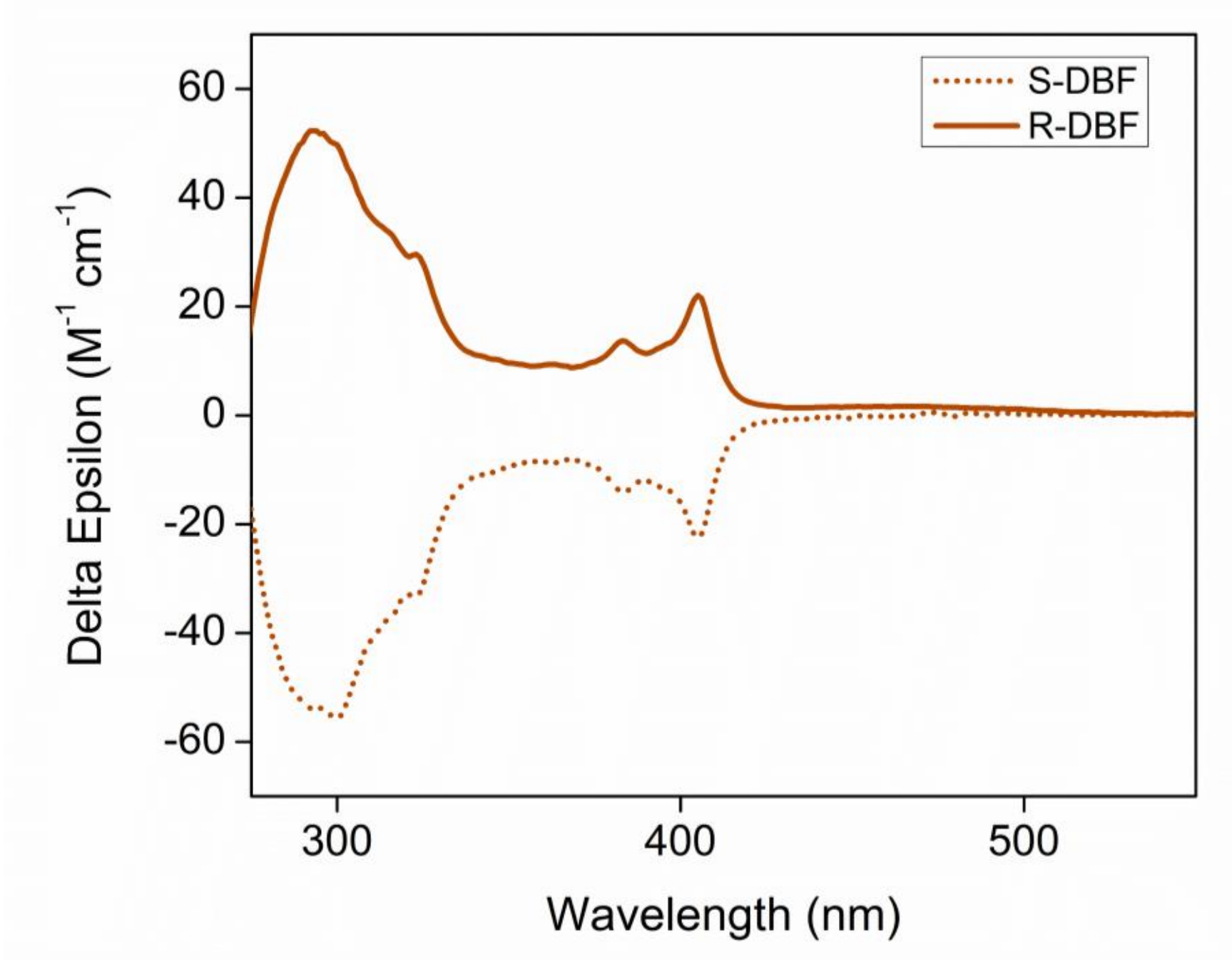

Figure 15. Neutral CD spectrum of **DTP-dbf**.

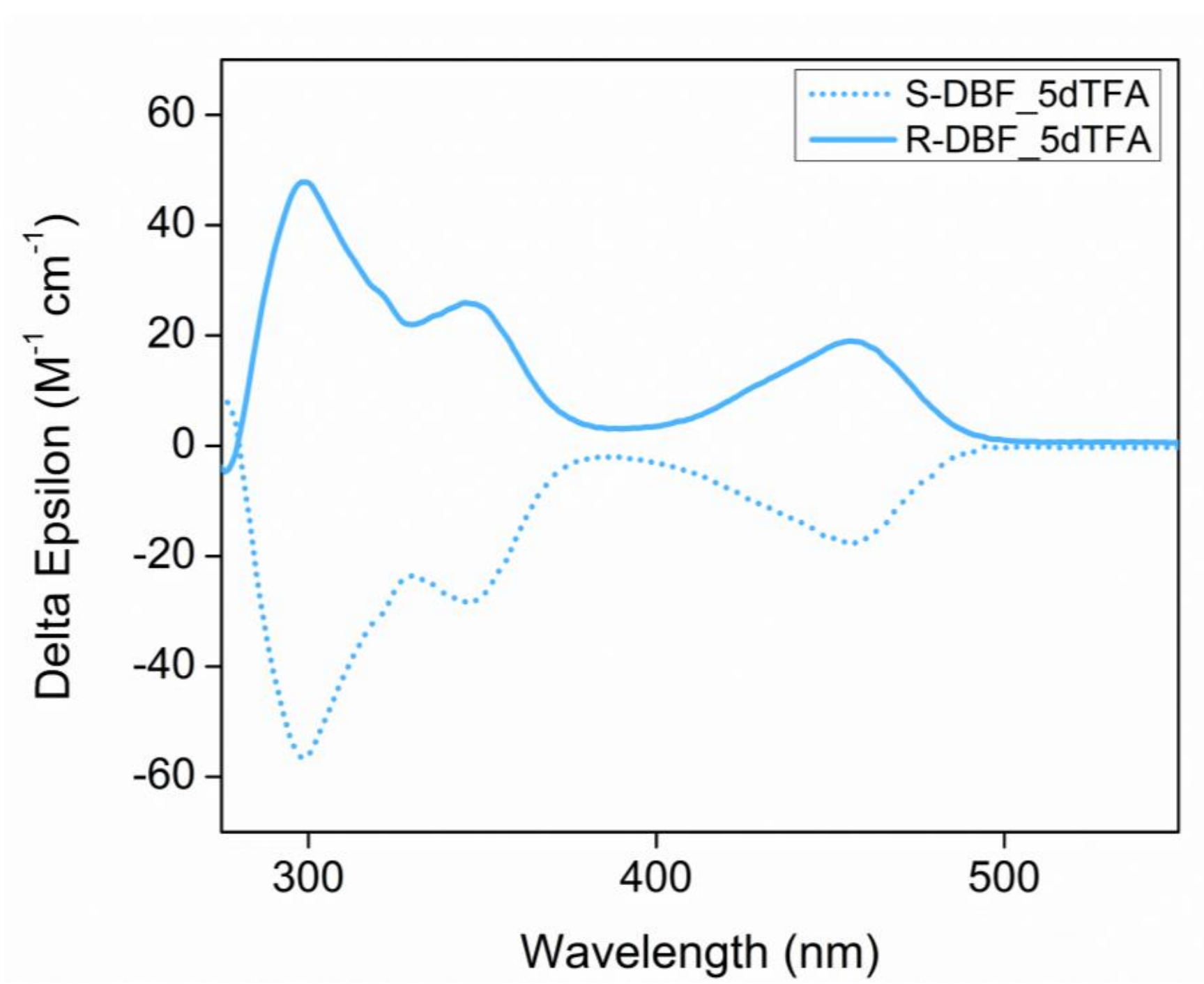

Figure 16. Protonated CD spectrum of **DTP-dbf**.

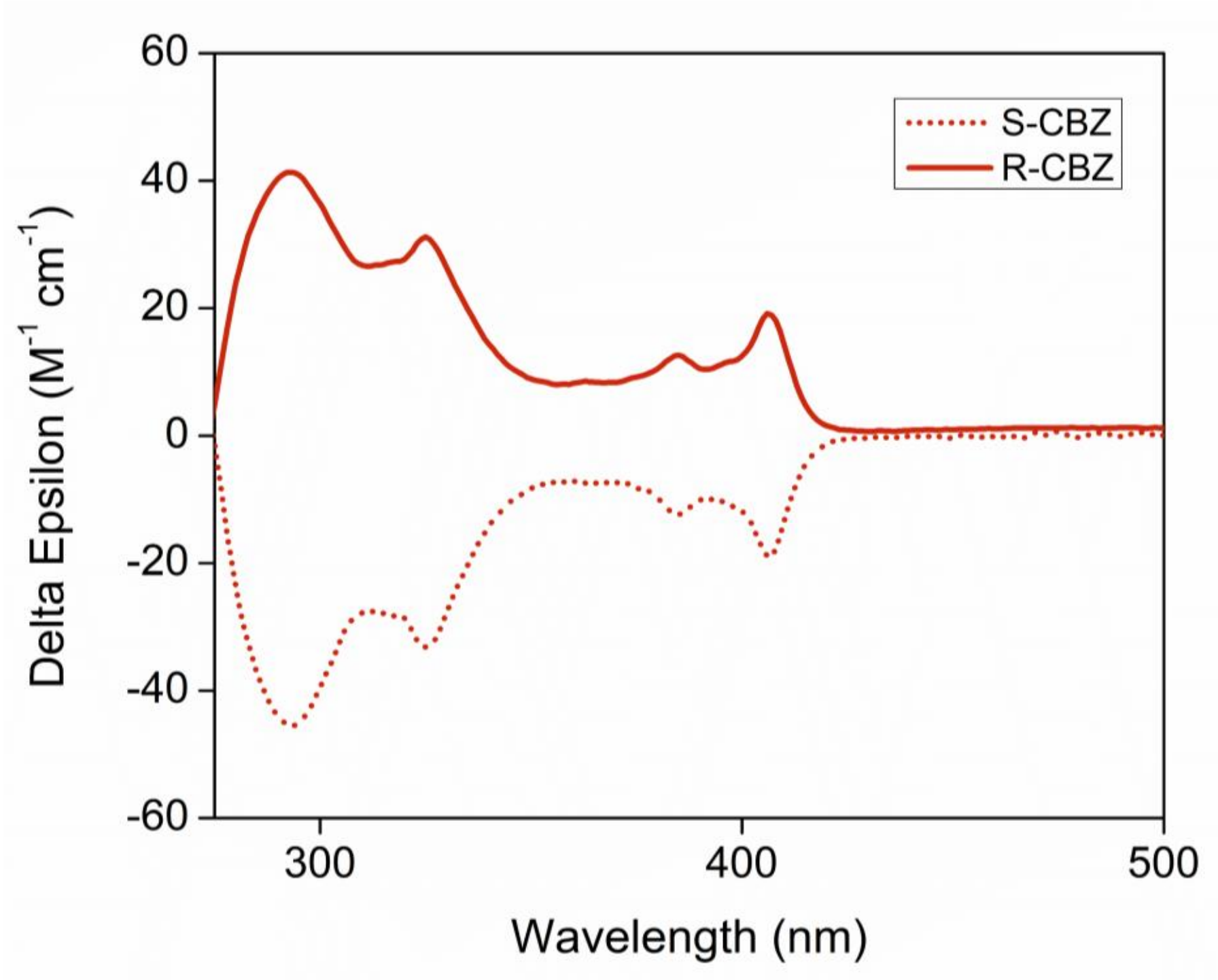

Figure 17. Neutral CD spectrum of **DTP-cbz**.

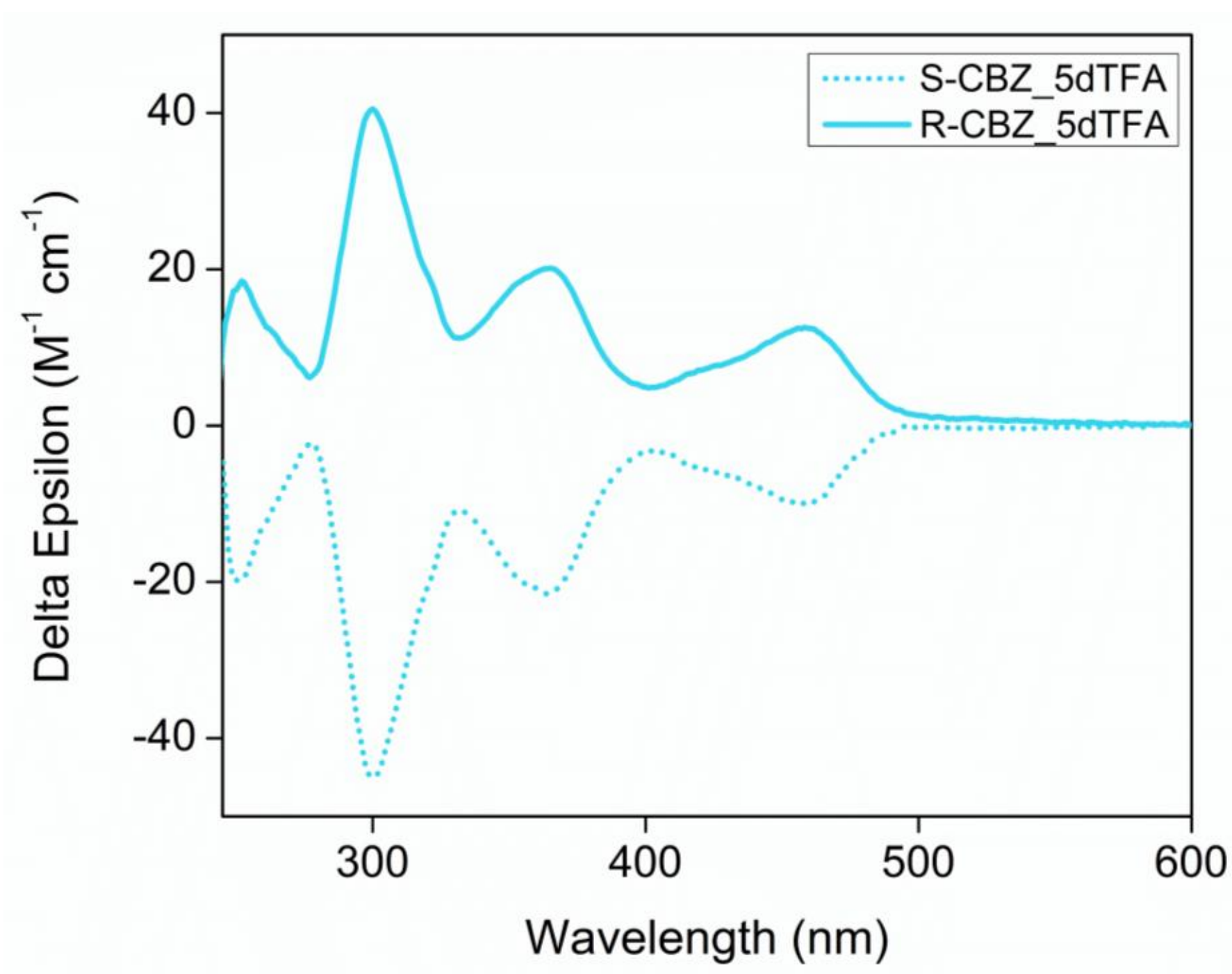

Figure 18. Protonated CD spectrum of **DTP-cbz**.

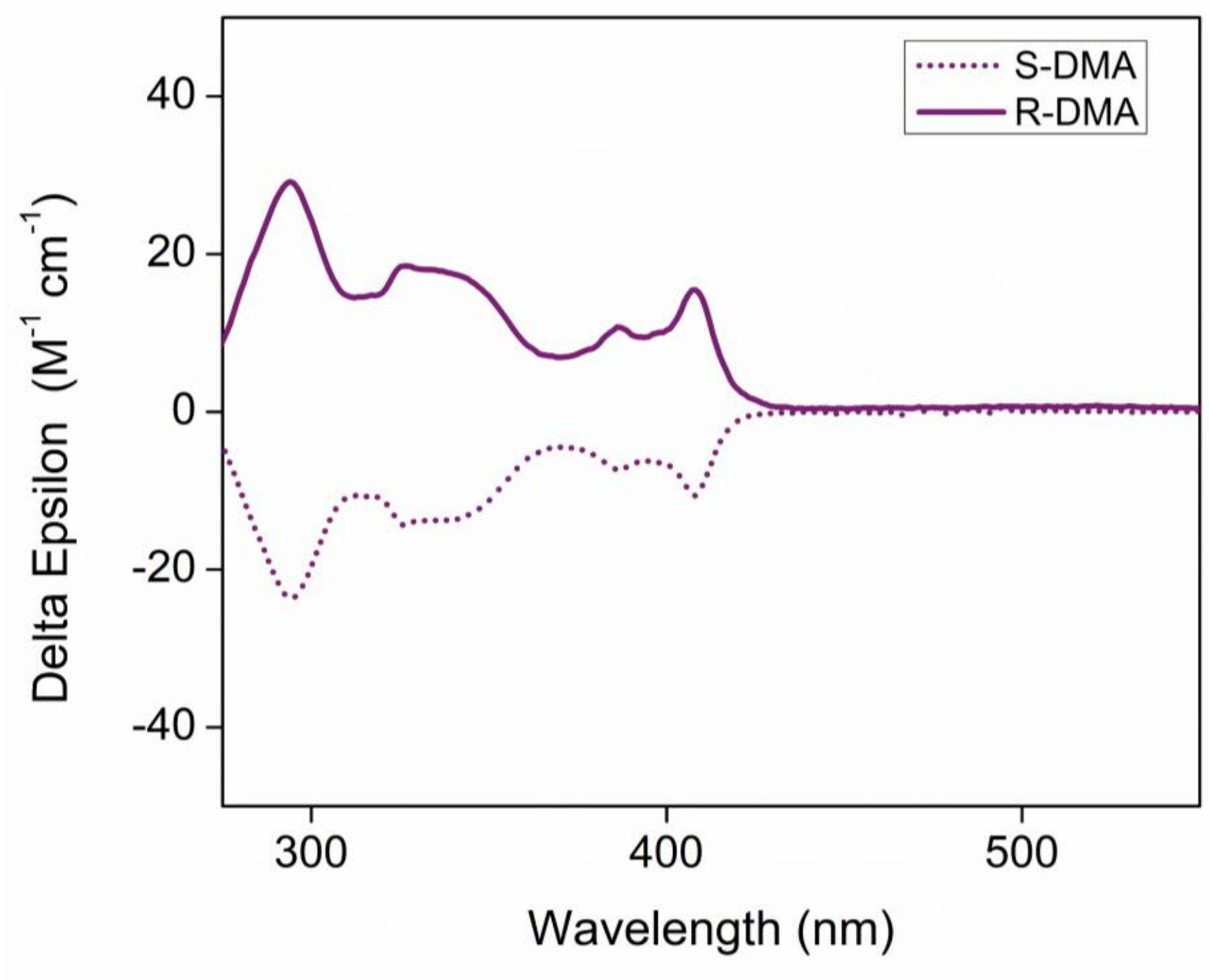

Figure 19. Neutral CD spectrum of **DTP-dma**.

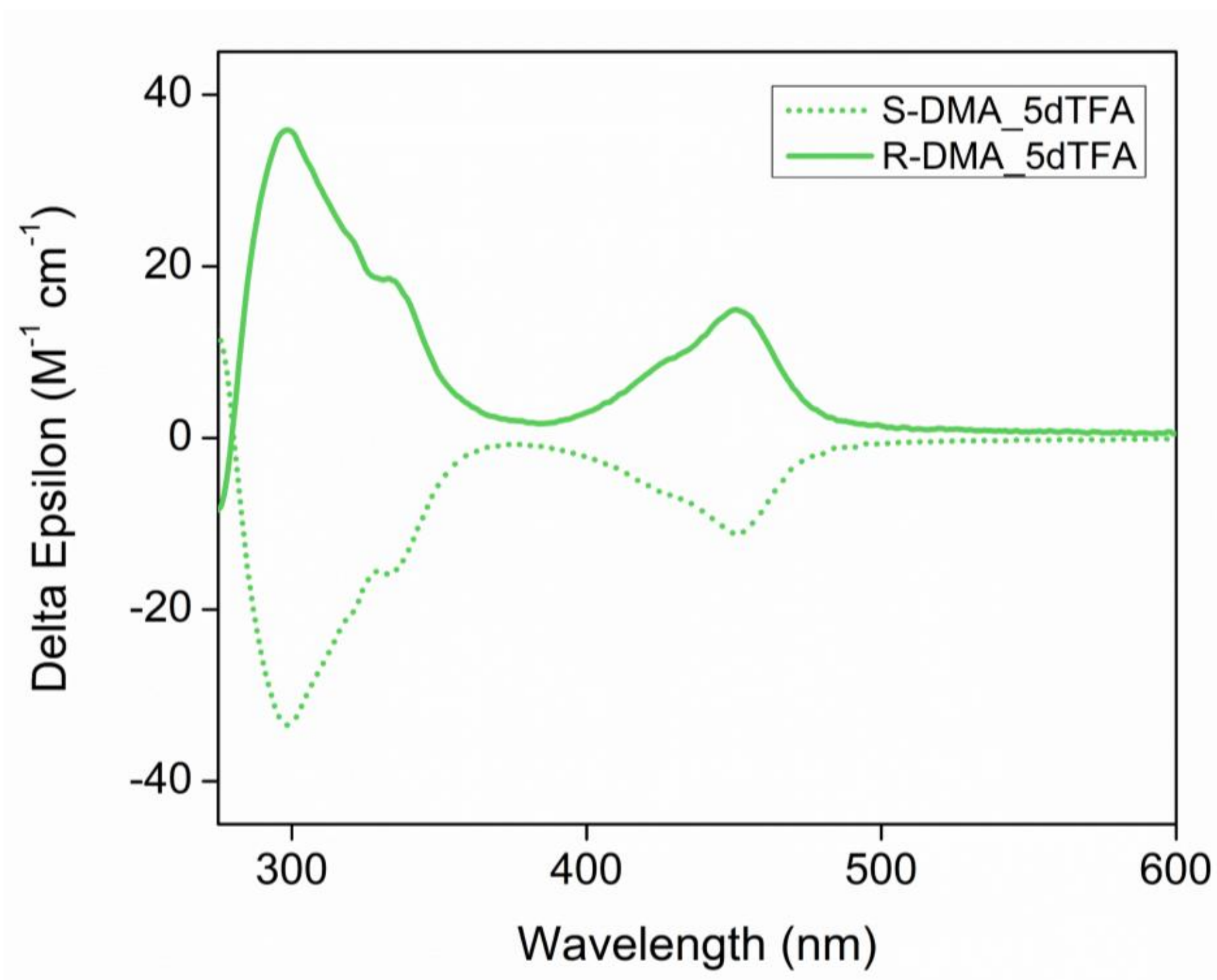

Figure 20. Protonated CD spectrum of **DTP-dma**.

Solid State CD

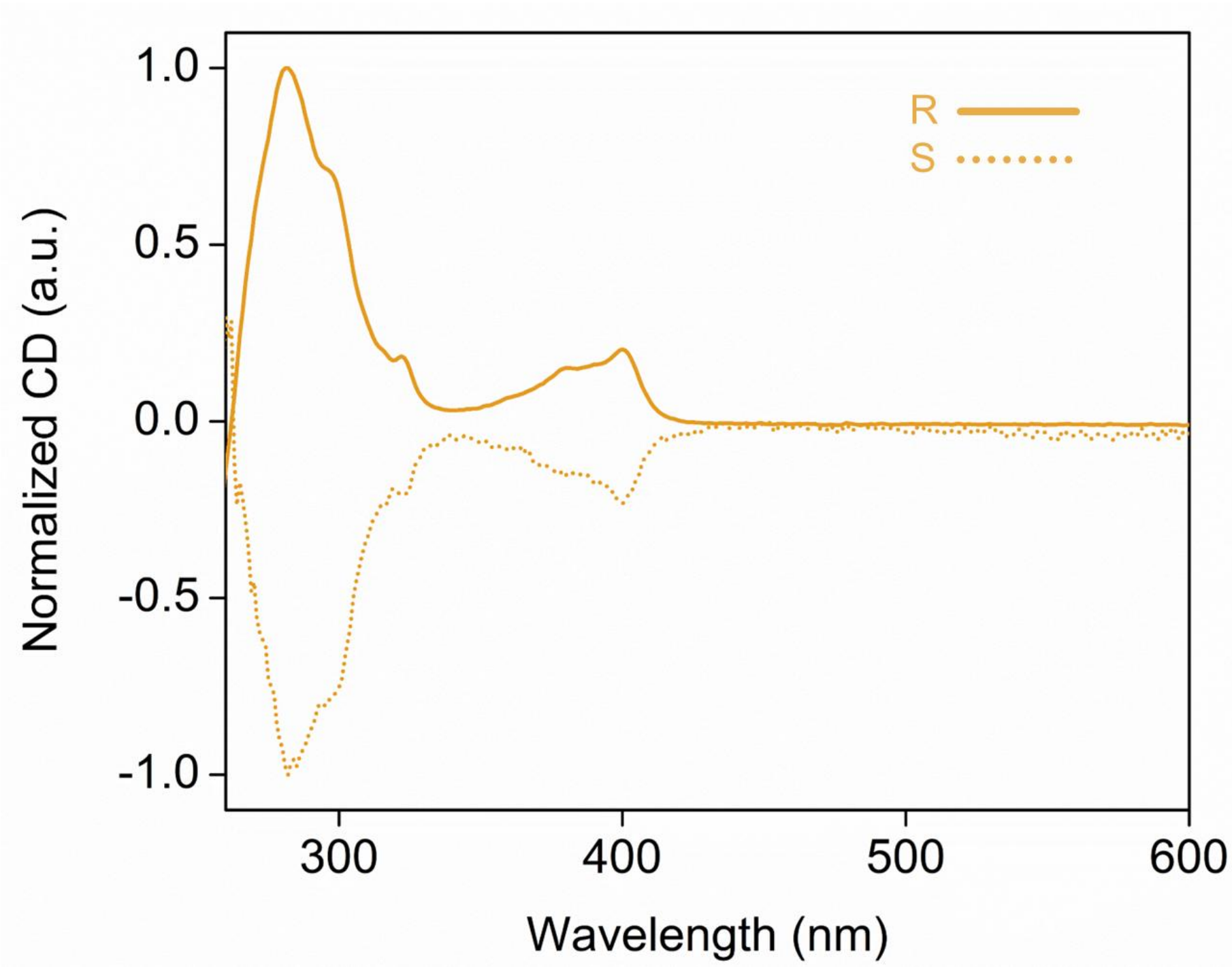

Figure 21. Solid-state circular dichroism spectrum of **DTP-Br** in a PMMA matrix.

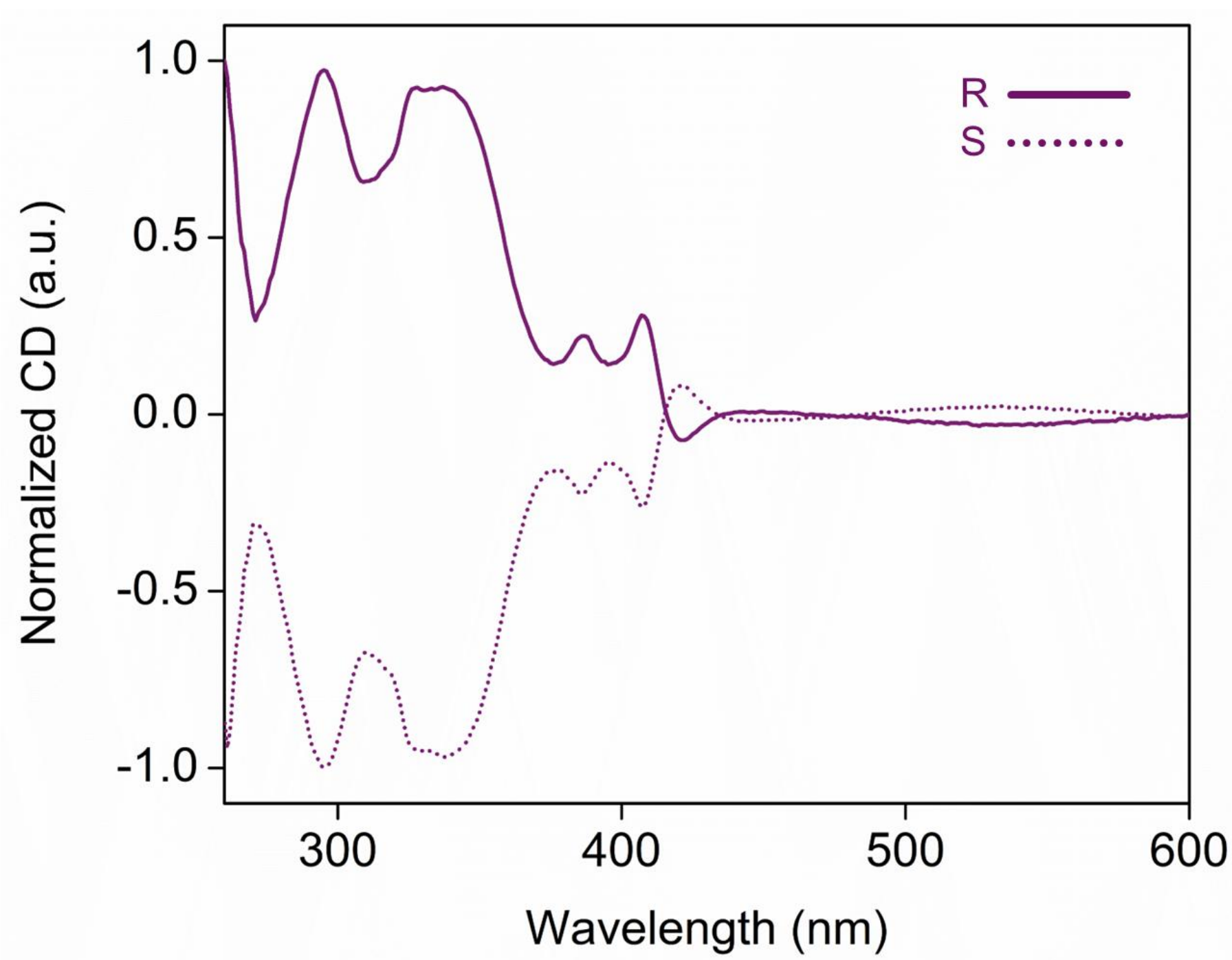

Figure 22. Solid-state circular dichroism spectrum of **DTP-dma** in a PMMA matrix.

S6 – Computational Details

DFT calculations were performed using ORCA 6.0.0.[3-8] Structures were optimized at the PBE/def2-TZVP level of theory, and frequency calculations were used to ensure proper minimization had occurred. Time-dependent density functional theory calculations were performed using ORCA 6.0.0 at the PBE0/def2-TZVP level of theory, incorporating 30 states. Qualitatively equivalent results were obtained using cam-B3LYP for S0 and S1 structure optimization, as well as TD-DFT calculations. In all cases, the NTO for the electron to hole transition suggest charge-transfer character dominates the transition. In **DTP-dma** and **DTP-cbz**, the delocalization of the electron extends all over the carbazole and aniline moieties. In **DTP-dbf** on the other hand, the delocalization is limited to the arene ring forming the biphenyl linkage. This could potentially explain the observed difference in photoluminescence, as a better orbital overlap between S1 and S0 is indicative of higher emission rates.

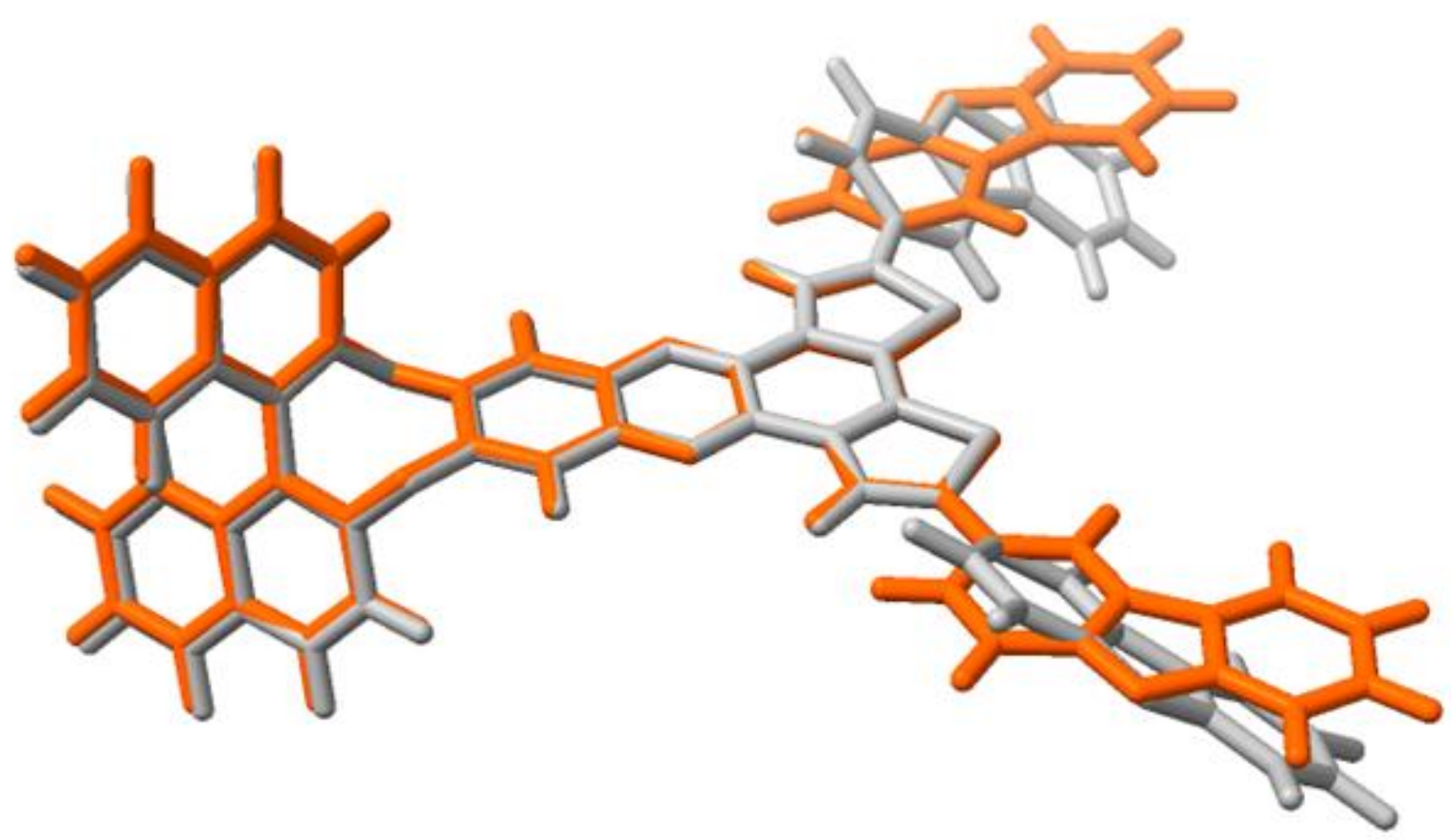

Figure 23. S_0 (grey) and S_1 (orange) geometry for **DTP-dbf**.

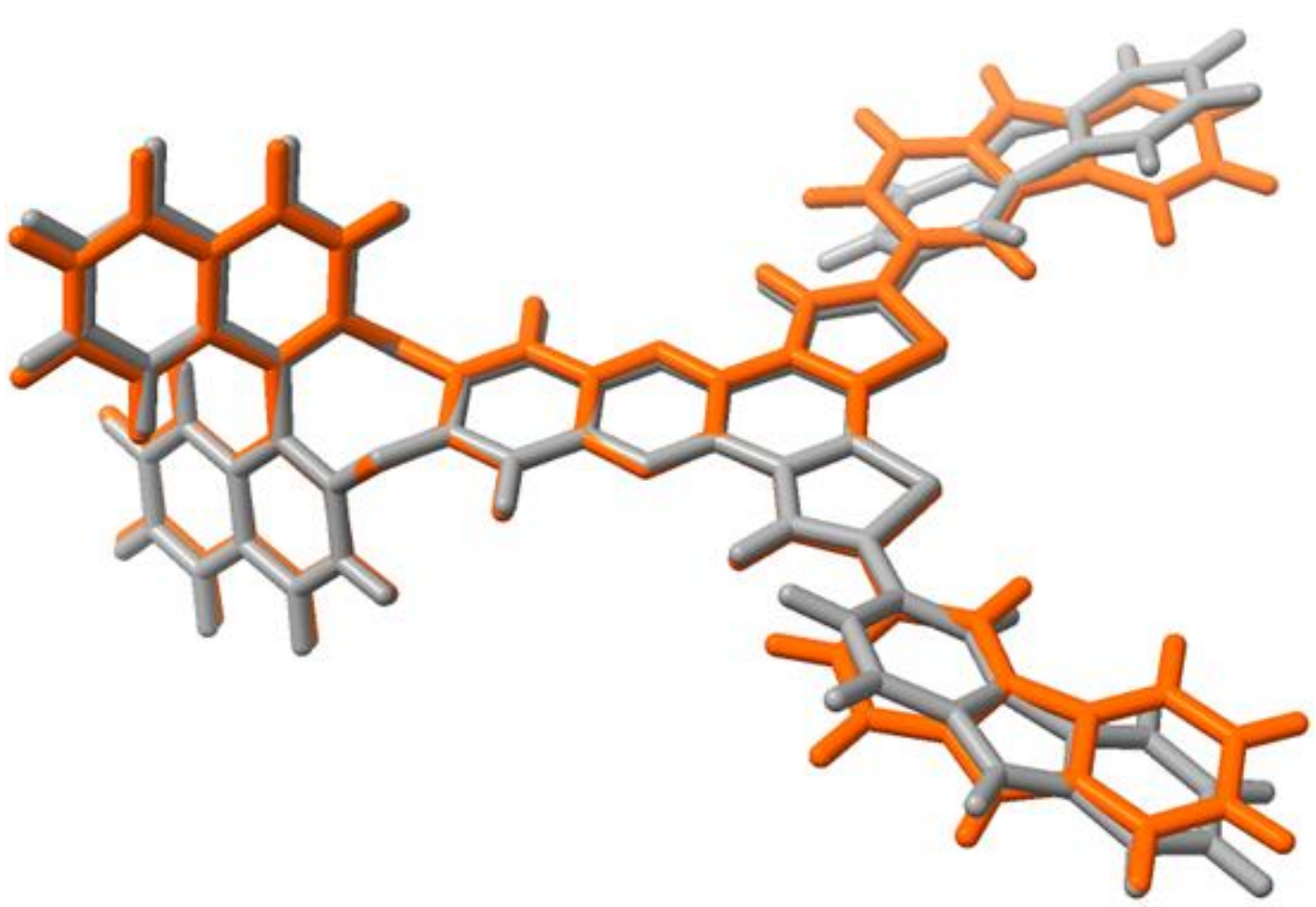

Figure 24. S_0 (grey) and S_1 (orange) geometry for **DTP-cbz**.

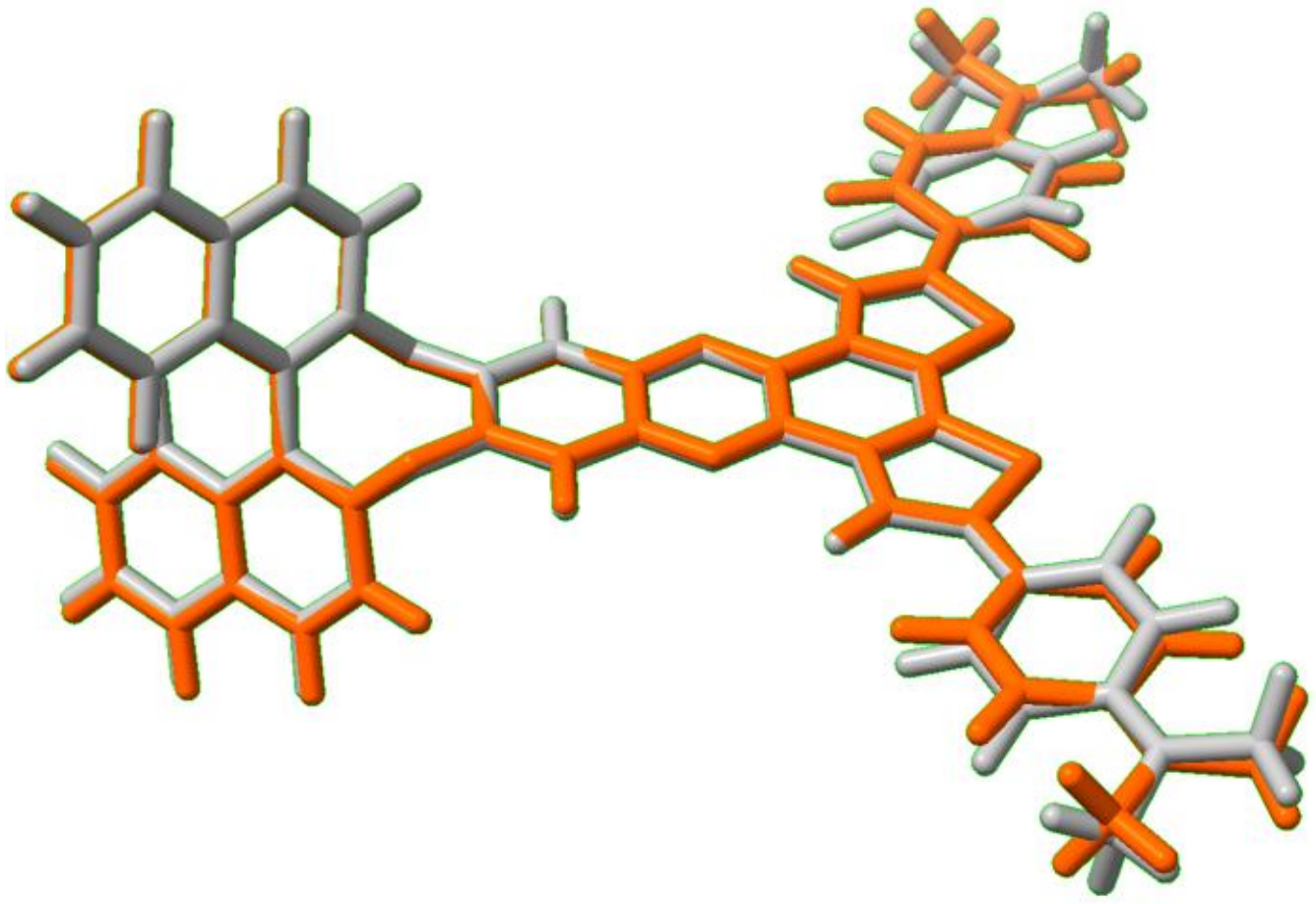

Figure 25. S_0 (grey) and S_1 (orange) geometry for **DTP-dma**.

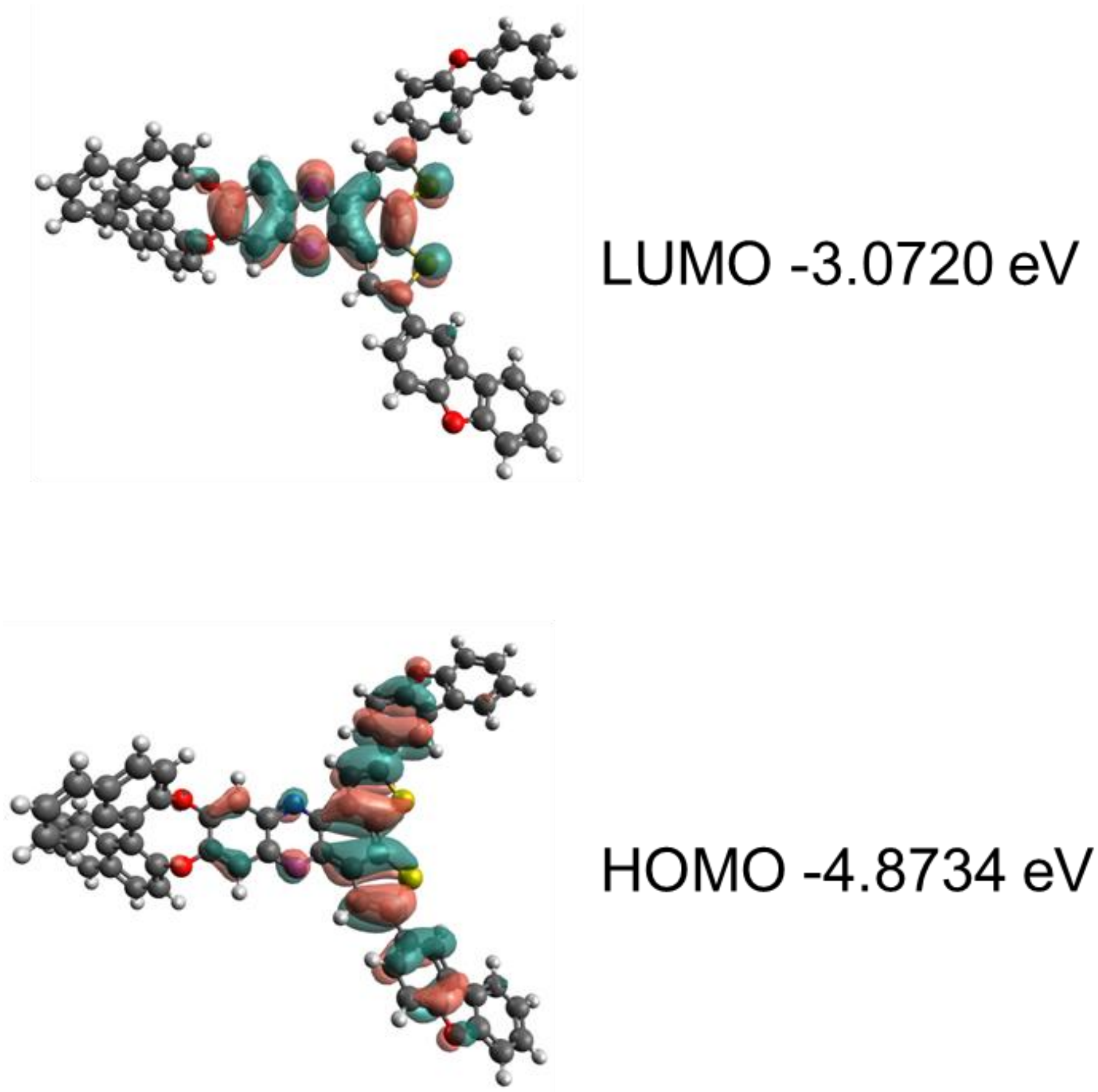

Figure 26. LUMO and HOMO values and orbital depictions for **DTP-dbf**.

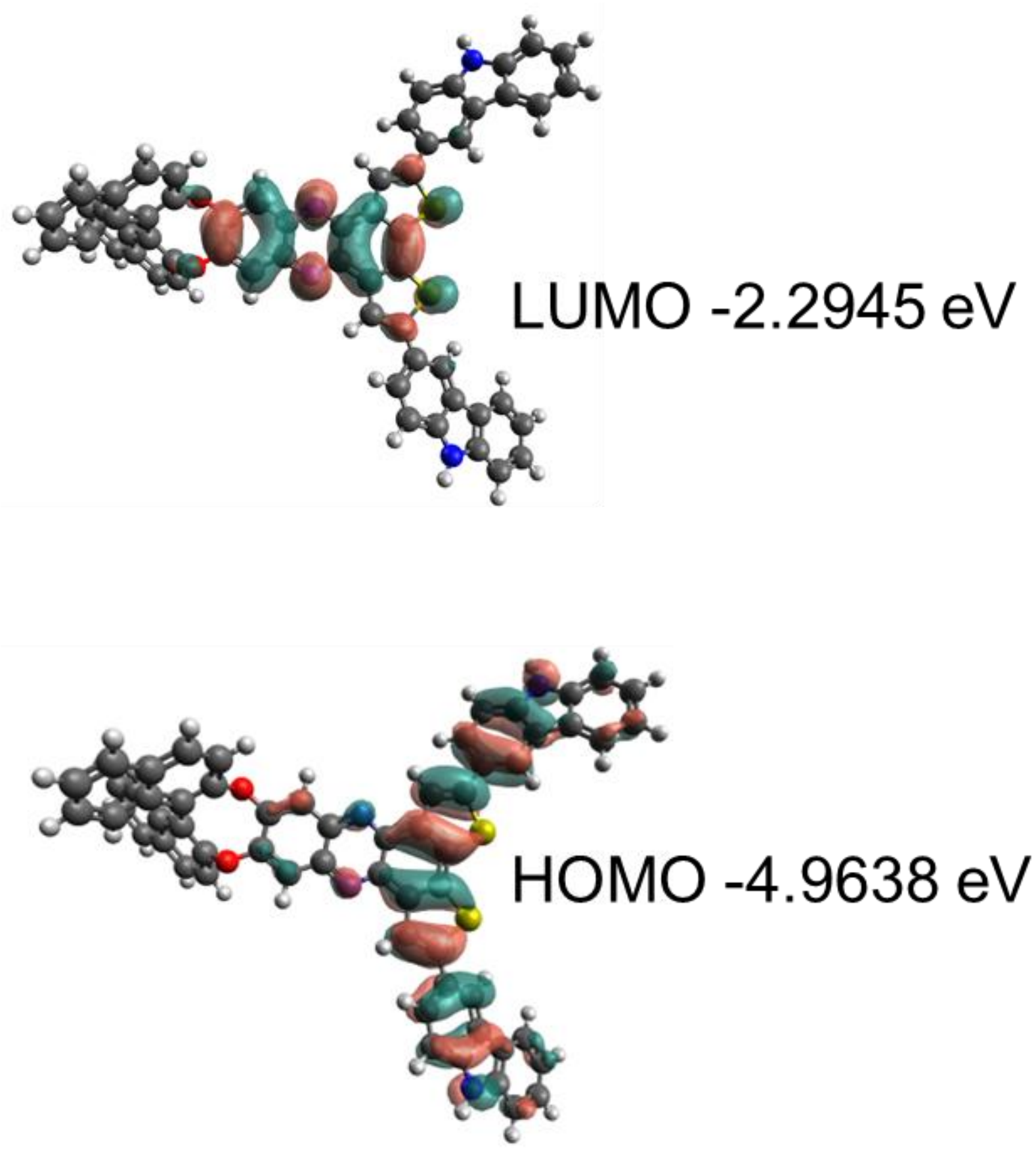

Figure 27. LUMO and HOMO values and orbital depictions for **DTP-cbz**.

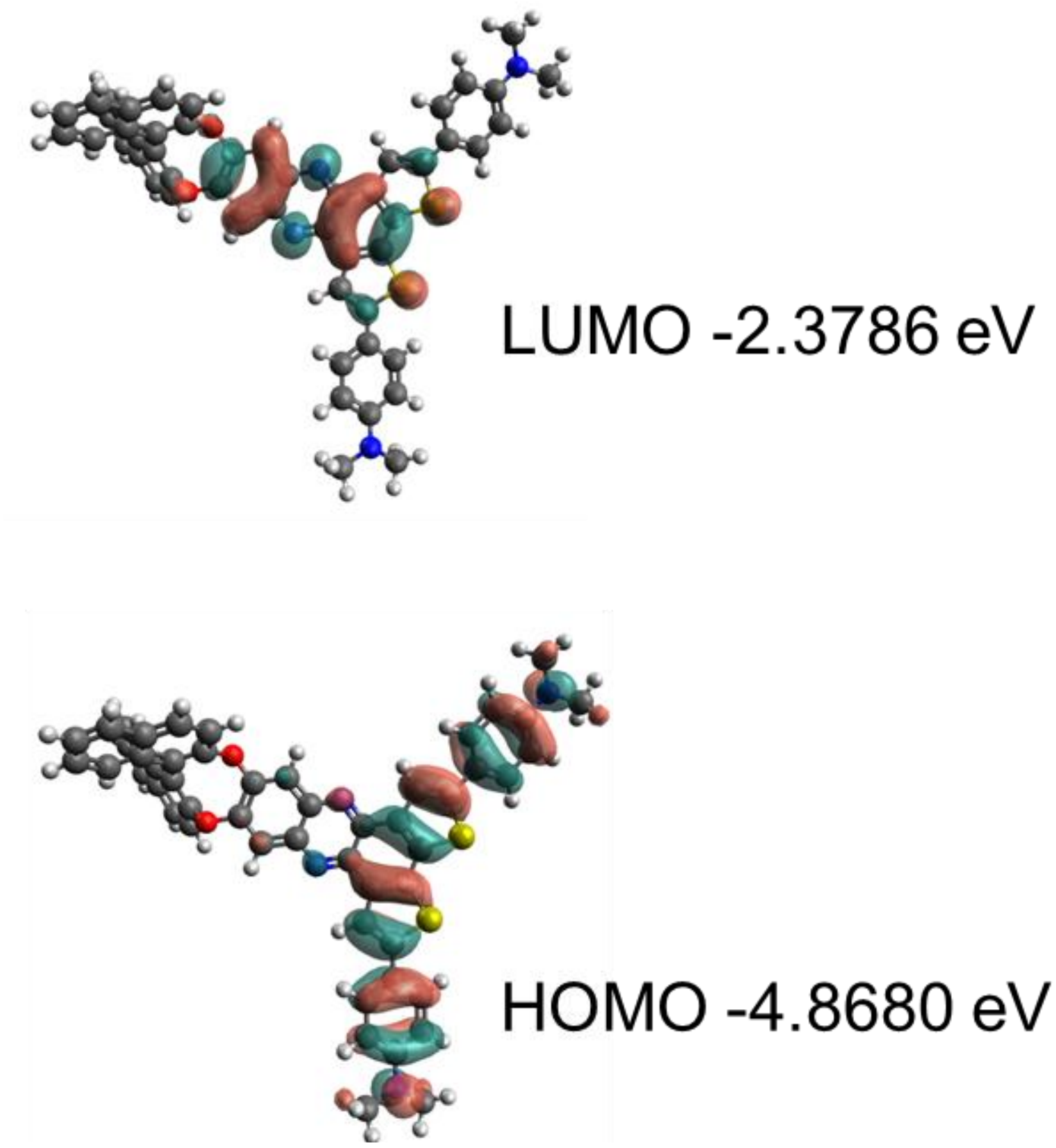

Figure 28. LUMO and HOMO values and orbital depictions for **DTP-dma**.

S7 – Ultraviolet-visible Spectroscopy

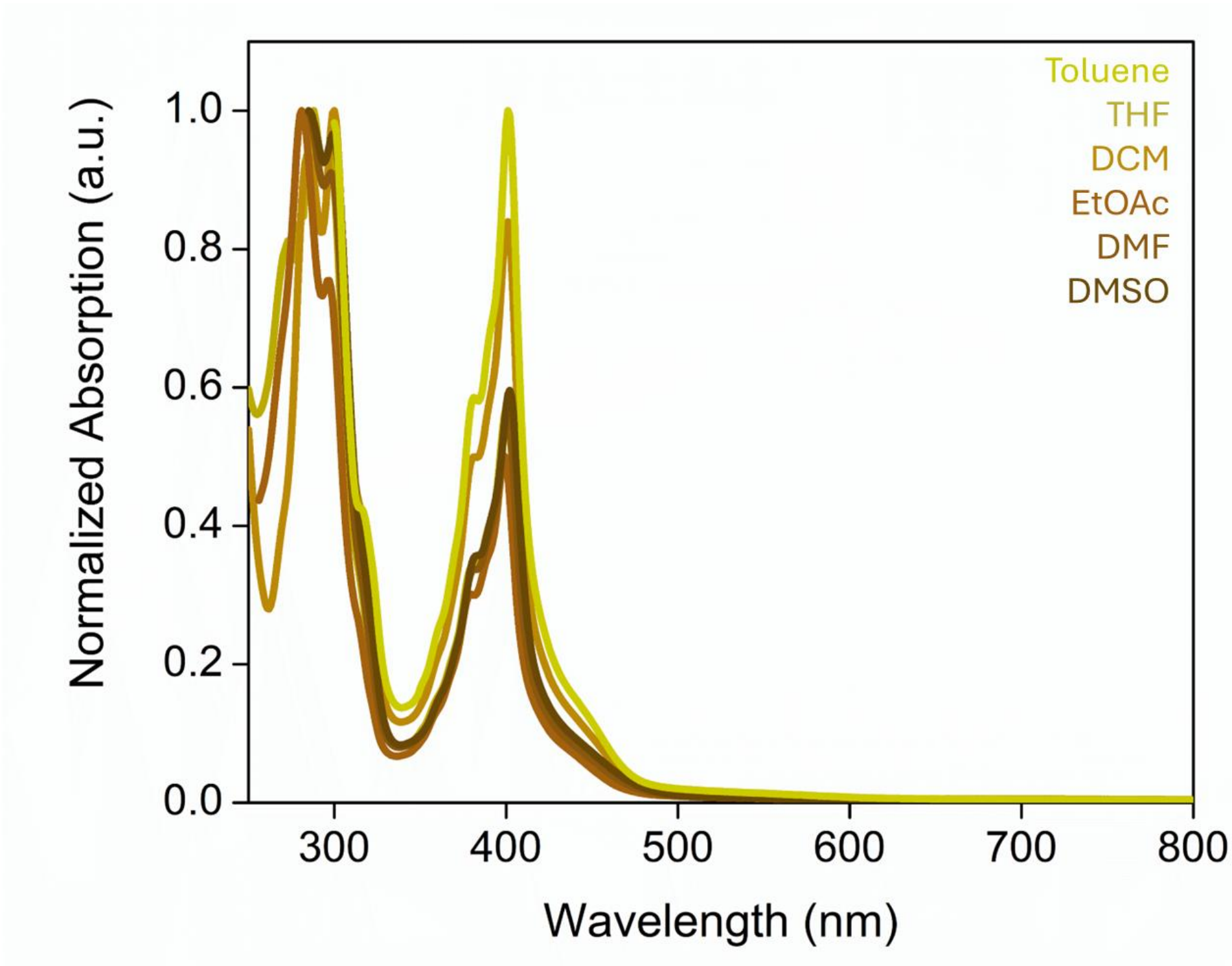

Figure 29. UV-Vis spectra of **DTP-Br** in different solvents.

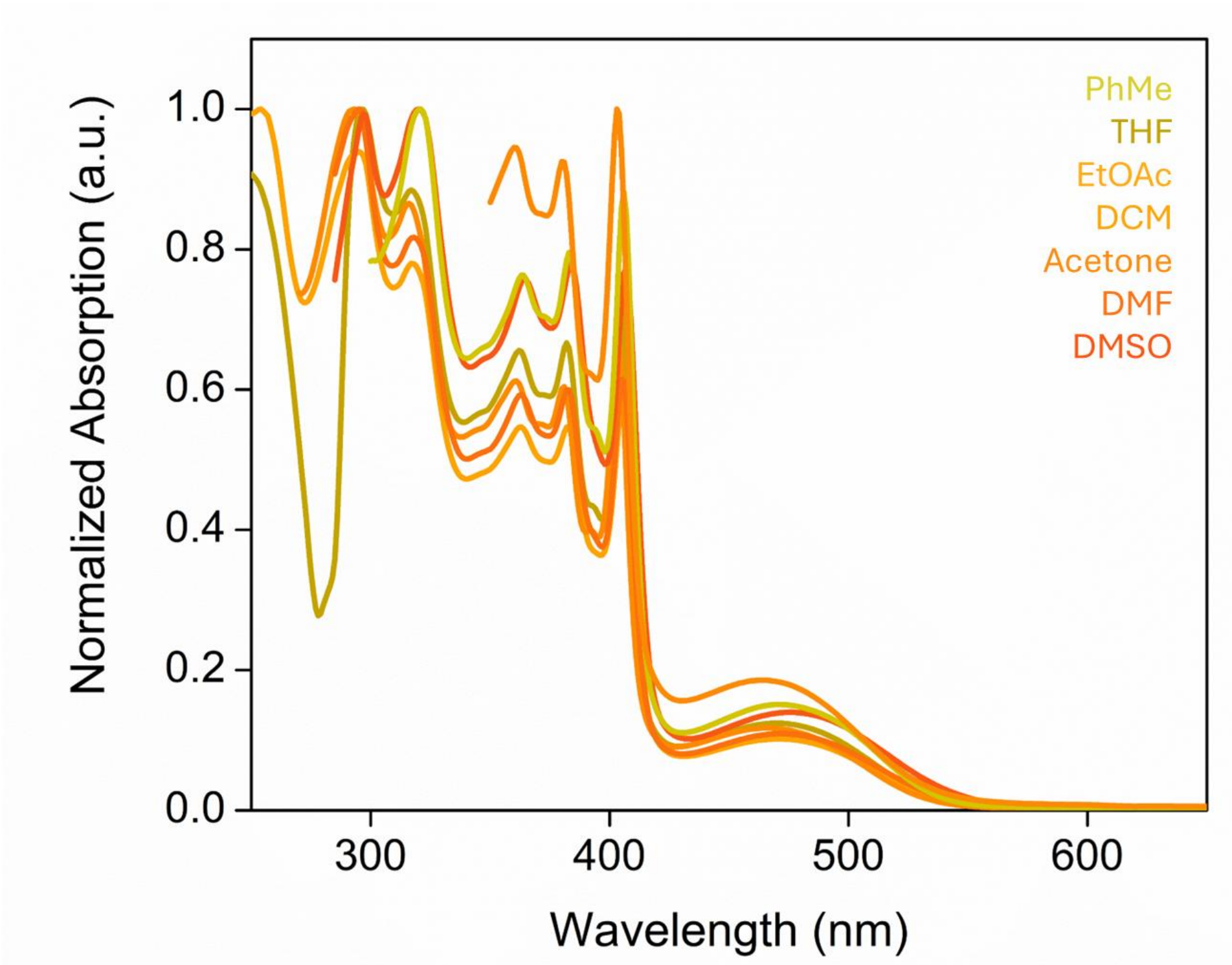

Figure 30. UV-Vis spectra of **DTP-dbf** in different solvents. Solvents listed in corresponding trace color.

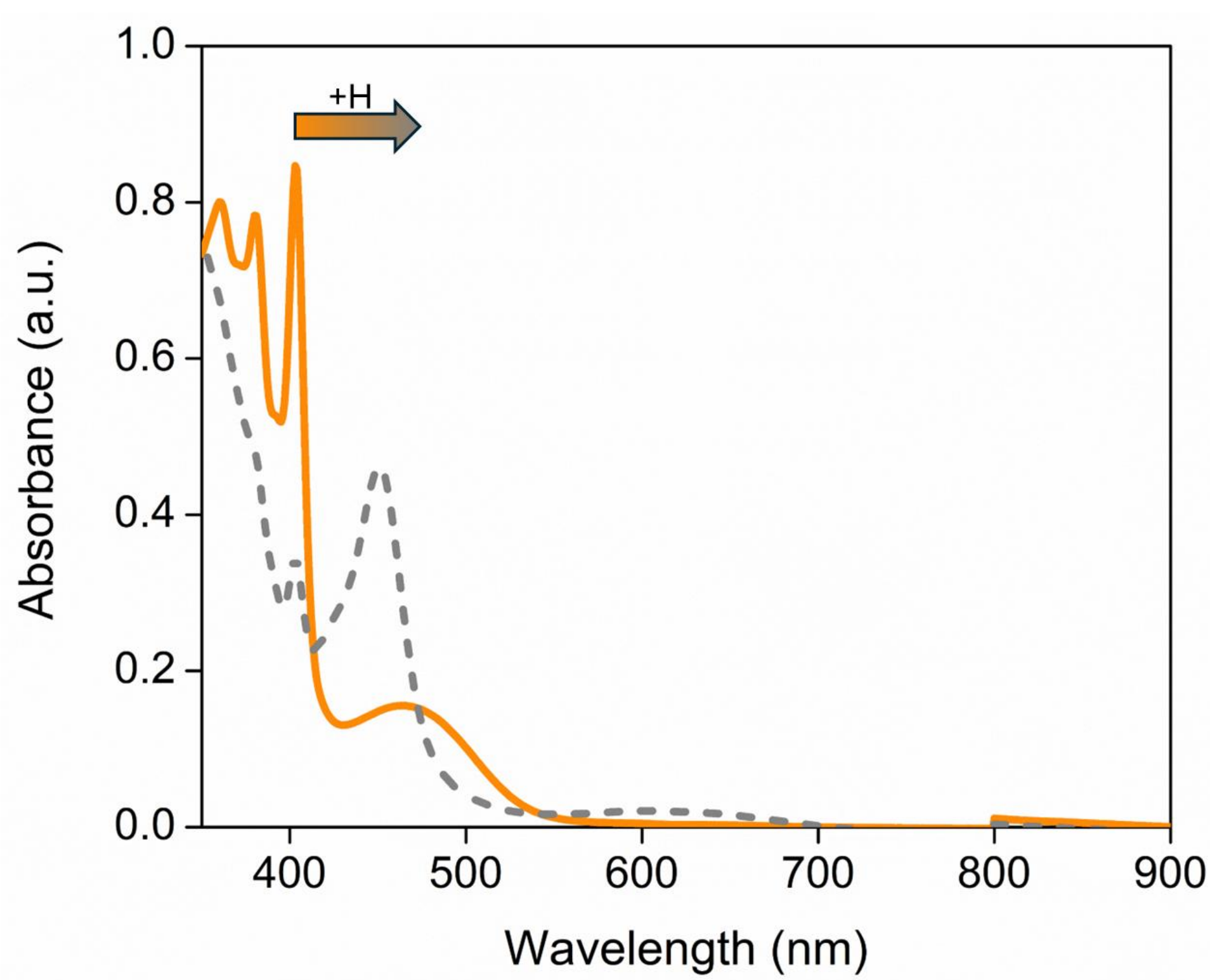

Figure 31. UV-Vis spectra of **DTP-dbf** in acetone as a neutral (orange) and protonated (grey) species. Solution was protonated using 2-3 drops of trifluoroacetic acid.

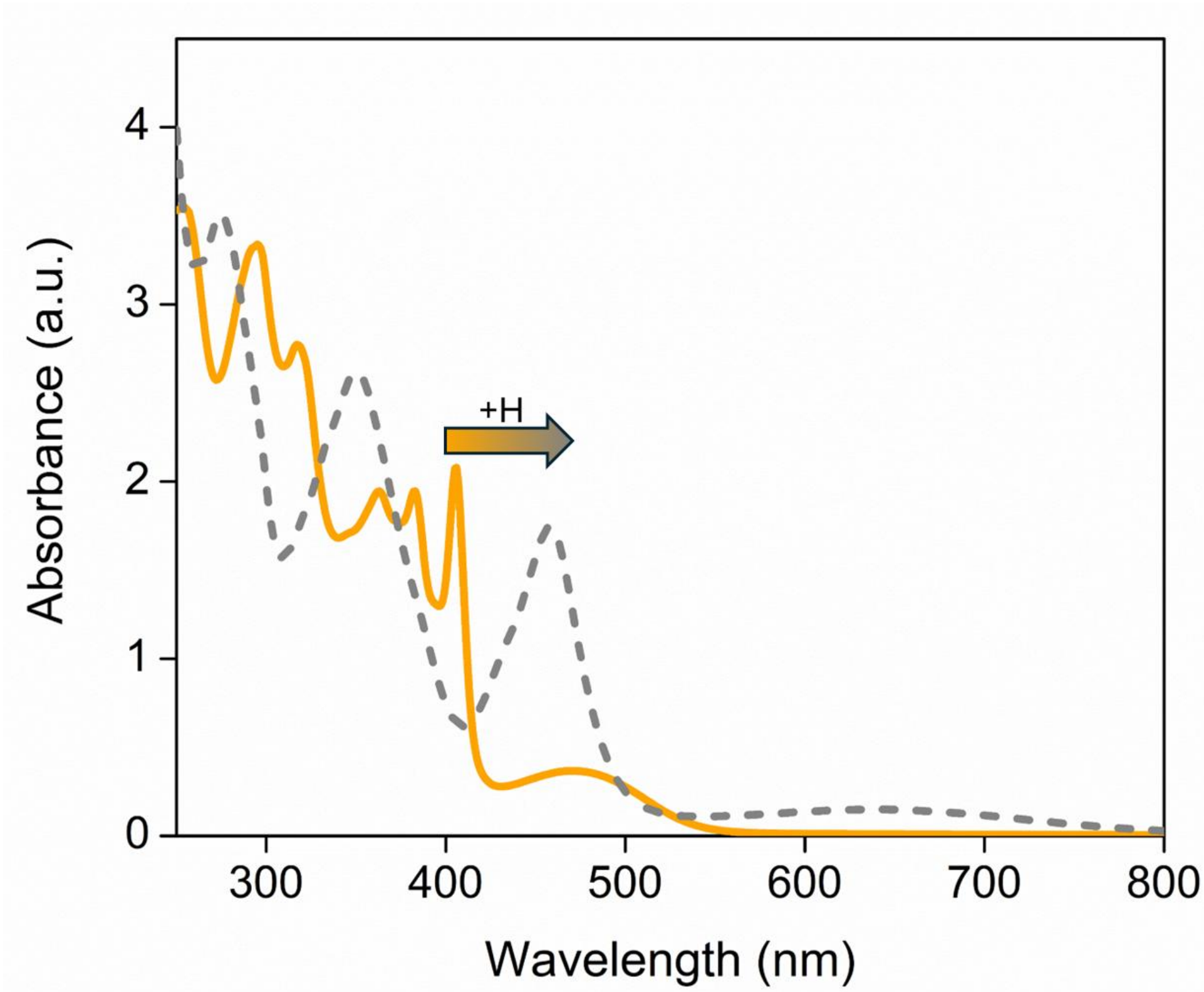

Figure 32. UV-Vis spectra of **DTP-dbf** in CH_2Cl_2 as a neutral (orange) and protonated (grey) species. Solution was protonated using 2-3 drops of trifluoroacetic acid.

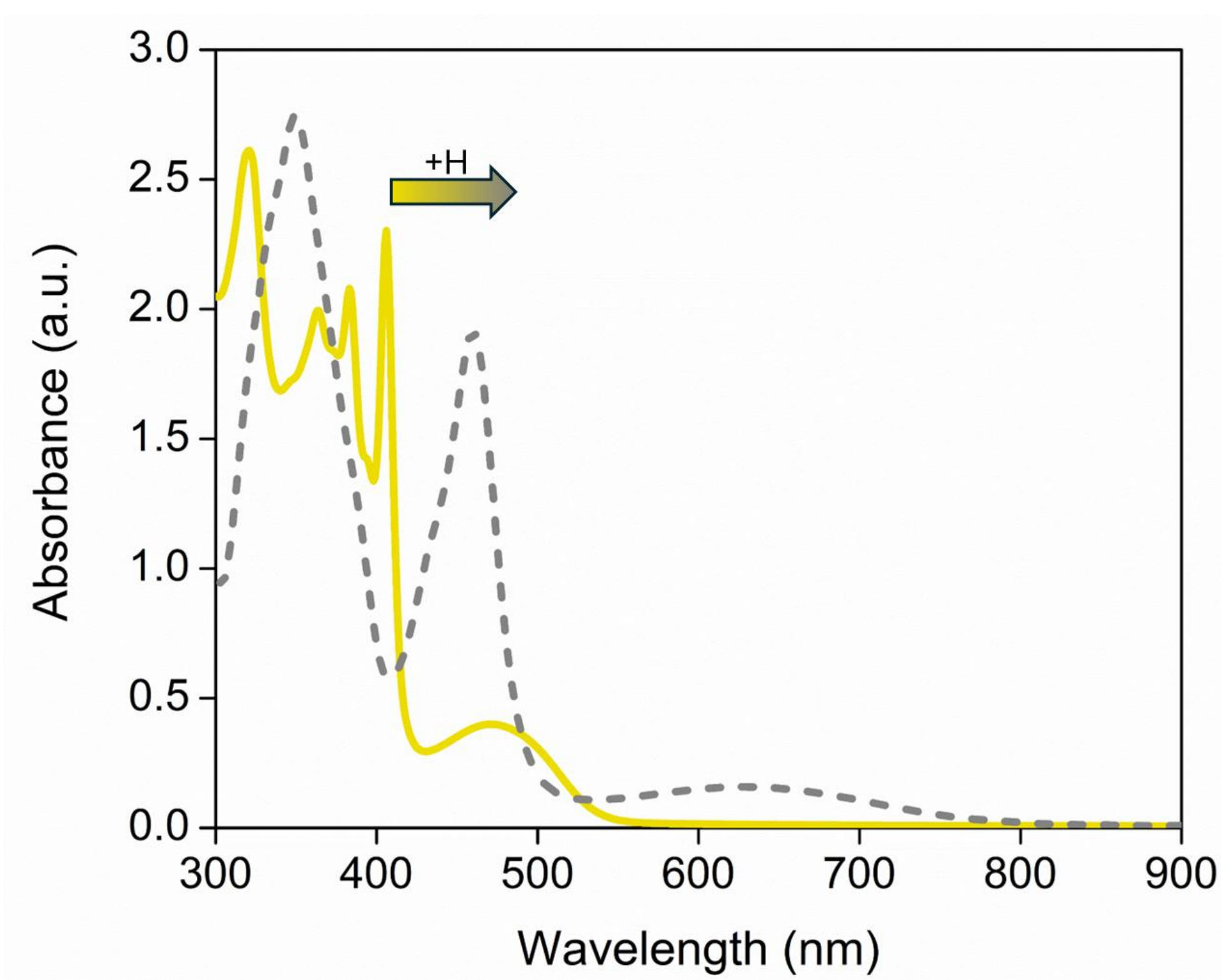

Figure 33. UV-Vis spectra of **DTP-dbf** in toluene as a neutral (yellow) and protonated (grey) species. Solution was protonated using 2-3 drops of trifluoroacetic acid.

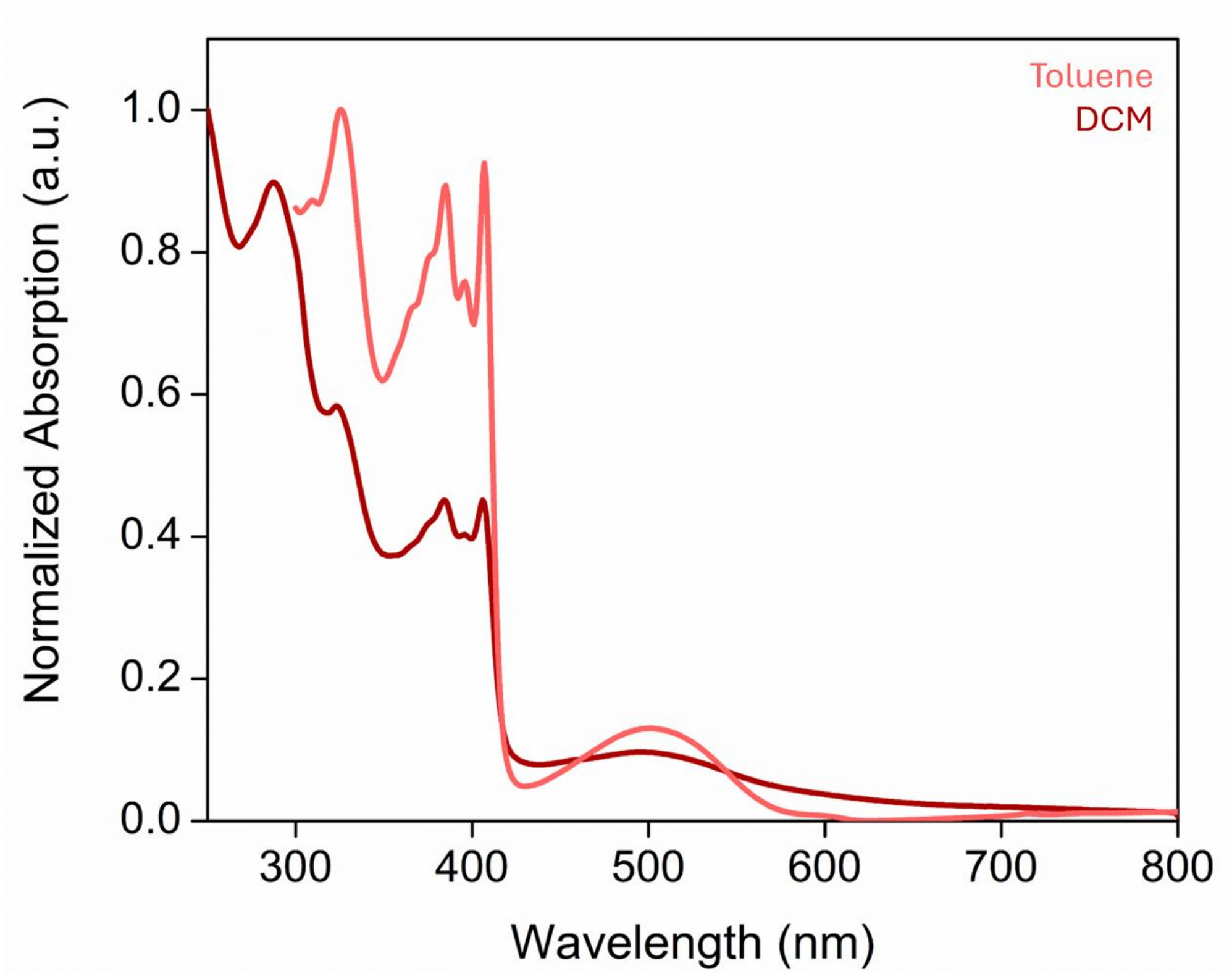

Figure 34. UV-Vis spectra of **DTP-cbz** in CH_2Cl_2 and toluene.

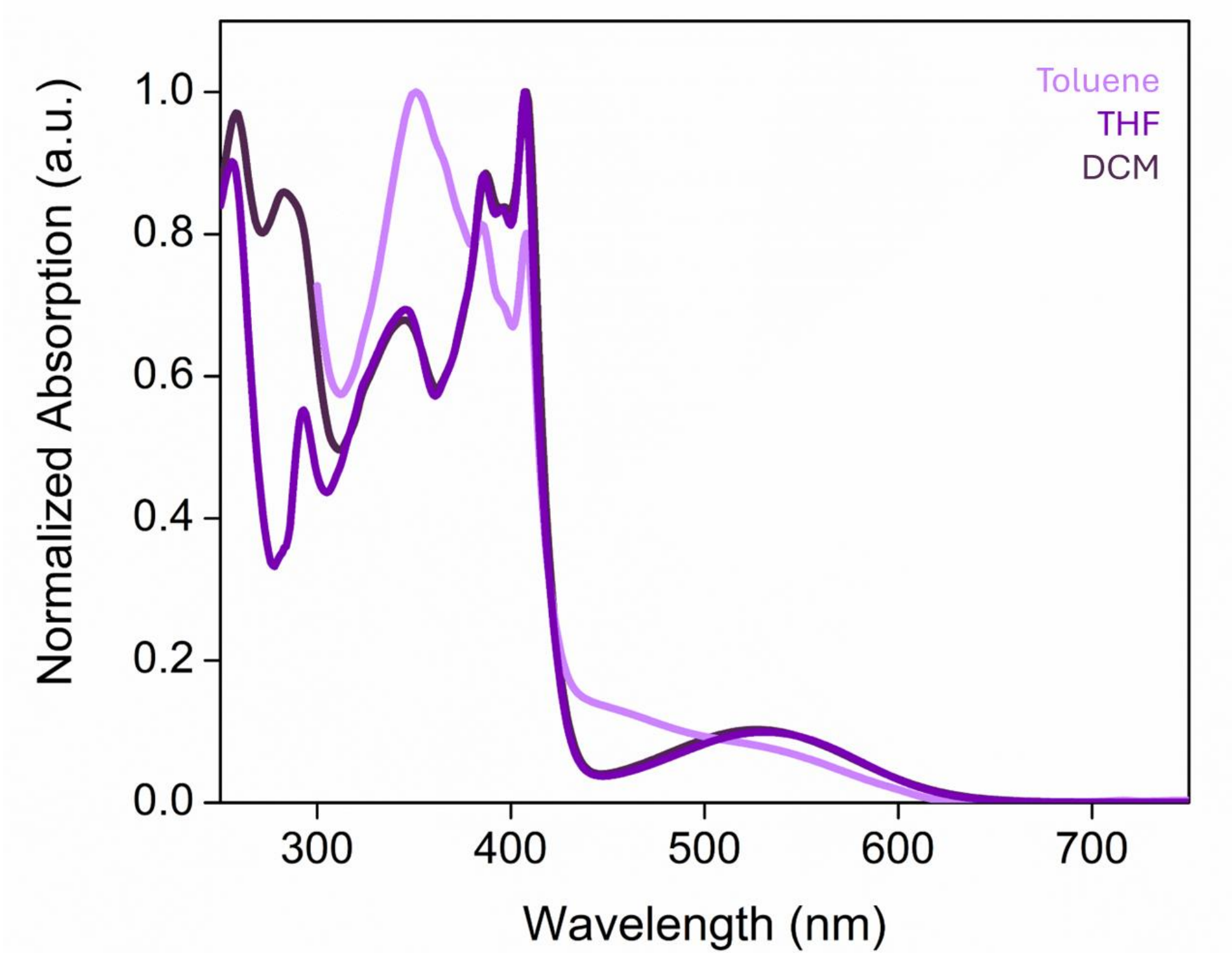

Figure 35. UV-Vis spectra of **DTP-dma** in CH_2Cl_2, THF, and toluene.

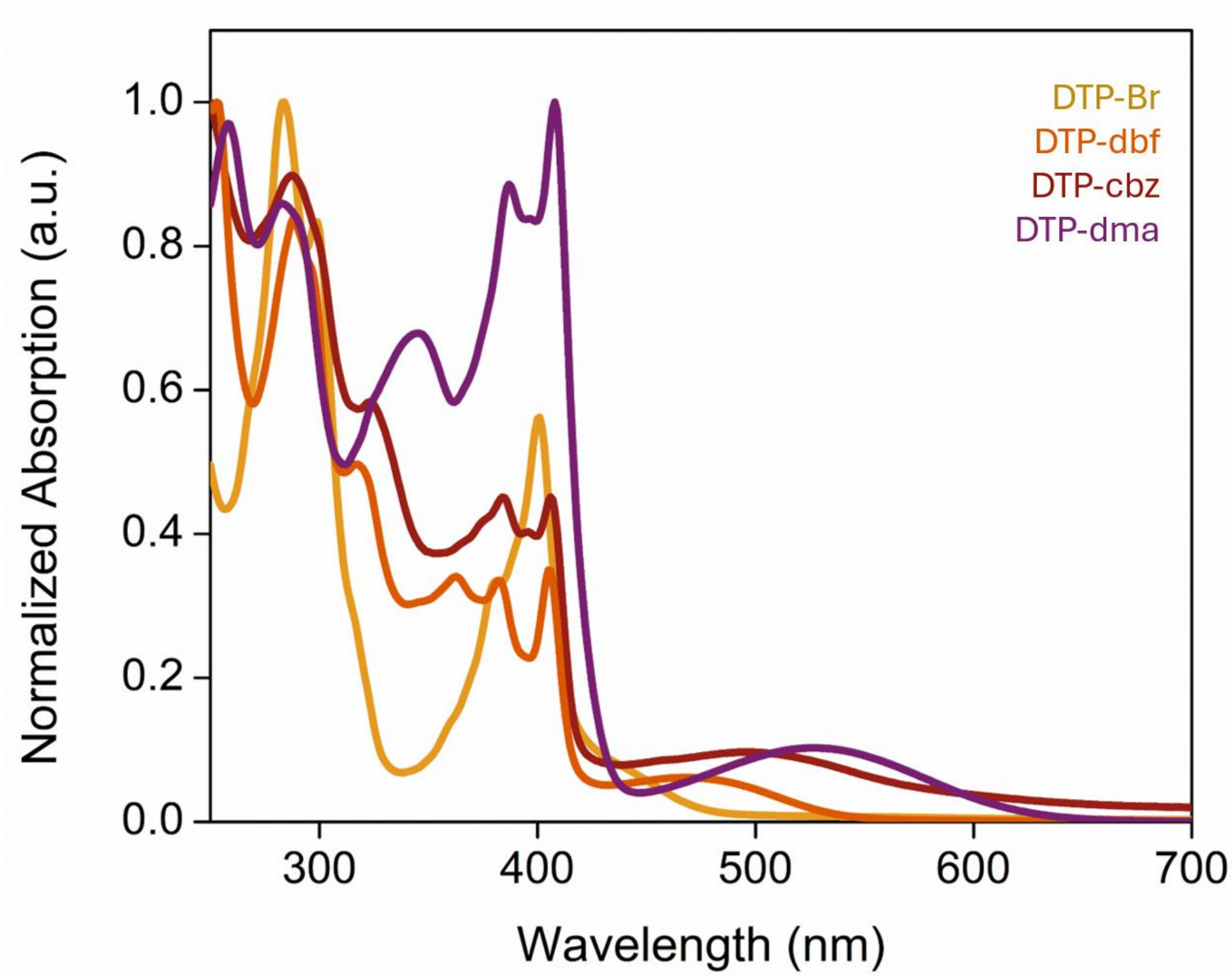

Figure 36. Normalized UV-Vis spectra of all derivatives in CH_2Cl_2.

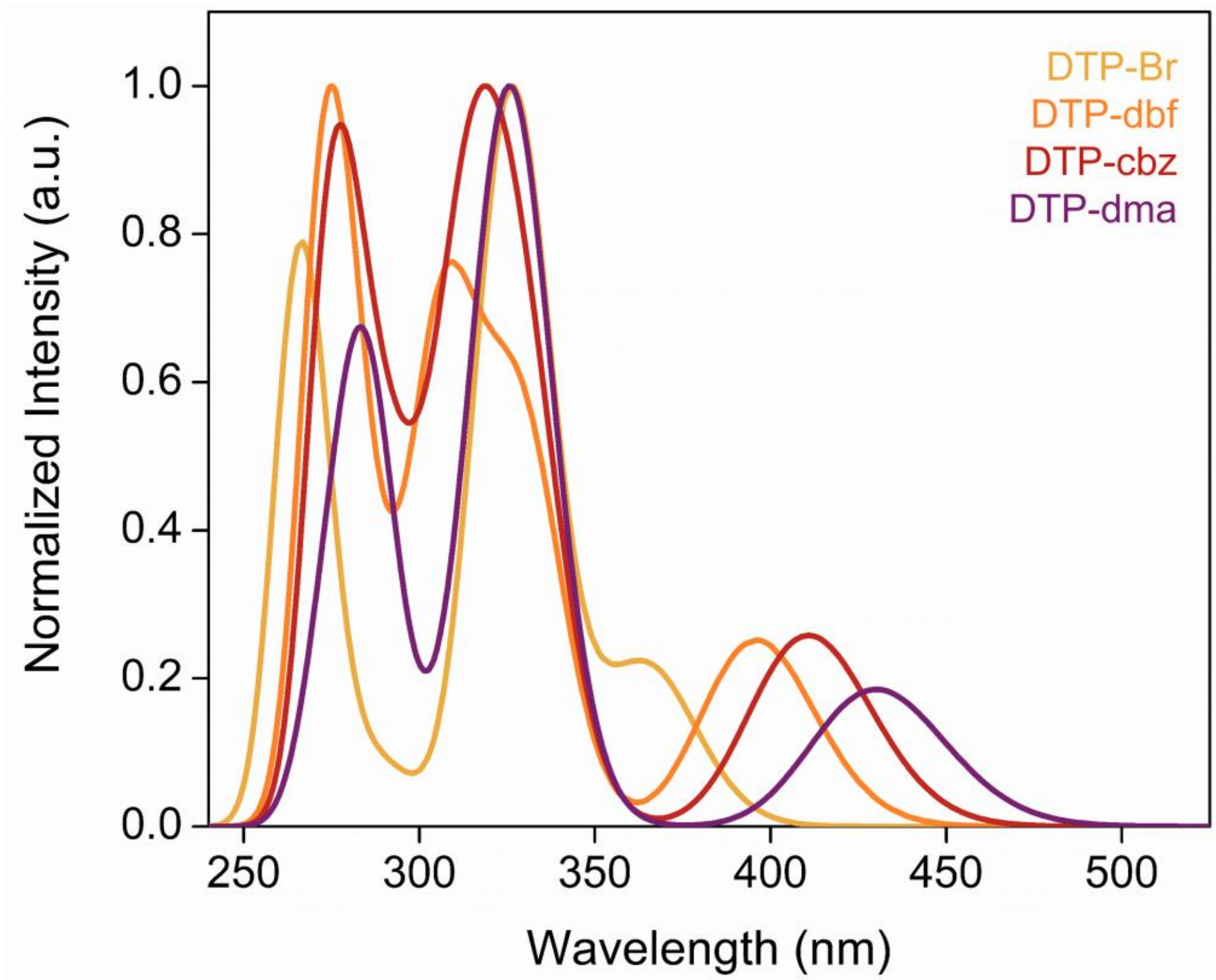

Figure 37. DFT-calculated UV-Vis spectra of all derivatives.

S8 – Photoluminescence Spectroscopy

Quantum yield

Quantum yield was calculated using the following equation:

$$\Phi = \Phi_{ref} \times \frac{I_{sample}}{I_{ref}} \times \frac{Abs_{ref}}{Abs_{sample}} \times \frac{n^2_{sample}}{n^2_{ref}}$$

where Φ_{ref} is the quantum yield, *I* is the integrated intensity of emission spectra, *A* is the absorbance at the excitation wavelength, *n* is the refractive index of the solvent, and subscripts *ref* and *sample* correspond to the reference fluorophore and the measured sample, respectively. 9,10-diphenylanthracene was used as the reference fluorophore.

Lippert-Mataga Plot

Lippert-Mataga plots were calculated using the following equations:

For the x-axis (orientation polarizability, ΔF):

$$\Delta F = \frac{(\varepsilon_r - 1)}{(2\varepsilon_r + 1)} - \frac{(n^2 - 1)}{(2n^2 + 1)}$$

where ε_r is the dielectric solvent and n is the refractive index of the solvent. Here, F(ε_r, n) ranges from 0.018376 for toluene and 0.27438 for DMF.

The y-axis is calculated by determining the stokes shift, $\Delta\upsilon$:

$\Delta\upsilon = \upsilon_{abs} - \upsilon_{em}$.

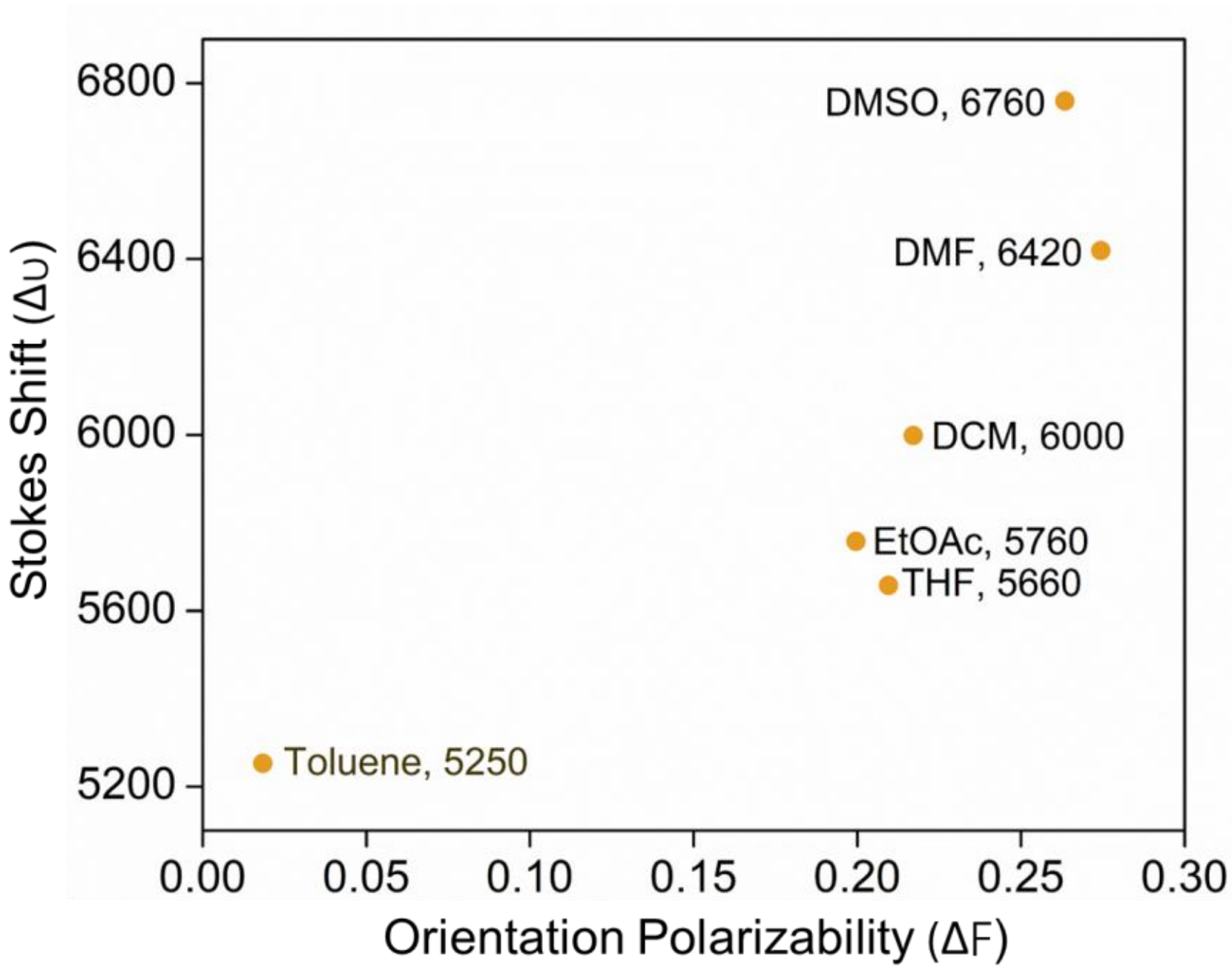

Figure 38. Lippert-Mataga plot for **DTP-Br**.

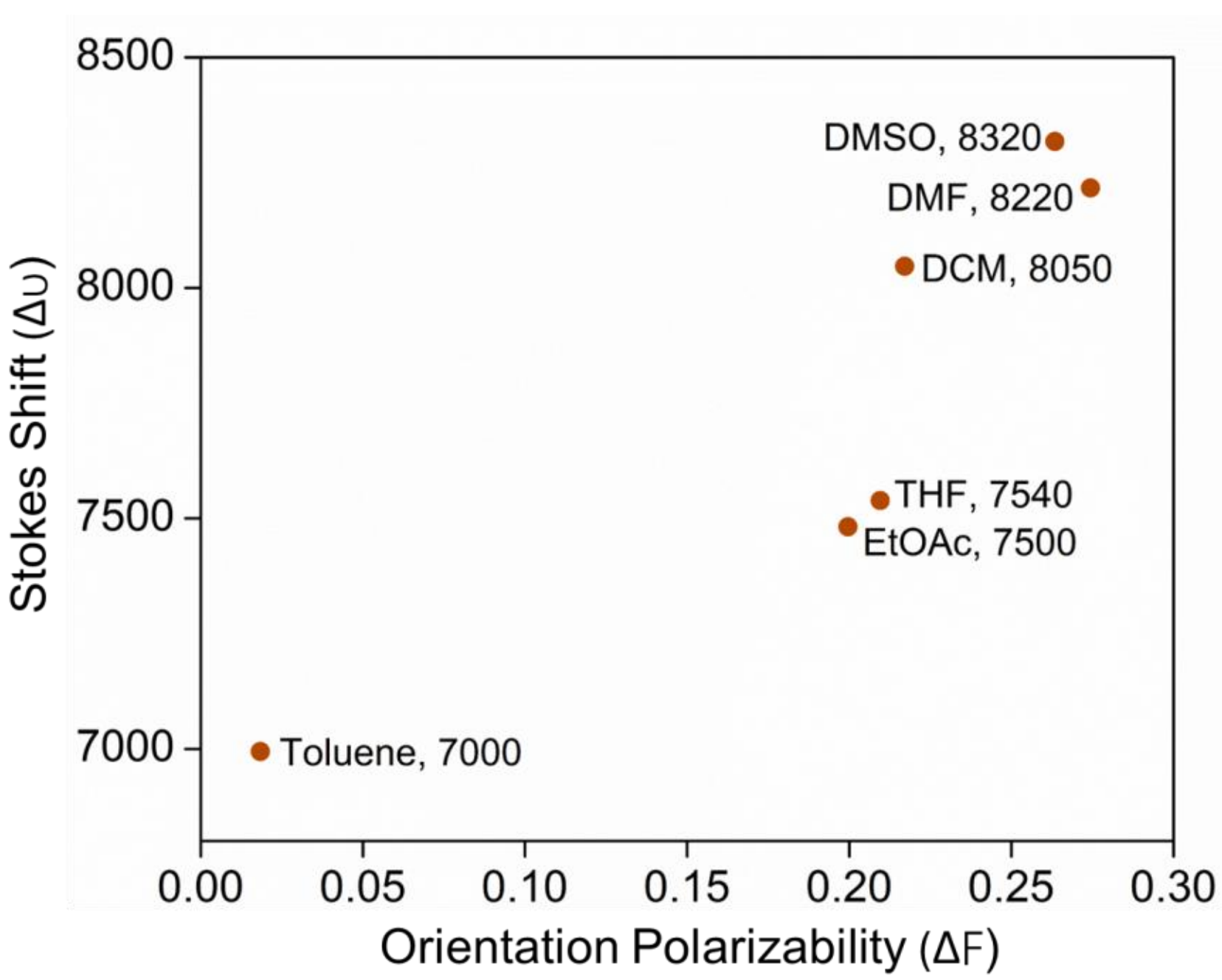

Figure 39. Lippert-Mataga plot for **DTP-dbf**.

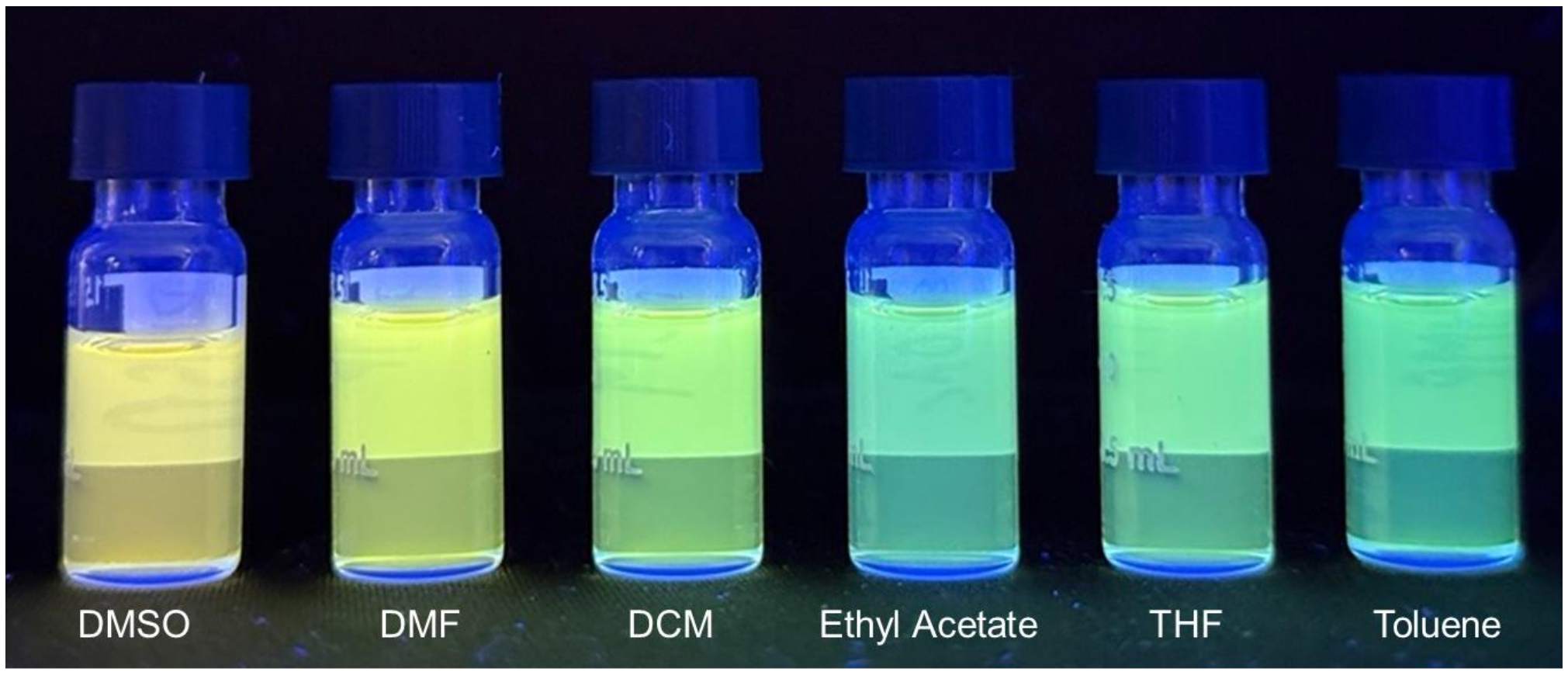

Figure 40. Photo of **DTP-Br** in different solvents. Samples irradiated at 365 nm.

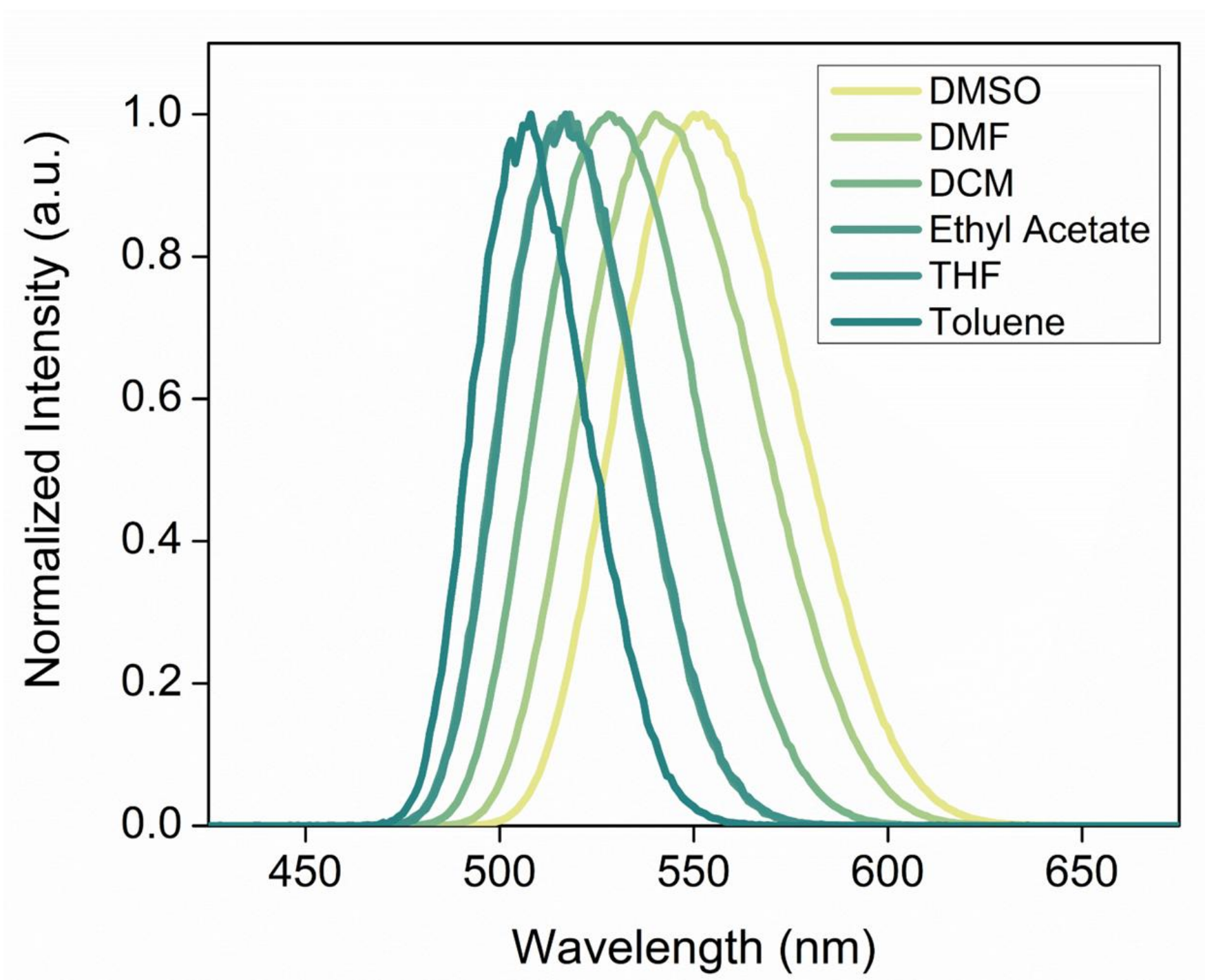

Figure 41. Photoluminescence of **DTP-Br** in different solvents. Excitation at 365 nm.

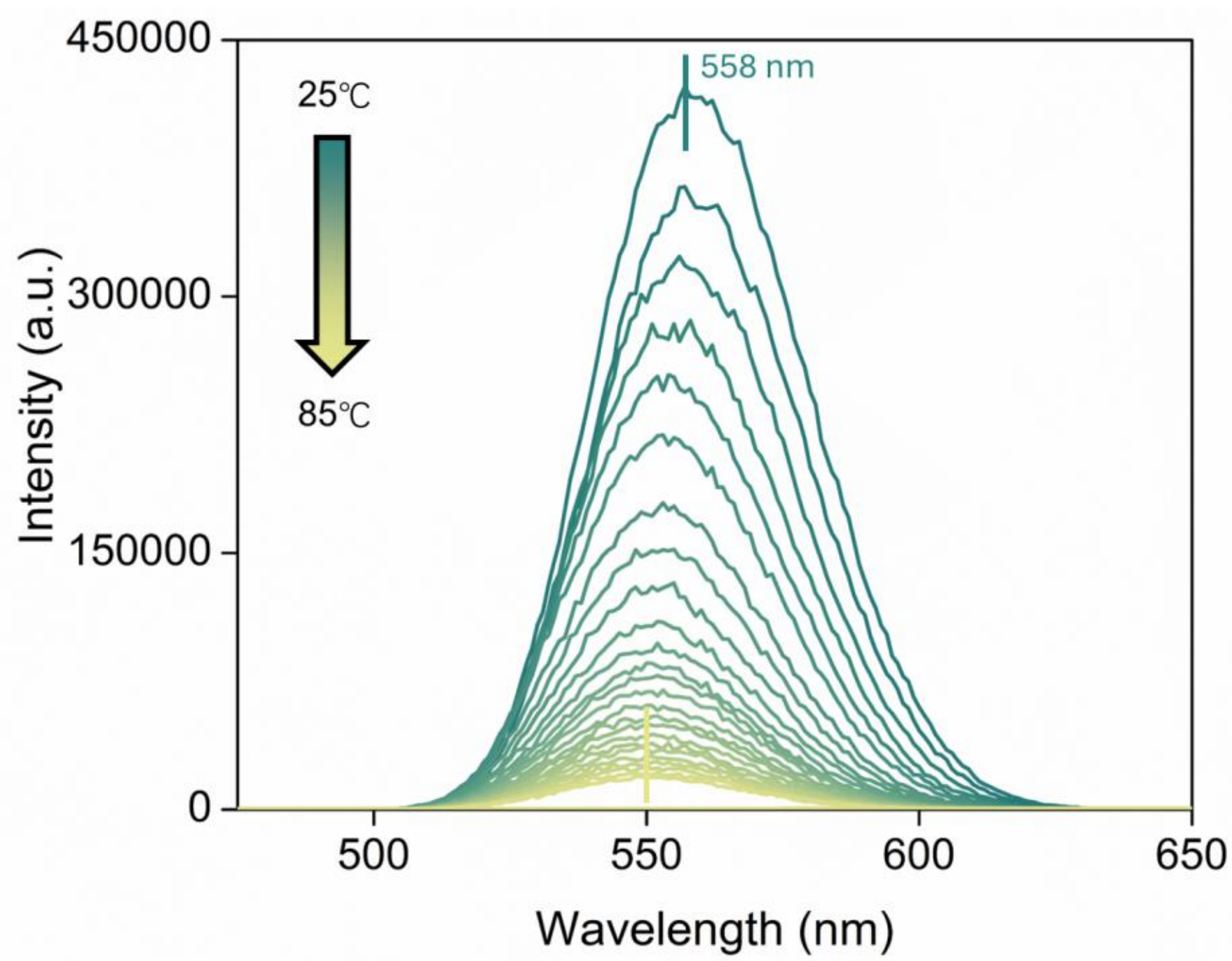

Figure 42. Temperature-dependent photoluminescence of **DTP-Br** in DMSO.

Procedure: A solution of **DTP-Br** in DMSO at 10 μM was prepared. Fluorimeter parameters were set to measure 49 times. Scans began at room temperature (~25 ℃) and temperature controller was started after the second measurement. Temperature controller warmed cuvette cell up to 85℃.

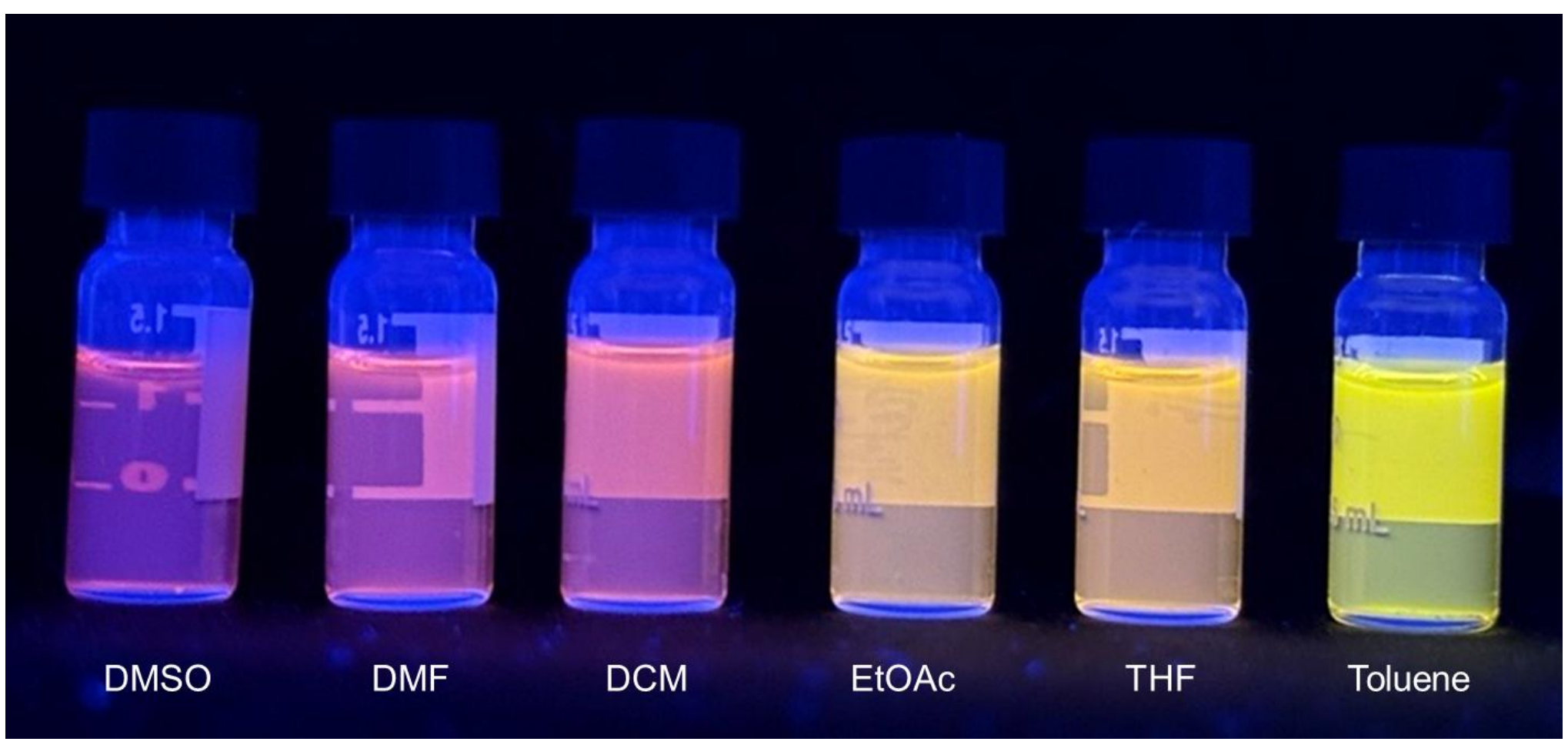

Figure 43. Photo of **DTP-dbf** in different solvents.

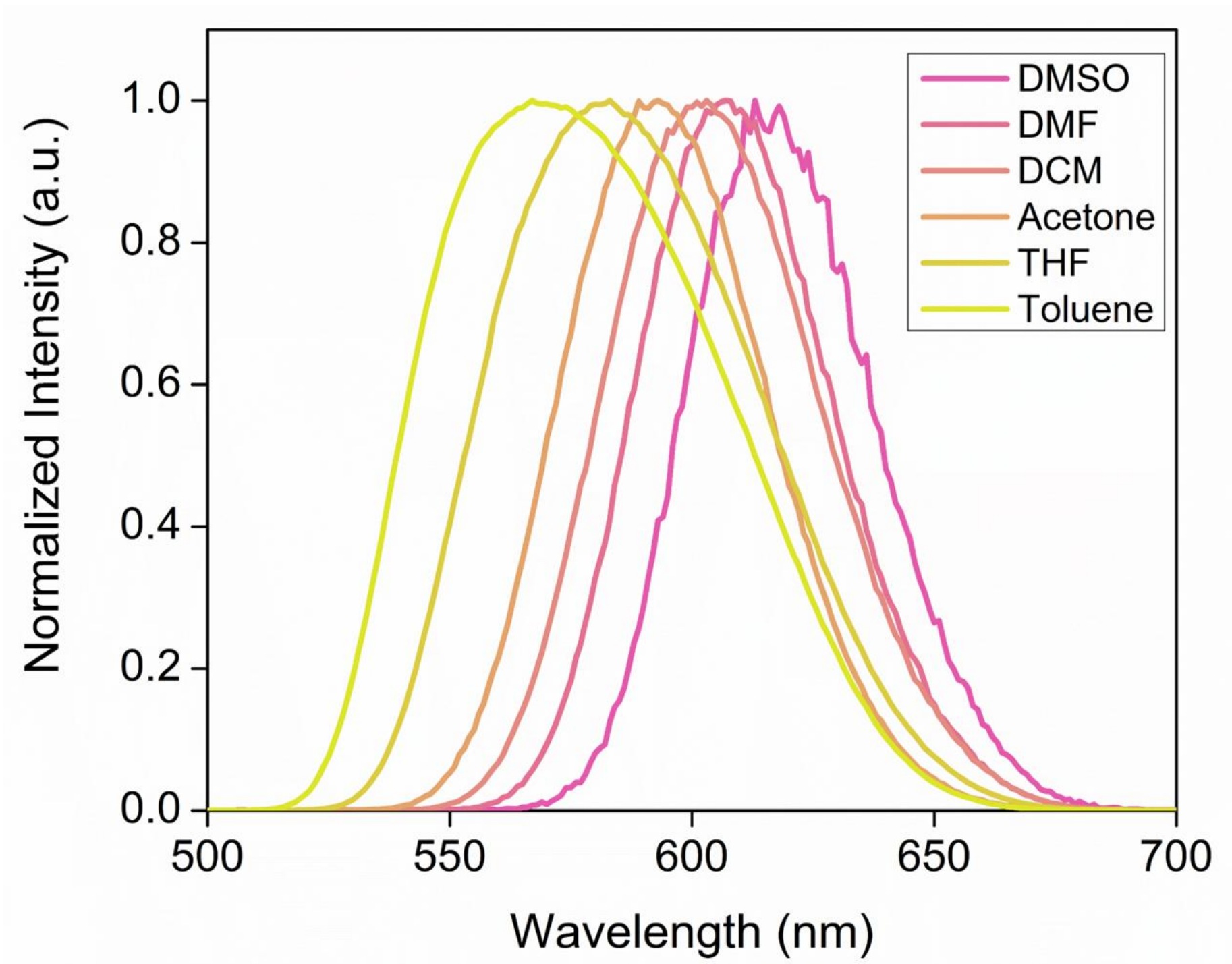

Figure 44. Photoluminescence of **DTP-dbf** in different solvents. Excitation at 365 nm.

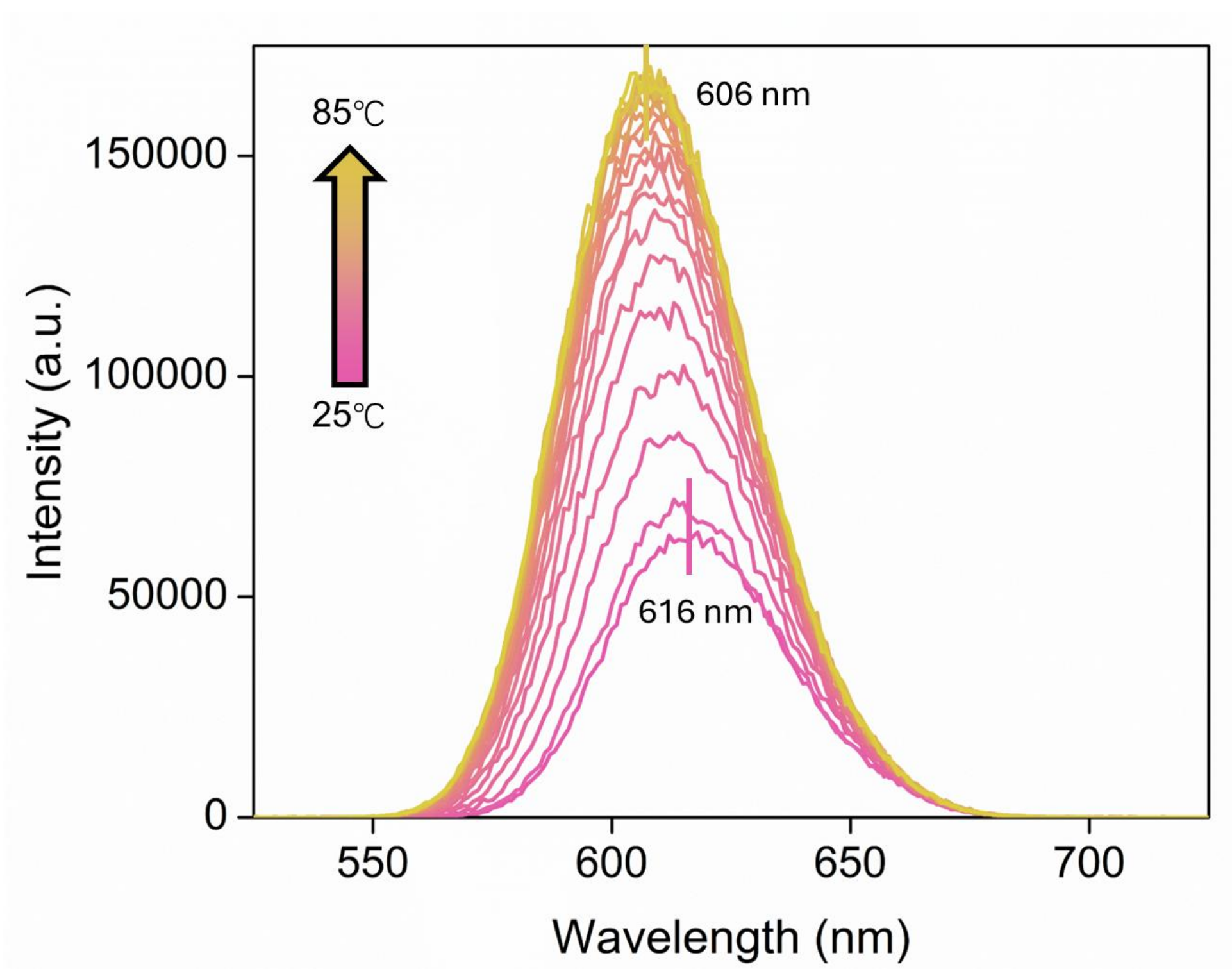

Figure 45. Temperature-dependent photoluminescence of **DTP-dbf** in DMSO.

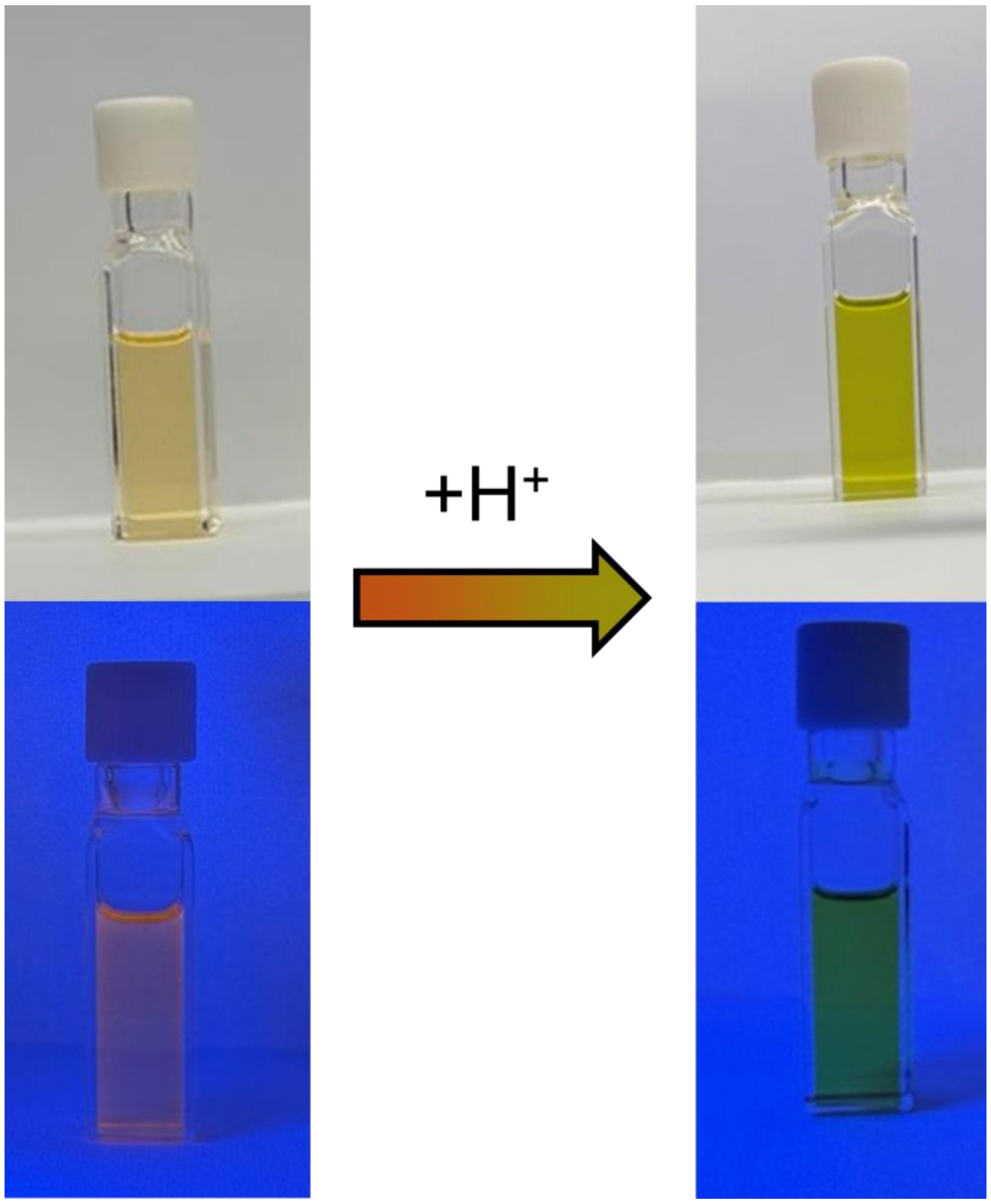

Figure 46. Neutral **DTP-dbf** (left) and protonated (right) in daylight (top) and under 365 nm irradiation (bottom).

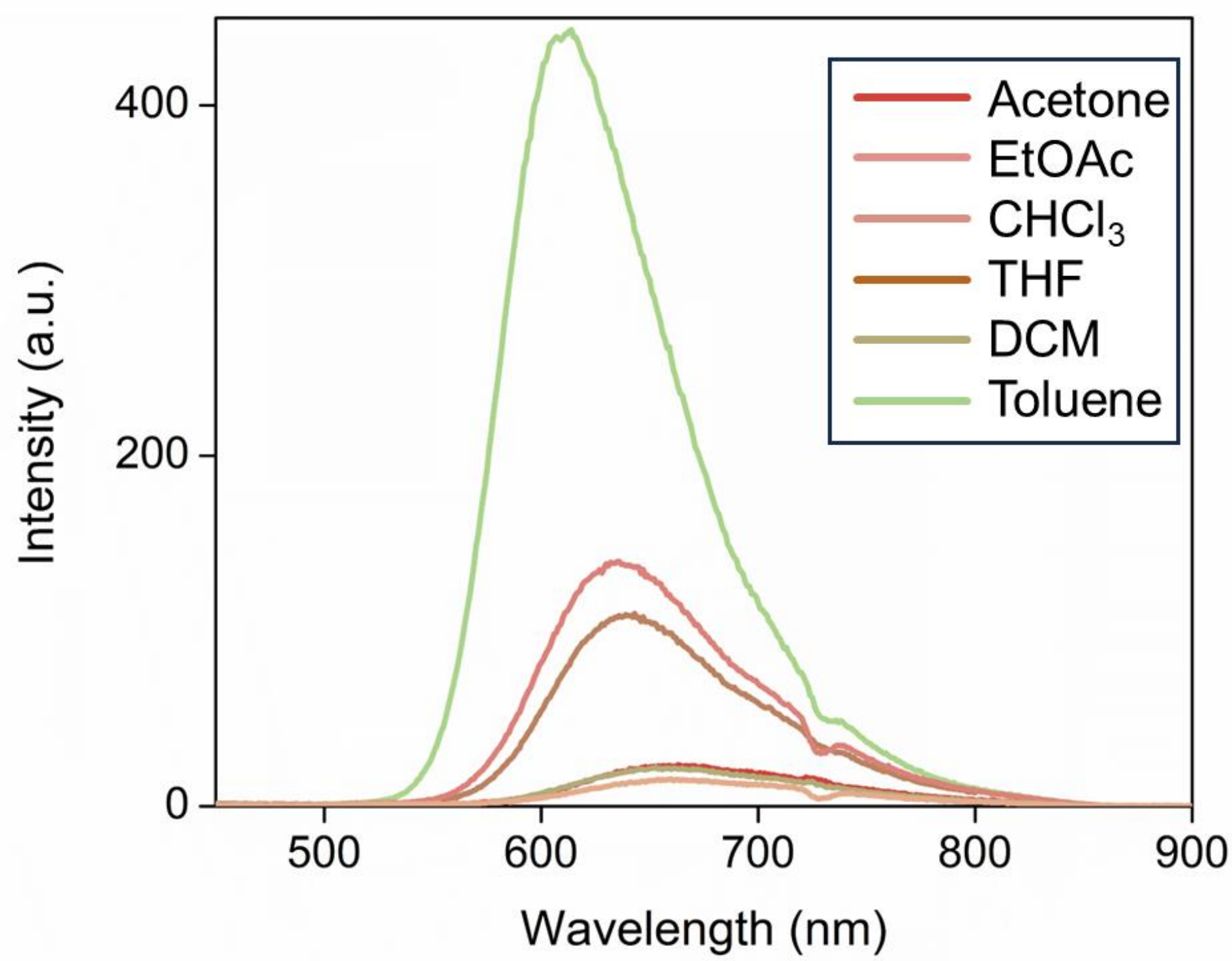

Figure 47. Photoluminescence of **DTP-cbz** in different solvents. Excitation at 365 nm.

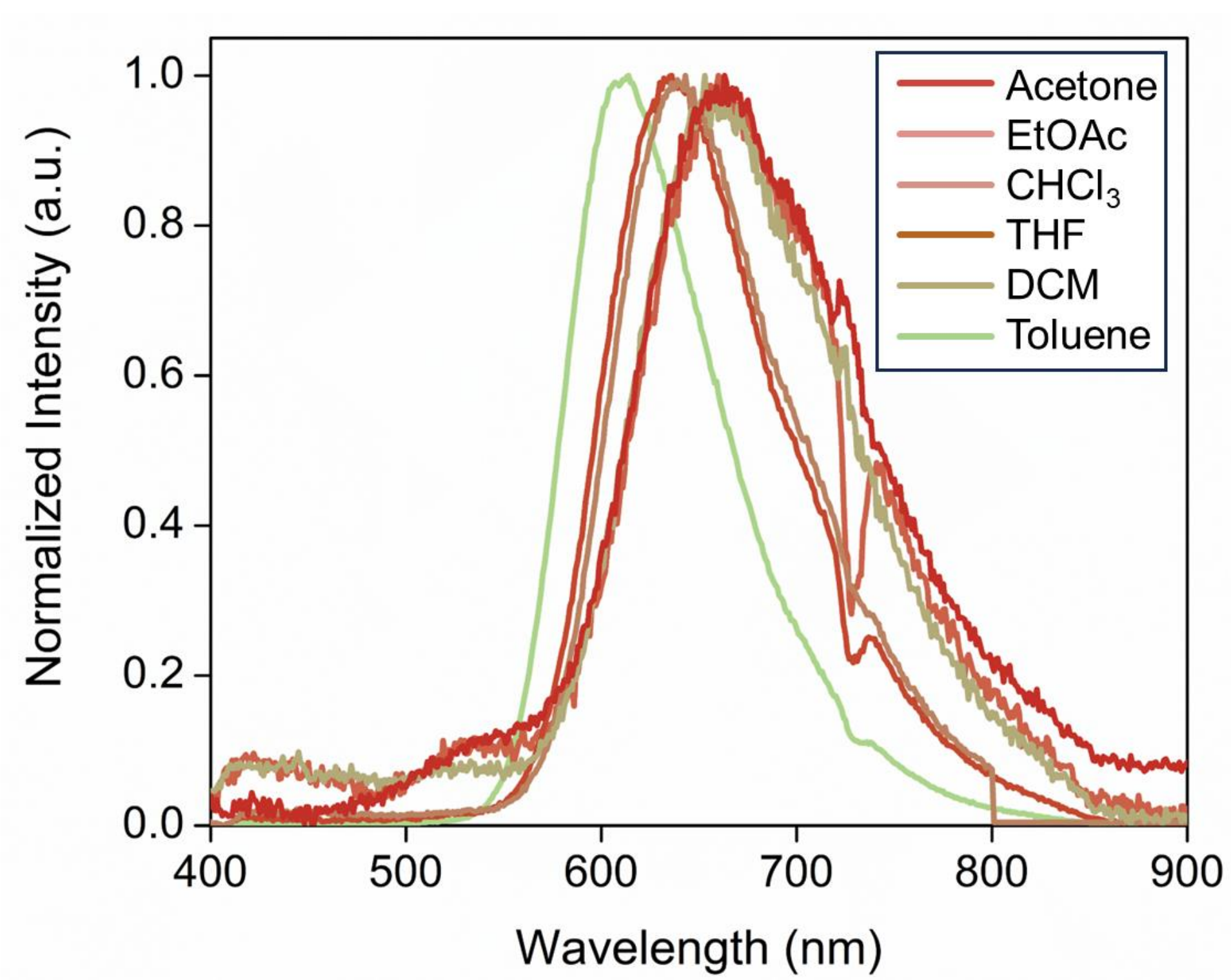

Figure 48. Normalized photoluminescence of **DTP-cbz** in different solvents. Excitation at 365 nm.

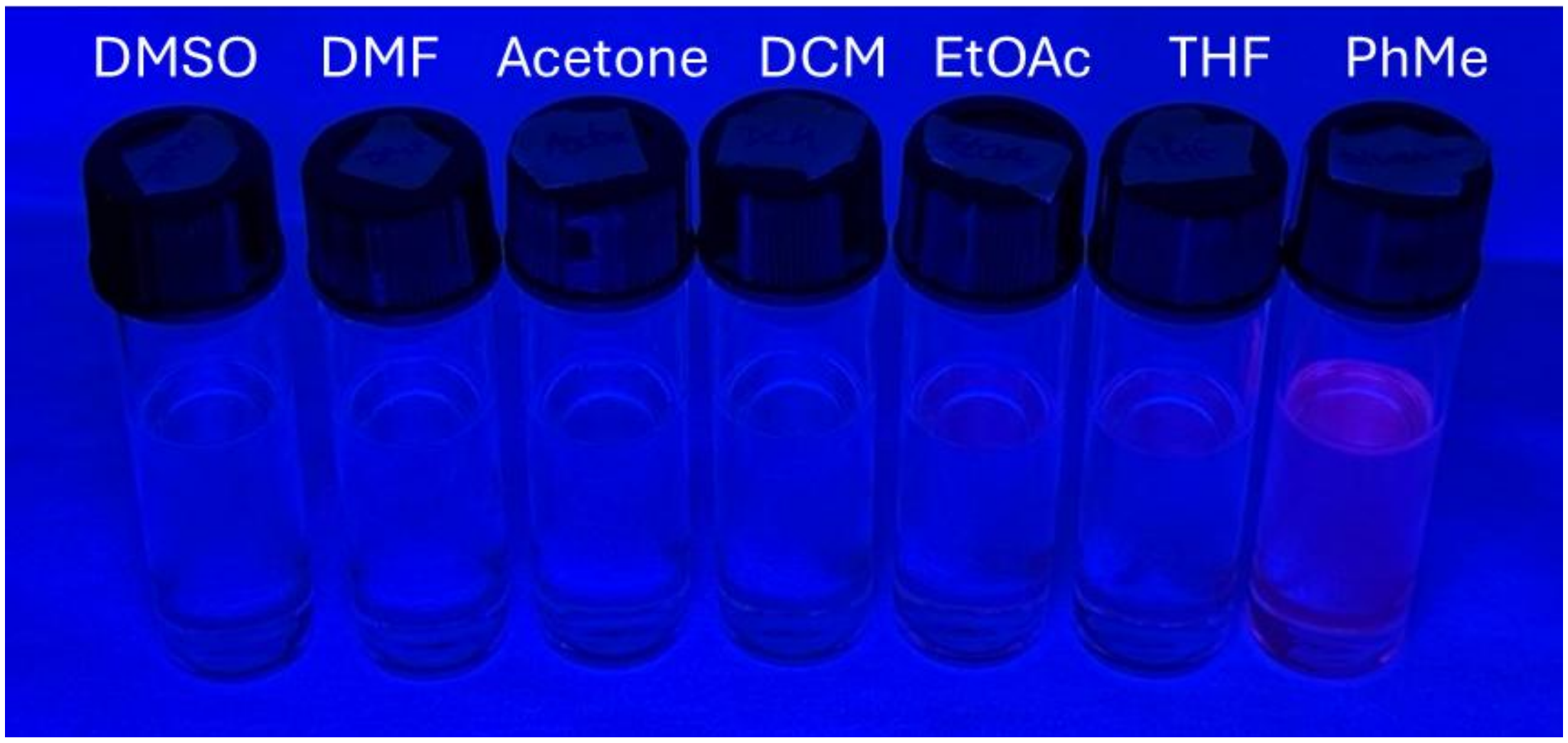

Figure 49. Photo of **DTP-cbz** in different solvents.

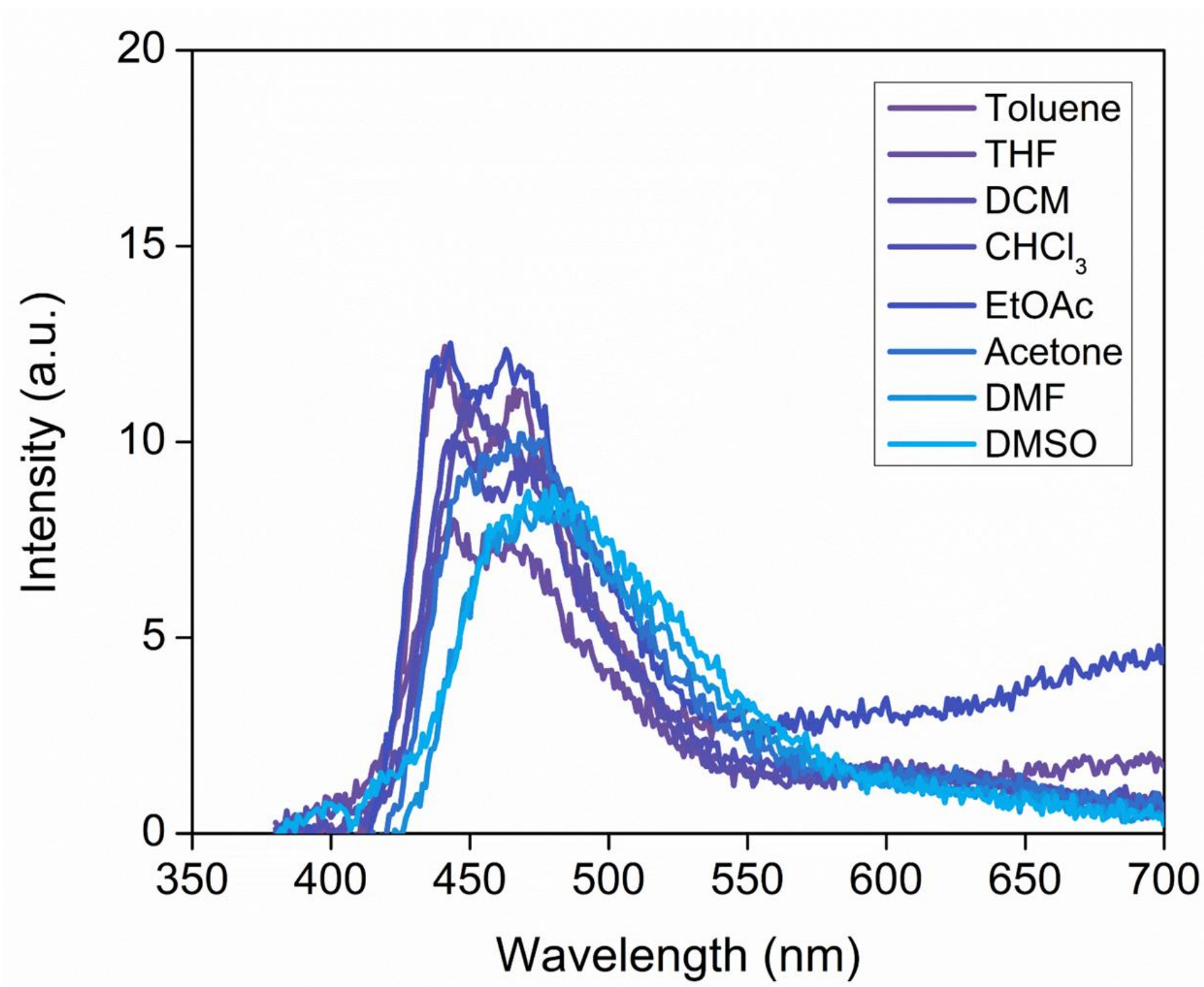

Figure 50. Photoluminescence of **DTP-dma** in different solvents. Excitation at 365 nm.

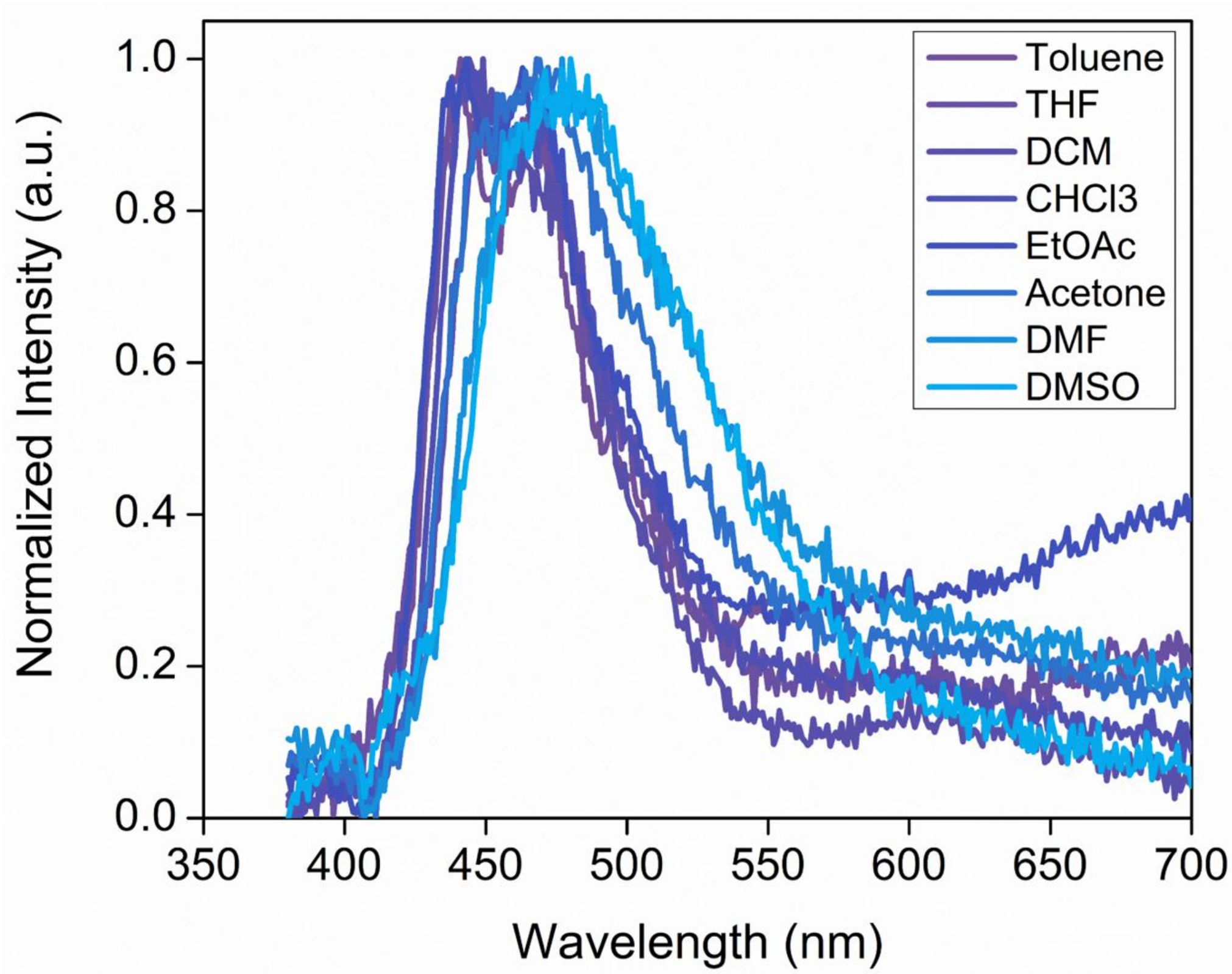

Figure 51. Normalized photoluminescence of **DTP-dma** in different solvents. Excitation at 365 nm.

To demonstrate the versatility of these chromophores in an applied setting, we tested the photoluminescence intensity output as a function of circularly polarized light input.

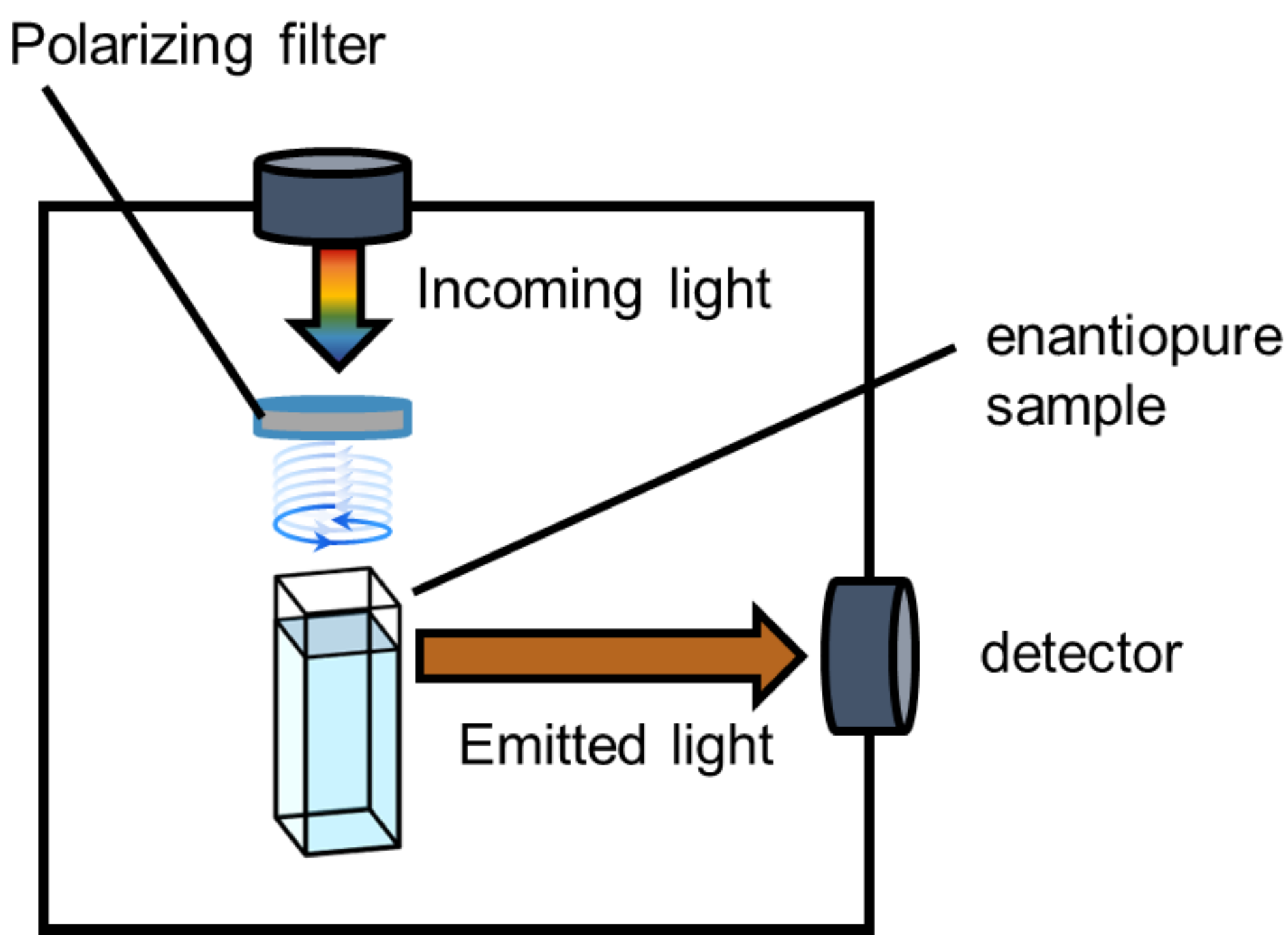

Figure 52. Fluorometer setup for differential emission experiment.

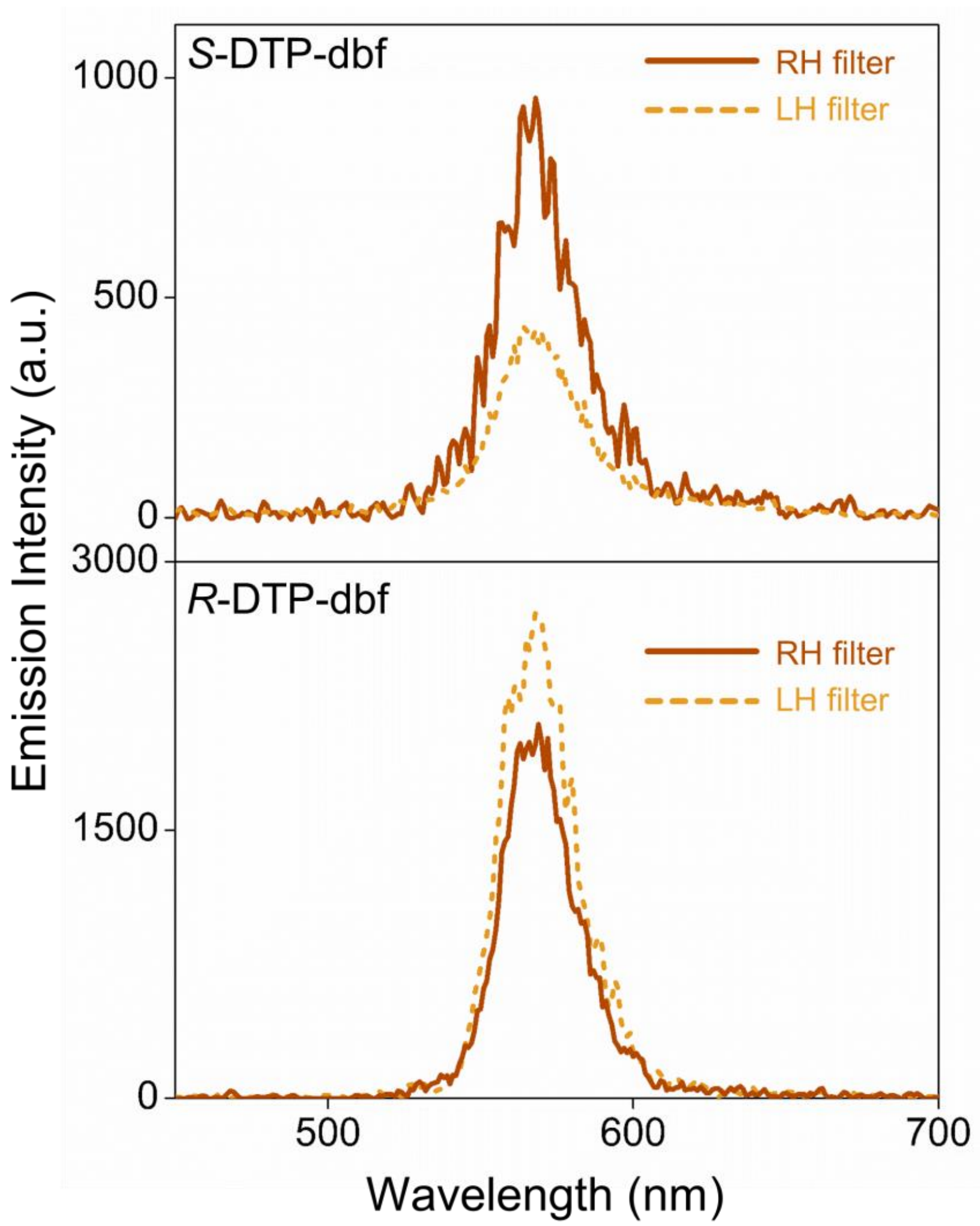

Figure 53. Comparison of **DTP-dbf** enantiomers emission intensity in response to LH and RH light.

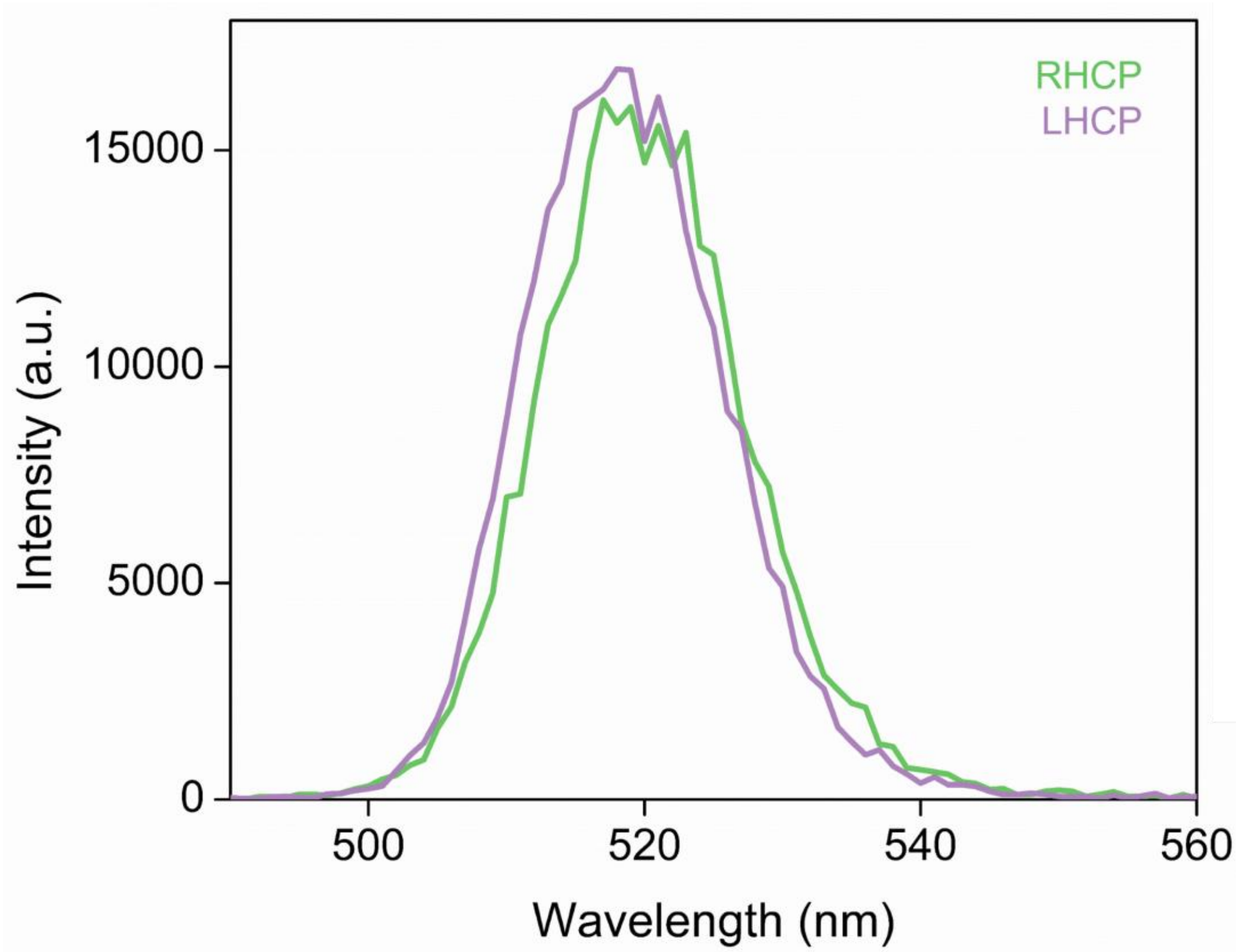

Figure 54. Rhodamine 6G in ethanol. λ_{exc}=480 nm.

Procedure for data collection.
One of two polarization filters (Edmund Optics), either left-handed or right-handed were placed in the fluorimeter sample cell between the incoming light source and the sample. The lights to the lab were off and it was dark throughout the duration of the experiment to prevent stray light from entering the detector. The enantiopure sample was excited and intensity was recorded under one set of filter conditions. The opposite filter was used, and the same enantiomer was excited and intensity was once again recorded. This process was repeated for the opposite enantiomer. Raw data was collected using a 0.1 s integration, but the measured data was smoothed to average data collection to every 0.5 s.

Rhodamine 6G was used as a control for this experiment. No difference in emission was observed for the achiral control.

S9 – Film Images

Film development
4.5 g of commercial PMMA was dissolved in 90 mL of CH_2Cl_2 to make the PMMA solution. To make 1 sample, 1-2 mg of compound is placed in a vial. An appropriate amount of the PMMA solution is added to achieve a concentration of 1 to 5 mM. Using a wide-bore syringe, about 1-

1.5 mL is taken into a syringe and slowly dispensed onto a square slide. The slide is then covered with a watch glass and left to dry for 2-4 hours.

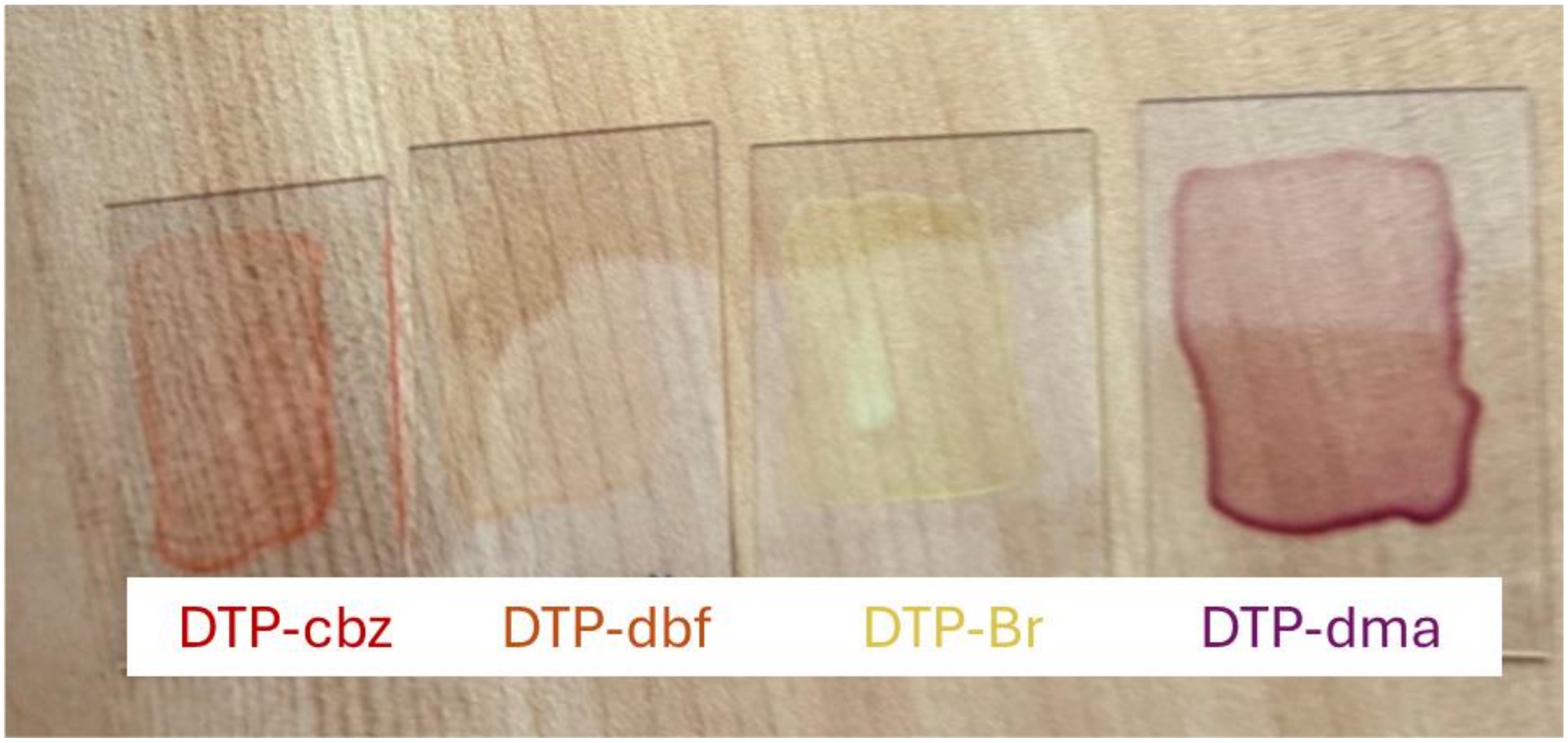

Figure 55. Images of PMMA films with each chromophore mixed in and dried.

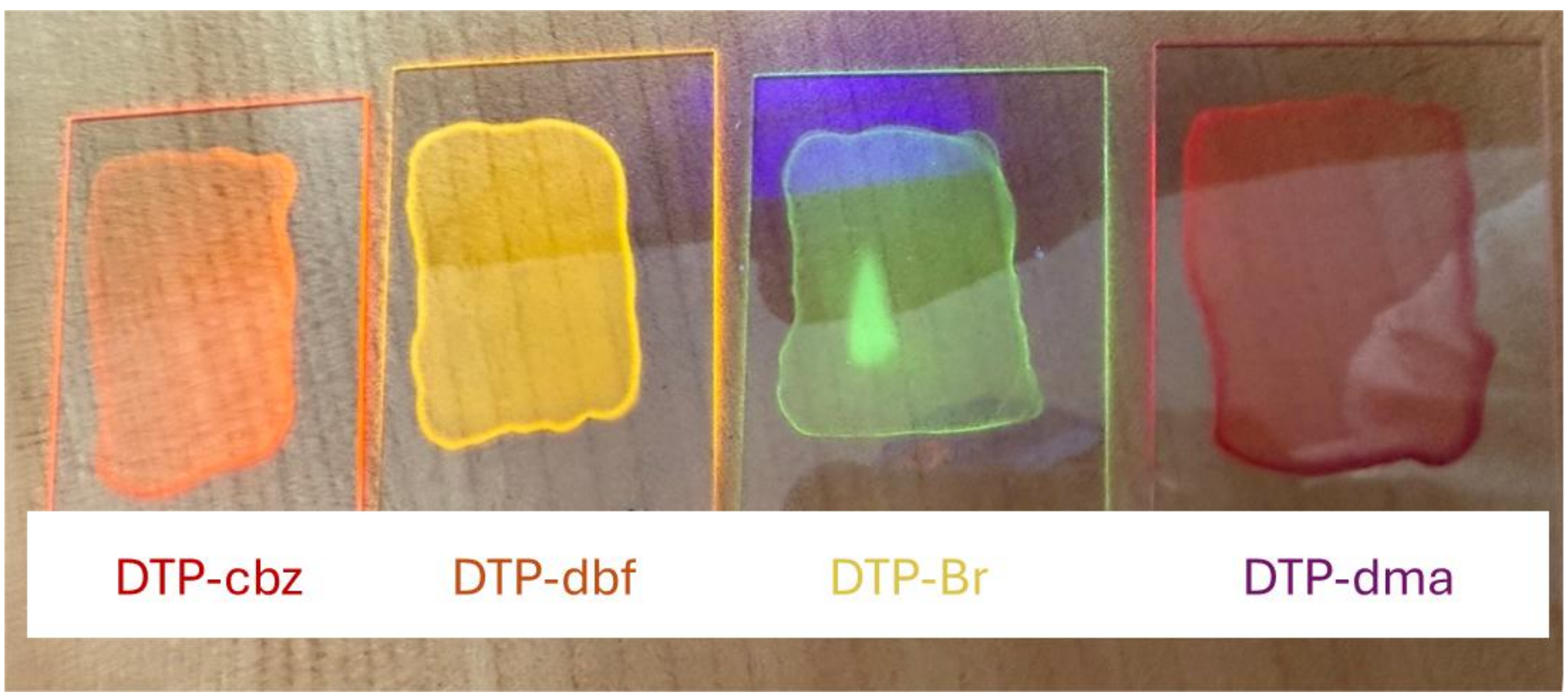

Figure 56. Images of PMMA films with each chromophore mixed in. Irradiated with 365 nm light.

References

(1) Brown, S.J.; Zhao, J.; Forehand, E.; Dobrzycki, L.; Roy, R.; Hasan, A M M.; Ding, W.; Schaack, C.; Evans, A. M. *J. Am. Chem. Soc.* **2025**, 147, 4, 3769-3775.
(2) Arroyave, F. A.; Richard, C.A.; Reynolds, J. R. *Org. Lett.* **2012**, 14, 24, 6138-6141.
(3) Neese, F. The ORCA Program System. *WIREs Comput Mol Sci* **2012**, 2 (1), 73–78. https://doi.org/10.1002/wcms.81.

(4) Neese, F. An Improvement of the Resolution of the Identity Approximation for the Formation of the Coulomb Matrix. *J. Comput. Chem.* **2003**, 24 (14), 1740–1747. https://doi.org/10.1002/jcc.10318.
(5) Neese, F. Software Update: The ORCA Program System—Version 5.0. *WIREs Comput Mol Sci* **2022**, 12 (5), e1606. https://doi.org/10.1002/wcms.1606.
(6) Neese, F. The SHARK Integral Generation and Digestion System. *J. Comput. Chem.* **2023**, 44 (3), 381–396. https://doi.org/10.1002/jcc.26942.
(7) Garcia-Ratés, M.; Neese, F. Effect of the Solute Cavity on the Solvation Energy and Its Derivatives within the Framework of the Gaussian Charge Scheme. *J. Comput. Chem*. **2020**, 41 (8), 922–939. https://doi.org/10.1002/jcc.26139.
